# Targeting EZH2-driven cholesterol metabolic vulnerability through Napabucasin suppresses ovarian cancer metastasis

**DOI:** 10.1038/s41419-026-08894-9

**Published:** 2026-06-27

**Authors:** Mingjun Ma, Chao Wang, Yue Zhang, Shanshan Cheng, Jiani Yang, Yejun Zou, Xiu Tian, Sijia Gu, Jianxiao Li, Weiwei Cao, Chao Huang, Yaodi Shao, Yaqian Zhao, Yutong Gao, Yilin Liu, Wen Pang, Shuo Shi, Hui Ding, Minghai Zhang, Yifei Cai, Yongsong Wu, Renhao Xue, Xiawei Cheng, Chen Chu, Jindan Sheng, Yu Wang

**Affiliations:** 1 Department of Gynecology, Shanghai Key Laboratory of Maternal Fetal Medicine, Shanghai Institute of Maternal-Fetal Medicine and Gynecologic Oncology, Clinical and Translational Research Center, Shanghai First Maternity and Infant Hospital, School of Medicine, Tongji University, Shanghai, China; 2 State Key Laboratory of Bioreactor Engineering, Optogenetics & Synthetic Biology Interdisciplinary Research Center, Shanghai Frontiers Science Center of Optogenetic Techniques for Cell Metabolism, School of Pharmacy, East China University of Science and Technology, Shanghai, China; 3 Department of Cancer Biology, Dana–Farber Cancer Institute, Boston, MA USA

**Keywords:** Targeted therapies, Preclinical research

## Abstract

Ovarian cancer represents the most lethal gynecologic malignancy, with tumor metastasis being the primary contributor to patient mortality. EZH2, frequently overexpressed in various cancers, has been implicated in promoting metastatic progression through metabolic dysregulation. However, the mechanistic basis by which EZH2 reprograms cholesterol metabolism to facilitate ovarian cancer metastasis remains poorly defined. In this study, we demonstrated that EZH2 is highly expressed in both primary and metastatic ovarian cancer tissues, correlating positively with poor clinical prognosis. Genetic silencing of EZH2 significantly suppressed tumor proliferation and metastatic dissemination. Mechanistically, EZH2 overexpression activated the NF-κB-Rap1A signaling axis and orchestrated cholesterol metabolic reprogramming by activating SREBP2, while repressing TMED10, to promote ovarian cancer metastasis. Furthermore, we identified Napabucasin as a potent suppressor of EZH2/Rap1A axis and cholesterol metabolism. Notably, Napabucasin effectively inhibited ovarian cancer metastasis in vivo. Collectively, our findings elucidate a previously unrecognized mechanism by which EZH2 governs metastatic progression through cholesterol metabolic rewiring and propose Napabucasin as a promising therapeutic strategy for ovarian cancer, particularly in tumors with EZH2 hyperactivation.

## Introduction

Ovarian cancer ranks as the second most lethal gynecologic malignancy globally, with 324,398 new cases and 206,839 deaths recorded in 2022 [1]. The anatomical positioning of the ovaries and the aggressive biology of epithelial carcinomas impede early detection at curable stages [2]. resulting in over 70% of patients presenting with advanced disease at diagnosis. These individuals face poor prognoses (5-year survival: 25–30%), high recurrence rates (>70%), and limited therapeutic options [3]. Primary ovarian tumors metastasize via intraperitoneal dissemination, lymphatic spread, or hematogenous routes, with intraperitoneal dissemination dominating in >90% of advanced cases [4]. This process manifests as peritoneal carcinomatosis and malignant ascites, hallmarks of late-stage disease [5]. Metastatic implants preferentially colonize the omentum and intestinal serosa, driving malignant bowel obstruction—a leading cause of recurrence-associated mortality [6, 7]. Despite therapeutic advancements, the molecular drivers of ovarian cancer metastasis remain poorly characterized, critically hindering the development of targeted therapies for metastatic progression.

EZH2 resides at chromosomal locus 7q35, comprises 20 exons, and encodes a 746-amino-acid protein [8]. As the core catalytic subunit of polycomb repressive complex 2 (PRC2), EZH2 modulates downstream genes and proteins through PRC2-dependent methylation, PRC2-independent methylation, or mechanisms independent of both PRC2 and methylation [9]. It is frequently overexpressed across a spectrum of cancers, where it plays a pivotal role in tumorigenesis, progression, and metastatic dissemination [10]. In ovarian cancer, EZH2 overexpression is observed in approximately 66% of primary epithelial tumors [11]. with its expression levels strongly correlating with advanced disease stage, metastatic potential, and tumor-associated angiogenesis [12, 13]. TMED10, a member of the transmembrane p24 trafficking protein family, localizes to the ER-Golgi intermediate compartment (ERGIC) and is found within COPI-coated vesicles [14]. It functions by oligomerizing to form protein channels that facilitate the transmembrane transport of unconventionally secreted proteins (UPS) into the ERGIC lumen [15]. Dysregulated cholesterol biosynthesis, a hallmark of many cancers, occurs primarily in the endoplasmic reticulum through energy-intensive reactions [16, 17]. Reprogrammed cholesterol metabolism significantly contributes to cancer progression by enhancing cellular proliferation, migration, and invasion [16]. Despite these insights, the interplay between EZH2 and TMED10 and their regulatory roles in cholesterol metabolism within ovarian cancer remains poorly understood.

Here, we systematically delineate the oncogenic role of EZH2 in ovarian cancer through comprehensive in vitro and in vivo studies. Through these, we found that EZH2 hyperactivation promotes aberrant cholesterol metabolism by transcriptionally upregulating SREBP2, a master regulator of cholesterol biosynthesis genes, while suppressing TMED10 to enhance cholesterol transmembrane transport. This dual regulatory axis fuels malignant phenotypes, including proliferation and metastatic dissemination. Furthermore, we reveal synergistic therapeutic efficacy between cholesterol synthesis inhibitors (e.g., pravastatin) and EZH2-targeted agents in ovarian cancer cells. We identify Napabucasin as a potent suppressor of EZH2-driven cholesterol metabolic rewiring through high-throughput drug screening and functional validation. Napabucasin exhibits robust anti-tumor activity in EZH2-high ovarian tumors in vivo, highlighting its translational potential as a precision therapeutic strategy.

## Results

### EZH2 and TMED10 are highly expressed in ovarian cancer

**Fig. 1:**
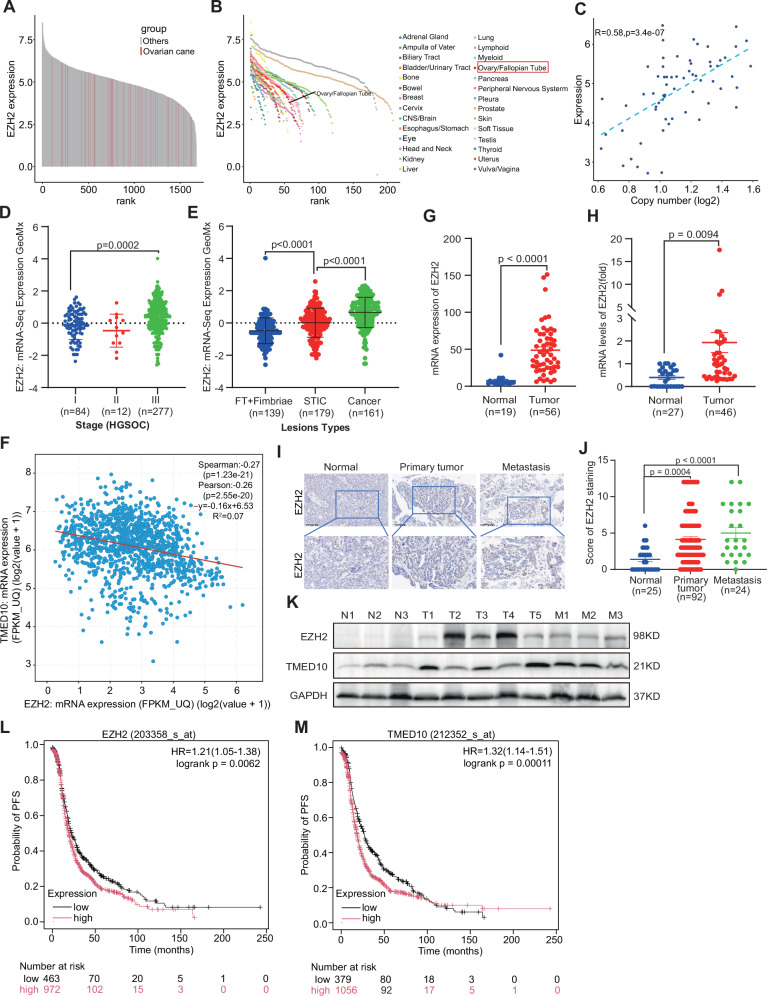
High EZH2 and TMED10 expression in ovarian cancer. **A** All cell lines annotated in the Cancer Cell Line Encyclopedia (CCLE) were arranged according to EZH2 transcript levels from the highest (left) to the lowest (right). Each line represents a different cell line. Ovarian cancer cell lines are denoted by red lines. **B** All cell lines annotated in the CCLE were grouped according to the cancer type. For each cancer type, cell lines were arranged according to the levels of EZH2 transcripts, from highest to lowest. Each dot represents a different cell line. **C** Correlation between the EZH2 gene copy number and gene expression in ovarian cancer cell lines from the CCLE. Each dot represents a different cell line. Pearson correlation coefficient and *p* value are shown. EZH2 expression in different stages of HGSOC (**D**) and different stages of ovarian cancer progression (normal Fallopian Tube/Fimbria, STIC, and invasive ovarian carcinoma; **E**) in the cBioPortal data (id=Ovary/Fallopian_OC_2024). **F** Correlation between the EZH2 and TMED10 gene expression (normalized FPKM) in all cancers from the ICGC/TCGA datasets in cBioPortal (id=pancan_pcawg_2020). RNA sequencing (RNA-Seq) analysis of EZH2 mRNA expression in 19 normal tissues and 56 ovarian cancer (OC) tissues (**G**) and RT-qPCR validation of EZH2 mRNA levels in 27 normal and 46 OC tissues (**H**). **I**, **J** IHC analysis of EZH2 protein expression in ovarian tissues. Representative images show EZH2 staining in normal, primary tumor, and metastatic tissues; scale bar, 100 μm (**I**). The dot plot quantifies differential EZH2 expression across normal (*n* = 25), primary tumor (*n* = 92), and metastatic (*n* = 24) cohorts, analyzed in a single experimental (**J**). Data are mean ± s.d.; statistical significance determined by a two-tailed unpaired t-test. **K** EZH2 and TMED10 protein levels in three normal, five tumors, and three metastatic tissues from patients. Images represent results from two independent experiments. Kaplan–Meier progression-free survival curve of patients stratified by EZH2 (**L**) or TMED10 (**M**) expression from the TCGA OV cohort. For (**D**, **E**, **G**, **H**, **J**), Data represent the mean ± s.d., and statistical analyses were performed using two-tailed unpaired t-tests.

To investigate the molecular drivers of ovarian cancer pathogenesis, we found that ovarian cancer cell lines exhibit relatively high expression levels of EZH2 and TMED10 compared to cell lines derived from other tumor types (Fig. 1A, B and Supplementary Fig. 1A, B), by analyzing transcriptomic data from over 1000 human cancer cell lines in the Cancer Cell Line Encyclopedia (CCLE).

A positive correlation was observed between gene copy number and transcript levels for both EZH2 and TMED10 in these cell lines (Fig. 1C and Supplementary Fig. 1C), suggesting that gene amplification may contribute to their expression. By analyzing cBioPortal data, we observed predominant gene amplification of EZH2 in ovarian cancer, with more pronounced amplification in advanced-stage disease (Supplementary Fig. 1D). Additionally, EZH2 mRNA expression demonstrated a progressive increase with advancing FIGO stages in high-grade serous ovarian carcinomas (HGSOC). Its expression increased incrementally during the pathological progression from normal fallopian tube to invasive ovarian carcinoma, whereas TMED10 mRNA levels exhibited no significant alterations (Fig. 1D, E and Supplementary Fig. 1E). In addition, stratified analysis of 88 normal tissues and 427 ovarian cancer specimens from the GDC cohort demonstrates elevated EZH2 and TMED10 expression in tumor tissues (Supplementary Fig. 1F). To systematically evaluate the expression landscape across malignancies, we interrogated TCGA datasets encompassing both unpaired and matched tumor-normal cohorts to evaluate its expression landscape systematically across malignancies. Multi-omics analysis revealed significant overexpression of EZH2 and TMED10 in diverse cancer types (Supplementary Fig. 1G).

Next, we performed RNA sequencing on 19 normal tissues and 56 ovarian cancer specimens. Comparative analysis revealed that EZH2 expression was significantly upregulated in tumor tissues (Fig. 1G). Subsequent RT-qPCR validation in 27 normal tissues and 46 ovarian cancer tissues confirmed marked upregulation of EZH2 mRNA in malignant tissues, while there was no significant difference in TMED10 expression (Fig. 1H and Supplementary Fig. 1I). Interestingly, we observed a significantly negative correlation between EZH2 and TMED10 transcript levels across all tumors and ovarian cancer accessed via cBioPortal data (Fig. 1F and Supplementary Fig. 1H). To comprehensively validate these observations, we employed orthogonal methodologies to assess EZH2 expression in patient-derived samples. Immunohistochemical (IHC) staining of 25 normal tissues and 92 ovarian cancer tissues (including 24 metastatic lesions) revealed significantly higher EZH2 protein intensity scores in tumor and metastatic specimens compared to normal tissues (Fig. 1I, J). Western blot analysis of representative subsets (three regulars, five primary tumors, and three metastatic tissues) demonstrated substantially elevated EZH2 and TMED10 protein levels in tumor and metastatic specimens (Fig. 1K). Complementary in vivo investigations utilizing our previously established murine model [18]. revealed a progressive increase in EZH2 expression during tumorigenesis (Supplementary Fig. 1J).

To further elucidate the prognostic implications of EZH2 and TMED10 expression patterns, we demonstrated that patients with elevated EZH2 and TMED10 expression exhibited poorer PFS by Kaplan–Meier survival analyses conducted through online platforms (Fig. 1L, M). Collectively, these findings establish that EZH2 is inversely correlated with TMED10 expression, and they are both overexpression in ovarian cancer.

Moreover, we used the cBioPortal TCGA high-grade serous ovarian cancer cohort (hgsoc_tcga_gdc), integrating RNA-seq expression, BRCA1/2 mutation annotation, and clinical survival data at the patient level. Survival analyses were performed both in the full evaluable cohort and in the BRCA-WT subset, where EZH2 overexpression was most prominent. We compared the top versus bottom quartiles of EZH2 expression within the BRCA-WT subgroup, yielding 101 high-EZH2 and 101 low-EZH2 cases for overall survival analysis, and 40 high-EZH2 and 50 low-EZH2 cases for progression-free survival analysis. In this subgroup-restricted analysis, EZH2 showed only a borderline trend with overall survival and no association with progression-free survival.

We further evaluated EZH2 as a continuous variable in BRCA-WT patients. In univariable Cox regression, EZH2 expression was not significantly associated with overall survival or progression-free survival. We also tested whether the prognostic effect of EZH2 differed by BRCA status in the full cohort. In the full cohort, the EZH2 × BRCA status interaction term was not significant for either overall survival or progression-free survival. In the age-adjusted interaction model, the interaction likewise remained non-significant for overall survival and progression-free survival. In addition to repeating the survival analyses within BRCA-WT tumors, we performed transcriptome-level pathway analysis in the same BRCA-WT subgroup using the cBioPortal TCGA high-grade serous ovarian cancer cohort. Comparing the highest and lowest quartiles of EZH2 expression, Hallmark GSEA demonstrated that EZH2-high BRCA-WT tumors were enriched for proliferation- and cell-cycle-associated programs, including E2F targets, G2M checkpoint, MYC targets, mitotic spindle, DNA repair, and mTORC1 signaling (Supplementary Fig. 1K–M).

### EZH2 and TMED10 drive malignant progression in ovarian carcinoma

**Fig. 2:**
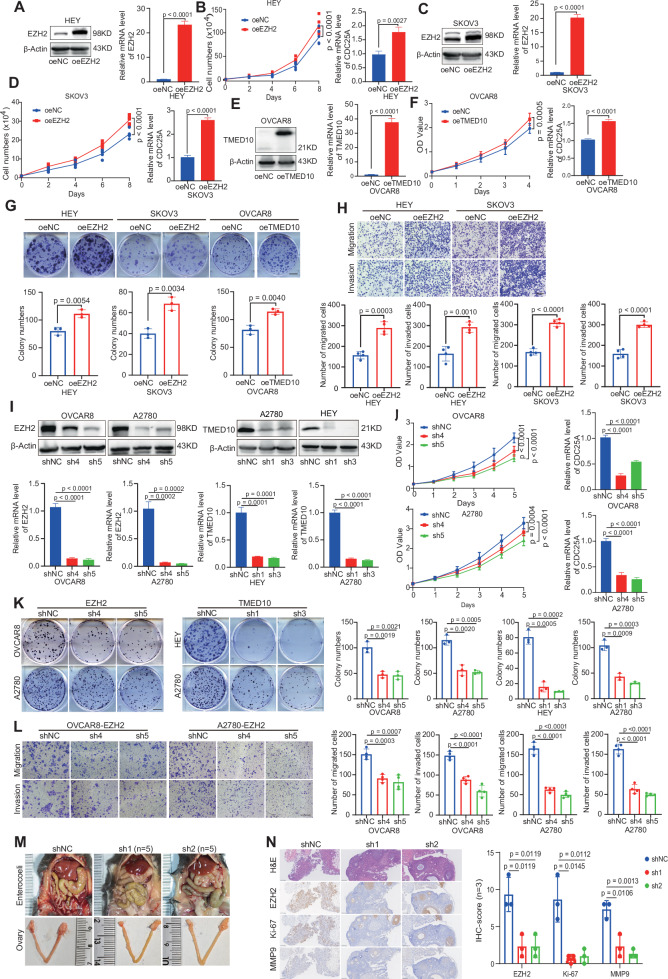
EZH2 and TMED10 drive malignant progression in ovarian carcinoma. Immunoblotting and RT-qPCR analysis of EZH2 overexpression (OE) levels in HEY (**A**) and SKOV3 (**C**) cells transfected with OE or empty vector control (oeNC). Images are representative of at least three independent experiments. Cell counts and CDC25A mRNA expression of HEY (**B**) and SKOV3 (**D**) oeEZH2 and oeNC cells over 8 days. Data from three independent cultures. **E** Immunoblotting and RT-qPCR analysis of TMED10 overexpression (OE) levels in OVCAR8 cells transfected with OE or empty vector control (oeNC). Images are representative of at least three independent experiments. **F** CCK-8 and CDC25A mRNA expression of OVCAR8-oeTMED10 and oeNC cells over 4 days. Data from three independent cultures. **G** Clonogenic capacity of HEY, SKOV3-oeEZH2, and OVCAR8-oeTMED10 versus oeNC cells after 14 days; scale bar, 200 μm; data from three independent cultures. **H** Migration and invasion ability of HEY and SKOV3-oeEZH2 versus oeNC cells. Representative images of migrated and invaded cells are shown; scale bar, 100 μm. Data are from three independent cultures. **I** Immunoblotting and RT-qPCR analysis of EZH2 knockdown (left) in OVCAR8 and A2780 cells, and TMED10 knockdown (right) in A2780 and HEY cells using two independent shRNAs or non-targeting control (shNC). Images are representative of at least three experiments. **J** Viability of OVCAR8 and A2780-shEZH2 versus shNC cells over 5 days. Data from three independent cultures. **K** Clonogenic capacity of OVCAR8 and A2780-shEZH2 compared to shNC cells (left), HEY and OVCAR8-shTMED10 compared to shNC cells after 14 days; scale bar, 200 μm; data from three independent cultures. **L** Migration and invasion assays of OVCAR8 and A2780-shNC and shEZH2 cells. Representative images of migrated and invaded cells are shown; scale bar, 100 μm; data from three independent cultures. **M** Metastatic burden in ID8 shEZH2 and shNC tumor-bearing mice (*n* = 5 per group; single experiment). Representative images of ovarian tumor metastases. **N** H&E staining and IHC analysis of EZH2, Ki-67, and MMP9 in ID8 shEZH2 and shNC tumor tissues (*n* = 3 independent tumors per group). Scale bar, 100 μm. For (**A**–**N**), Data represent the mean ± s.d., and statistical analyses were performed using two-tailed unpaired t-tests.

To investigate the functional impact of EZH2 and TMED10 in ovarian cancer cells, we first assessed their expression profiles across multiple cell lines using Western blot and RT-qPCR. Comparative analyses revealed elevated EZH2 mRNA and protein levels in A2780, OVCAR8, and ES-2 cells relative to normal ovarian epithelial cells (IOSE), while HEY, SKOV3, and OV90 cells exhibited lower baseline expression. By contrast, TMED10 exhibited relatively low expression in OVCAR8 and SKOV3 cells, whereas higher expression was observed in HEY and A2780 cell lines. (Supplementary Fig. 2A, B). To establish causality, we stably overexpressed endogenous EZH2 in these low-expressing cell lines (HEY, SKOV3, OV90), achieving significant upregulation at both transcriptional and protein levels compared to empty vector controls (Fig. 2A, C and Supplementary Fig. 2C). Functional characterization of EZH2-driven oncogenicity revealed pleotropic pro-tumorigenic effects. Cell counts, CCK-8 viability assays combined with RT-qPCR-based CDC25A mRNA quantification demonstrated that EZH2 overexpression (OE) robustly enhanced proliferation in HEY, SKOV3, and OV90 cells compared to empty vector control groups (Fig. 2B, D and Supplementary Fig. 2D, E). Consistent with this pattern, stable overexpression of TMED10 in OVCAR8 and SKOV3 cells also enhanced cellular proliferation compared to control cells (Fig. 2E, F and Supplementary Fig. 2F, G). Colony formation assays further corroborated these findings, with OE cells exhibiting an increase in clonogenic capacity across all tested lines (Fig. 2G and Supplementary Fig. 2H). Given the established correlation between EZH2 overexpression and metastatic burden in ovarian cancer clinical specimens, we systematically evaluated its functional role in metastasis-associated phenotypes. Transwell migration and invasion assays revealed that EZH2-OE cells displayed markedly enhanced migratory and invasive capacities relative to controls (Fig. 2H and Supplementary Fig. 2I), mechanistically linking EZH2 to the aggressive dissemination traits underlying patient mortality. Similarly, TMED10 overexpression also promoted cell migration and invasion (Supplementary Fig. 2I),

To establish the functional necessity of EZH2 and TMED10 in tumor progression, we engineered endogenous EZH2 and TMED10 knockdown (KD) models in (EZH2: A2780, OVCAR8, and ES-2; TMED10: A2780 and HEY) cell lines, which exhibit high baseline EZH2 and TMED10 expression. Transfection with EZH2 and TMED10-targeting shRNAs achieved robust post-transcriptional suppression compared to controls (Fig. 2I and Supplementary Fig. 2J). Subsequent cell proliferation assays revealed a reduction in proliferative capacity across KD lines versus controls (Fig. 2J and Supplementary Fig. 2K, L). Colony formation efficiency was markedly attenuated in KD cells, with over a 50% reduction in macroscopic colony counts (Fig. 2K and Supplementary Fig. 2M). Transwell matrices showed that EZH2 or TMED10 depletion significantly reduced migratory and invasive capacities in all tested lines (Fig. 2L and Supplementary Fig. 2N), inversely reflecting the pro-metastatic effects observed in OE models. We further performed RNA-seq analysis using OVCAR8-EZH2-shNC versus sh4/sh5 cells. GO enrichment analysis revealed that terms such as extracellular matrix organization, extracellular structure organization, and collagen-containing extracellular matrix were significantly enriched among the downregulated genes following EZH2 knockdown (Supplementary Fig. 3A). Subsequently, RT-qPCR analysis showed that the expression levels of MMP9 and MMP2 were increased upon EZH2 overexpression, whereas they were decreased following EZH2 knockdown (Supplementary Fig. 3B). Furthermore, immunoblotting revealed that EZH2 overexpression led to decreased protein expression of TIMP2, along with increased expression of MMP2 and MMP9. Conversely, endogenous EZH2 knockdown resulted in the opposite expression trends (Supplementary Fig. 3C). Furthermore, we employed a DOX‑inducible shRNA expression system. Immunoblotting confirmed the knockdown efficiency of EZH2 in A2780 and OVCAR8 cells following DOX induction (Supplementary Fig. 3D). CCK‑8 assays demonstrated that DOX‑induced cells exhibited reduced proliferative capacity compared to the DOX‑negative control group (Supplementary Fig. 3E). Transwell assays further showed that the numbers of migrating and invading A2780 and OVCAR8 cells were decreased after DOX induction (Supplementary Fig. 3F).

To explore whether EZH2 knockdown affects tumor growth and metastasis in vivo, we established an orthotopic ID8 murine model with stable endogenous EZH2 knockdown (shEZH2) versus non-targeting controls (shNC) (Supplementary Fig. 2O). Longitudinal monitoring revealed that shEZH2-bearing mice exhibited a modest reduction in body weight gain over 7 weeks compared to shNC cohorts (Supplementary Fig. 2P), indicative of diminished tumor-associated cachexia. Meanwhile, shEZH2 mice displayed a marked reduction in ascites volume and decreased metastatic burden across peritoneal surfaces, intestines, and mesenteric tissues compared to shNC groups. Ovarian tumor mass in shEZH2 mice was also reduced (Fig. 2M). Moreover, H&E and immunohistochemical staining revealed decreased staining intensity of Ki-67 and MMP9, markers of cell proliferation and migration/invasion, respectively, in ovarian tissues from EZH2-KD mice (Fig. 2N). To further clarify the effect of EZH2 knockdown on tumor metastasis in vivo, ID8-shNC and ID8-sh1/sh2 cells were orthotopically injected into the ovaries of C57BL/6 mice. Consistent with our in vitro findings, EZH2 knockdown led to slower body weight gain and reduced numbers of intraperitoneal metastatic nodules and ascites (Supplementary Fig. 9A–D). These findings mechanistically position EZH2 as a central orchestrator of malignant progression, whose therapeutic targeting may disrupt both primary tumor expansion and metastatic dissemination in ovarian cancer.

### EZH2 upregulation activates the NF-κB-Rap1A signaling

**Fig. 3:**
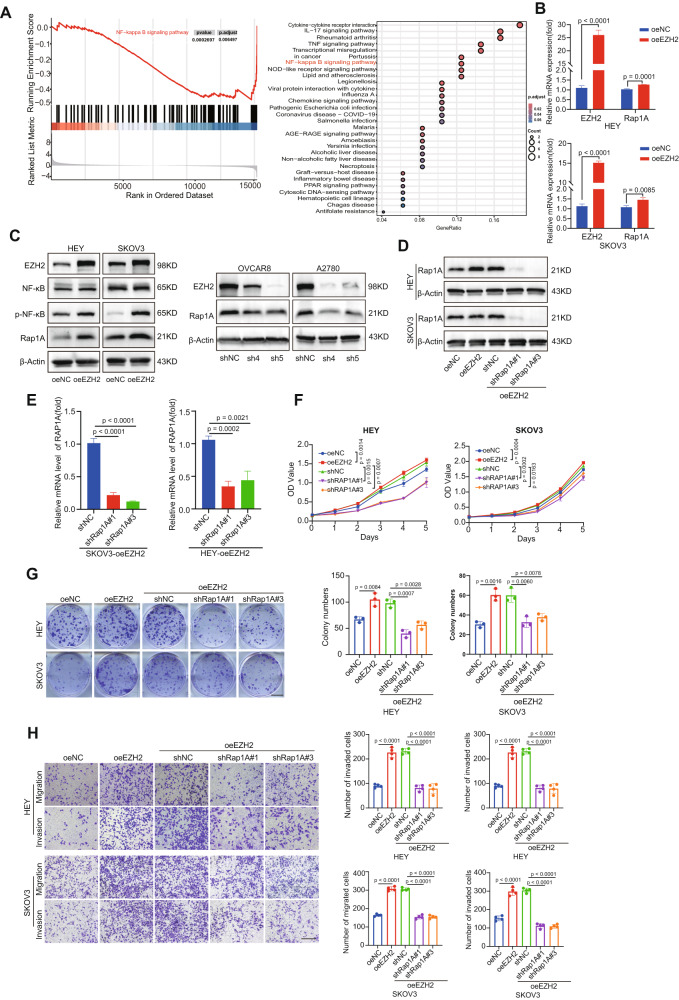
EZH2 upregulation activates the NF-κB-Rap1A signaling. **A** GSEA (left) and KEGG (right) analysis of differentially expressed genes downregulated with EZH2 in OVCAR8 cells. **B** RT-qPCR analysis of EZH2 and Rap1A mRNA levels in EZH2-overexpressing HEY and SKOV3 cells. Results from three independent experiments. **C** Immunoblotting analysis of p-NF-κB, NF-κB, and Rap1A protein levels in HEY and SKOV3 cells overexpressing EZH2 (left), as well as Rap1A protein levels in OVCAR8 and A2780 cells with EZH2 knockdown (right). Blots are representative of at least three independent experiments. Immunoblotting (**D**) and RT-qPCR (**E**) validation of Rap1A knockdown using two independent shRNAs or non-targeting control (shNC) in EZH2-overexpressing HEY and SKOV3 cells. Blots are representative of at least two experiments. **F** Viability of EZH2-OE HEY and SKOV3 cells with Rap1A knockdown (shRNA) or shNC controls over 5 days. Data from three independent cultures. **G** Clonogenic capacity of HEY and SKOV3 EZH2-OE cells with Rap1A knockdown after 14 days; scale bar, 200 μm; data from three independent cultures. **H** Migration and invasion ability of HEY and SKOV3 EZH2-OE cells with Rap1A knockdown. Representative images of migrated and invaded cells are shown; scale bar, 100 μm; data from three independent cultures. For (**B**, **E**–**H**), Data represent the mean ± s.d., and statistical analyses were performed using two-tailed unpaired t-tests.

To further explore the molecular pathways through which EZH2 regulates cellular functions, we performed enrichment analysis on the downregulated differentially expressed genes following EZH2 knockdown in our RNA‑seq data. This analysis revealed significant enrichment of the NF‑κB pathway (Fig. 3A). KEGG pathway analysis of differentially expressed genes between TMED10‑high and TMED10‑low groups in the TCGA database showed prominent enrichment of the Rap1 signaling pathway (Supplementary Fig. 4A). It has been reported that EZH2 activates the NF‑κB signaling pathway by regulating RelB transcription, thereby increasing Rap1 protein expression [19–25]. Accordingly, analysis of differentially expressed genes in the EZH2‑high group from TCGA revealed upregulation of RAP1A and downregulation of TMED10 upon EZH2 overexpression, and a positive correlation between EZH2 and RAP1A expression was observed (Supplementary Fig. 4B). Further validation showed that RAP1A mRNA levels were increased upon EZH2 overexpression and decreased upon EZH2 knockdown (Fig. 3B and Supplementary Fig. 4C). Immunoblotting demonstrated that EZH2 overexpression elevated the protein levels of phosphorylated NF‑κB (Ser536) and RAP1A, whereas EZH2 knockdown reduced RAP1A protein levels (Fig. 3C and Supplementary Fig. 4E). To further investigate this effect, nuclear‑cytoplasmic fractionation assays showed that EZH2 overexpression predominantly promoted the upregulation of RAP1A protein in the cytoplasmic compartment (Supplementary Fig. 4D). The molecular weight of p‑NF‑κB protein was confirmed by immunoblotting (Supplementary Fig. 4D). Subcellular fractionation further revealed that both EZH2 and NF‑κB were mainly localized in the nucleus, while phosphorylated NF‑κB was detected in both the cytoplasmic and nuclear fractions (Supplementary Fig. 4D). Treatment of EZH2‑overexpressing cells with the IκBα/NF‑κB inhibitor BAY 11‑7082 enhanced pathway suppression and reduced p‑NF‑κB levels compared to control cells (Supplementary Fig. 4D).

To further investigate whether EZH2 directly regulates the NF‑κB pathway, we first performed immunoblotting. EZH2 overexpression increased the protein levels of H3K4me3, H3K9me3, H3K27me3, and H3K36me3, whereas EZH2 knockdown decreased their levels, with the most pronounced change observed for H3K27me3 (Supplementary Fig. 4F). We then performed ChIP‑seq in HEY‑oeNC and HEY‑oeEZH2 cells. Compared with controls, EZH2 overexpression led to the identification of 2083 significantly differential peaks genome‑wide, including 561 upregulated and 1522 downregulated peaks. A volcano plot displays the top 20 most significantly differential peaks (Supplementary Fig. 4G), and a heatmap shows the genes associated with these peaks (Supplementary Fig. 4H). Notably, no direct binding of EZH2 to the NFKB1, RELA, or RAP1A genes was observed, suggesting that EZH2 may not regulate the expression of these genes by directly binding to their promoters.

To interrogate Rap1A’s necessity in EZH2-mediated tumor progression, we performed genetic rescue experiments in EZH2-OE HEY, SKOV3, and OV90 stable lines. Rap1A knockdown (KD) was achieved via shRNA transfection. We validated the endogenous reduction in Rap1 protein and mRNA levels compared to scramble controls (Fig. 3D, E and Supplementary Fig. 4I). Next, CCK-8 assays demonstrated that Rap1A-KD reversed EZH2-OE-induced proliferation in HEY, SKOV3, and OV90 cells (Fig. 3F and Supplementary Fig. 4J). Colony formation assays confirmed that Rap1A-KD abrogated EZH2-driven clonogenicity across all lines (Fig. 3G and Supplementary Fig. 4K). Finally, Transwell matrices showed that Rap1A-KD attenuated EZH2-enhanced migration and invasion capacities of HEY, SKOV3, and OV90 cells (Fig. 3H and Supplementary Fig. 4L). These rescue experiments mechanistically position Rap1A as the dominant downstream effector of EZH2’s pro-tumorigenic program.

### EZH2 orchestrates cholesterol metabolic reprogramming

**Fig. 4:**
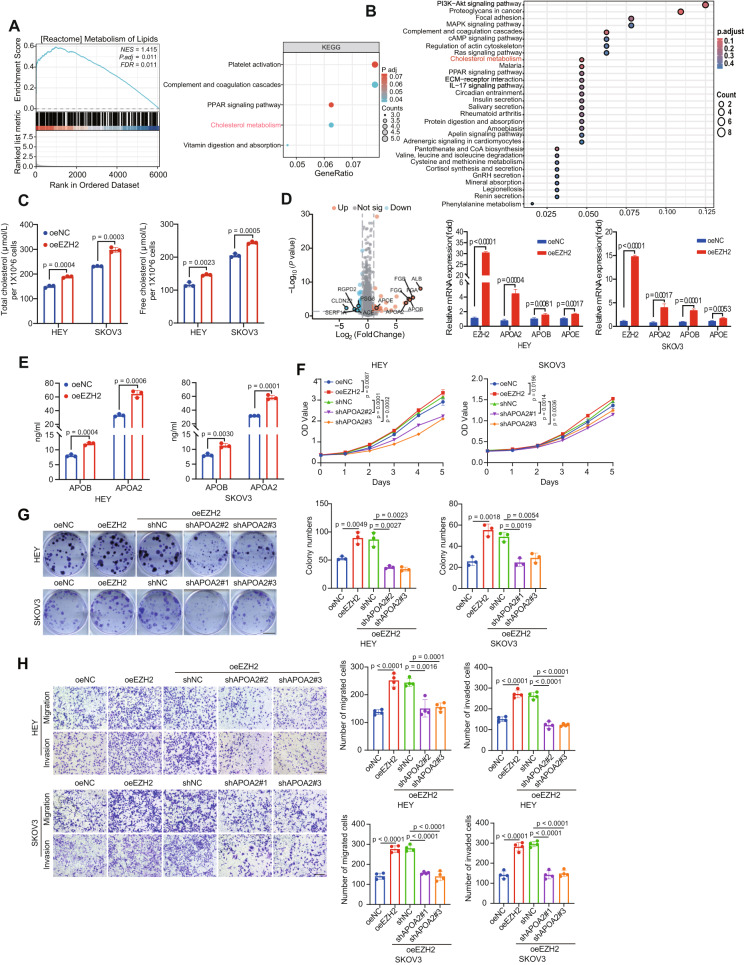

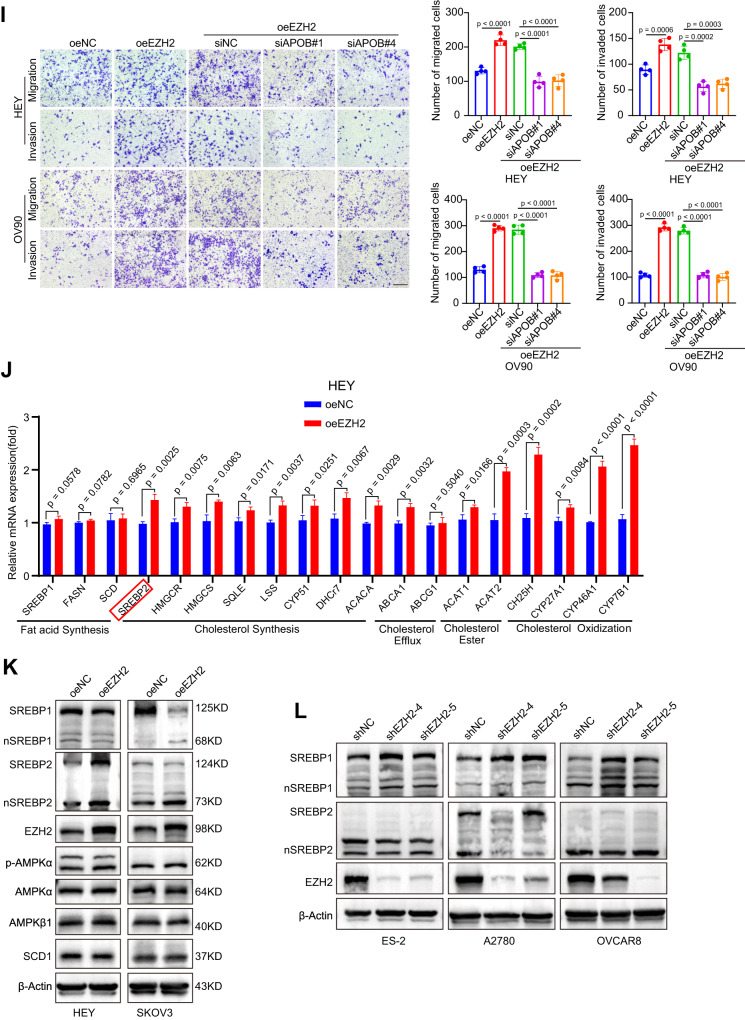
EZH2 orchestrates cholesterol metabolic reprogramming. **A** Gene Set Enrichment Analysis (GSEA) of upregulated pathways in HEY EZH2-OE cells; NES, normalized enrichment score. Data were analyzed using a two-sided permutation test, with *P* values adjusted using the Benjamini-Hochberg method (left); Kyoto Encyclopedia of Genes and Genomes (KEGG) upregulated signaling set from RNA-seq of HEY-OE versus control cells. *n* = 3 independent cell cultures (right). **B** Downregulation analysis of genes after knockdown of EZH2 in OVCAR8 cells via KEGG. **C** Total cholesterol and free cholesterol levels in EZH2-overexpressing HEY and SKOV3 cells. Data are derived from three independent biological replicates. **D** Volcano plots of significantly affected (padj<0.05, |log₂FC| > 1) genes in HEY-oeEZH2 cells relative to oeNC cells as revealed by RNA-seq. Both oeEZH2 and oeNC groups contain three biological replicates, assessed as one experiment. *P* values were calculated using R v4.0.3 software. Data were analyzed by a two-tailed unpaired t-test (left). RT-qPCR analysis of APOB, APOA2, and APOE mRNA levels in EZH2-OE HEY and SKOV3 cells. Results from three independent experiments (right). **E** APOB and APOA2 levels in conditioned media collected from HEY and SKOV3 cells overexpressing EZH2 following 48-h culture, as determined by ELISA. Data are derived from three independent biological replicates. **F** Viability of EZH2-OE HEY (left) and SKOV3 (right) cells transfected with two independent shAPOA2 constructs or non-targeting control (shNC) over 5 days. Data from three independent cultures. **G** Clonogenic capacity of HEY and SKOV3 EZH2-OE cells with APOA2 knockdown after 14 days; scale bar, 200 μm; data from three independent cultures. **H** Migration and invasion ability of HEY and SKOV3 EZH2-OE cells with APOA2 knockdown and shNC cells. Representative images of migrated and invaded cells are shown; scale bars, 100 μm; cell cultures from three independent experiments. **I** Migration and invasion ability of HEY and OV90 EZH2-OE cells with siAPOB and siNC cells. Representative images of migrated and invaded cells are shown; scale bars, 100 μm; cell cultures from three independent experiments. **J** RT-qPCR analysis of mRNA expression levels of cholesterol synthesis, efflux, esterification, and oxidation-related genes in EZH2-OE HEY cells. Data are from three independent biological replicates. **K** Immunoblotting analysis of SREBP1 (full-length), nSREBP1 (nuclear SREBP1), SREBP2 (full-length), nSREBP2 (nuclear SREBP2), p-AMPKα, AMPKα, AMPKβ1, and SCD1 protein levels in HEY and SKOV3 cells overexpressing EZH2. Representative immunoblots from three independent experiments are shown. **L** Immunoblotting analysis of SREBP1 (full-length), nSREBP1, SREBP2 (full-length), and nSREBP2 protein levels in ES-2, A2780 and OVCAR8 cells with EZH2 knockdown. Representative immunoblots from three independent experiments are shown. For (**C**–**J**), Data represent the mean ± s.d., and statistical analyses were performed using two-tailed unpaired t-tests.

Based on the previous findings, we further performed whole-transcriptome sequencing (RNA-seq) in HEY cells with stable EZH2 overexpression (OE). Comparative analysis identified 29 significantly upregulated and 42 downregulated genes (|log2FC | >1, *p*adj < 0.05). GSEA enrichment analysis of upregulated genes revealed significant enrichment of lipid metabolism and steroid metabolism pathways. Subsequently, KEGG pathway analysis demonstrated prominent enrichment of cholesterol metabolism (Fig. 4A, B and Supplementary Fig. 5A). At the same time, GO functional enrichment analysis revealed pronounced activation of glycerolipid metabolic process, lipid catabolic process, regulation of protein secretion, lipid transfer activity, and cholesterol transfer activity, etc., in OE cells (Supplementary Fig. 5A). In EZH2-OE cells, the mRNA levels of cholesterol synthesis-related genes (HMGCS, HMGCR, ACACA) were upregulated, as were those of cholesterol esterification and efflux-related genes ACAT2 and ABCA1 (Supplementary Fig. 5B). Consequently, quantification of intracellular cholesterol revealed significantly increased levels of both total and free cholesterol in EZH2-overexpressing HEY and SKOV3 cells. Conversely, EZH2 KD in A2780, OVCAR8, and ES-2 cells markedly reduced intracellular total and free cholesterol levels (Fig. 4C and Supplementary Fig. 5C).

Subsequent analysis of RNA-seq differentially expressed genes (DEGs) identified APOB and APOA2 as significantly altered candidate genes through volcano plot visualization (Fig. 4D). To validate the reliability of the RNA‑seq data, we additionally examined the expression of other significantly differentially expressed genes by RT‑qPCR and confirmed that the expression of the upregulated differentially expressed genes was increased upon EZH2 overexpression (Supplementary Fig. 5D). Concurrently, hierarchically clustered heatmaps displayed the top 19 upregulated and downregulated DEGs, and RT-qPCR confirmed EZH2-dependent upregulation of APOA2, APOB, and APOE in SKOV3 and HEY OE cells compared to controls (Fig. 4D and Supplementary Fig. 5D). Due to the critical roles of APOA2/APOB in cholesterol homeostasis transport, we investigated EZH2’s role in cholesterol metabolic rewiring. ELISA quantification revealed significant increases in extracellular APOB and APOA2 levels in EZH2-overexpressing (OE) HEY and SKOV3 cells versus controls (Fig. 4E). Conversely, EZH2 knockdown (KD) in A2780 and OVCAR8 cells marked reduced APOB and APOA2 secretion (Supplementary Fig. 5E). Thus, EZH2 overexpression promotes APOB and APOA2 expression and secretion. Building on transcriptomic profiling and RT-qPCR validation of EZH2-driven APOA2/APOB upregulation, we systematically examined their functional contributions through genetic rescue models. Stable APOA2 knockdown (KD) in EZH2-OE HEY, SKOV3, and OV90 lines achieved over 65% mRNA reduction compared to scramble controls (Supplementary Fig. 5F). CCK-8 assays revealed that APOA2-KD significantly reversed EZH2-OE-induced proliferation in HEY, SKOV3, and OV90 (Fig. 4F and Supplementary Fig. 5G). Colony formation assays confirmed that APOA2-KD abrogated EZH2-driven clonogenicity across all lines (Fig. 4G and Supplementary Fig. 5H). Finally, transwell assays demonstrated that APOA2-KD markedly suppressed EZH2-enhanced migration in all HEY and SKOV3 lines (Fig. 4H). Moreover, we knocked down APOB using siRNA in HEY and OV90 cells overexpressing EZH2. RT-qPCR confirmed the knockdown efficiency (Supplementary Fig. 5I). Transwell assays indicated that APOB depletion significantly reduced EZH2-enhanced migration and invasion (Fig. 4I). These findings position EZH2 as a master regulator of cholesterol metabolism in ovarian cancer, creating a protumor genic milieu through coordinated apolipoprotein secretion and sterol biosynthesis. And APOA2 and APOB as critical mediators of EZH2’s oncogenic program.

To elucidate the molecular mechanism underlying EZH2-driven cholesterol metabolic rewiring, we systematically examined its regulatory effects on sterol biosynthesis and transport pathways. RT-qPCR analysis revealed that in HEY and SKOV3 cells overexpressing EZH2, the mRNA levels of genes involved in cholesterol synthesis (SREBP2, HMGCR, HMGCS, CYP51, DHCR7, ACACA), cholesterol esterification (ACAT2), and cholesterol oxidation (CH25H, CYP27A1, CYP46A1) were significantly upregulated (Fig. 4J and Supplementary Fig. 5J). Correlation analysis via the cBioPortal database confirmed a statistically significant positive association between EZH2 and SREBF2, HMGCS1 expression in ovarian cancer (Supplementary Fig. 5K). Western blot analysis further demonstrated that in HEY and SKOV3 cells, EZH2-OE significantly upregulated the protein levels of the nuclear transcriptionally active nSREBP2, while downregulating full-length SREBP1 and nuclear nSREBP1. Additionally, EZH2-OE marginally increased p-AMPKα levels but did not alter AMPKβ1 or SCD1 levels (Fig. 4K). Conversely, in ES-2, A2780, and OVCAR8 cells with EZH2 knockdown, the protein levels of nuclear nSREBP2 were decreased, while those of full-length and nuclear nSREBP1 were increased (Fig. 4L). These data establish that EZH2 drives cholesterol metabolic reprogramming in ovarian cancer through SREBP2-dependent transcriptional activation of key synthesis genes while reciprocally suppressing lipogenic pathways via SREBP1 downregulation. The AMPK-independent nature of this regulation highlights EZH2’s role as a master coordinator of sterol homeostasis.

### EZH2 negatively regulates TMED10 to reprogram cholesterol metabolism

**Fig. 5:**
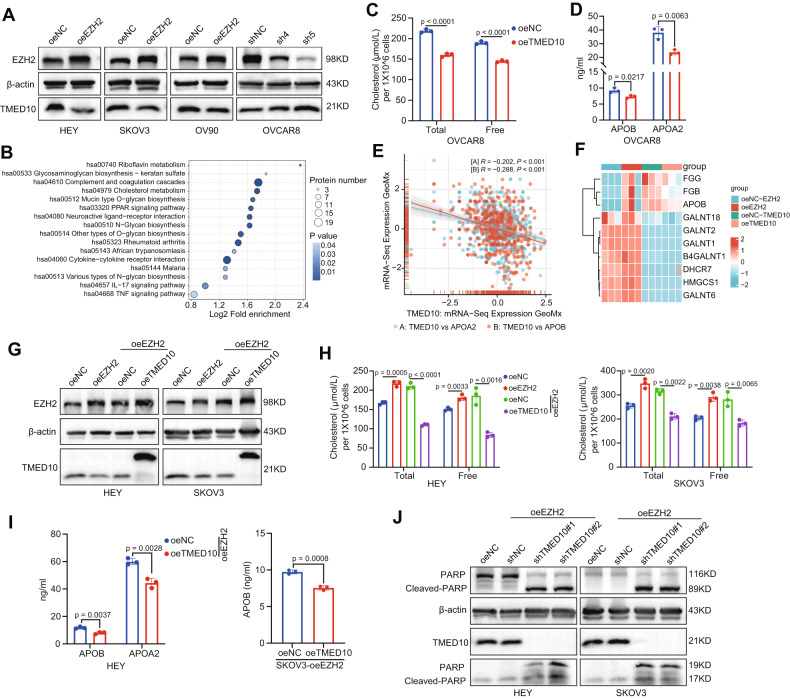
EZH2 negatively regulates TMED10 to reprogram cholesterol metabolism. **A** Immunoblotting analysis of EZH2 and TMED10 protein levels in HEY, SKOV3, and OV90 cells overexpressing EZH2, and OVCAR8 cells with EZH2 knockdown. Representative immunoblots from three independent experiments are shown. **B** Kyoto Encyclopedia of Genes and Genomes (KEGG) downregulated signaling from proteomics sequencing of cell culture supernatants with OVCAR8-OE versus control cells. *n* = 3 independent cell cultures. **C** Total cholesterol and free cholesterol levels in TMED10-OE OVCAR8 cells. Data are derived from three independent biological replicates. **D** APOB and APOA2 levels in conditioned media collected from OVCAR8 cells overexpressing TMED10 following 48-h culture, as determined by ELISA. Data are derived from three independent biological replicates. **E** Correlation between the TMED10 and APOB or APOA2 gene expression in ovarian cancer from the cBioPortal data (id=Ovary/Fallopian_OC_2024). **F** Heatmap of the genes upregulated in the RNA-seq of HEY EZH2-OE and oeNC but downregulated in the proteomics results of OVCAR8 TMED10-OE and oeNC cells. **G** Immunoblotting analysis of TMED10 overexpression in EZH2-OE HEY and SKOV3 cells. Representative immunoblots from three independent experiments are shown. **H** Intracellular total cholesterol and free cholesterol levels in EZH2-OE HEY and SKOV3 cells with TMED10 overexpression. Data represent three independent biological replicates. **I** ELISA analysis of APOB and APOA2 secretion levels in EZH2-OE HEY and APOB secretion levels in EZH2-OE SKOV3 cells with TMED10 overexpression. Data are from three independent biological replicates. **J** Immunoblotting analysis of PARP, Cleaved-PARP, and Cleaved-Caspase-3 levels in EZH2-OE HEY and SKOV3 cells with TMED10 knockdown. Representative immunoblots from two independent experiments are shown. For (**C**, **D**, **H**, **I**), Data represent the mean ± s.d., and statistical analyses were performed using two-tailed unpaired t-tests.

Given that EZH2 promotes the expression and secretion of apolipoproteins APOA2 and APOB, which facilitate cholesterol efflux, we explored how EZH2 drives cholesterol secretion from cells. TMED10, located in the ERGIC, regulates unconventional protein secretion (UCPS) for leaderless cargoes [26]. Moreover, TMED10 exhibited an inverse correlation with EZH2 expression. Consequently, we investigated the mechanistic basis of EZH2-mediated modulation of TMED10 expression and its functional role in cholesterol metabolism. We first demonstrated that EZH2 overexpression in HEY, SKOV3, and OV90 cells markedly suppressed TMED10 expression, whereas EZH2 knockdown in OVCAR8 cells led to elevated TMED10 protein levels evaluated by RT-qPCR and Western blot (Fig. 5A and Supplementary Fig. 6A).

To explore TMED10’s impact on protein secretion, we conducted proteomics sequencing on the culture supernatant of OVCAR8 cells overexpressing TMED10. Interestingly, KEGG pathway analysis identified significant suppression of cholesterol metabolism (Fig. 5B). COG/KOG enrichment analysis revealed that TMED10 overexpression downregulated proteins involved in signal transduction mechanisms and various substances transport and metabolism. Meanwhile, GO enrichment analysis revealed that TMED10-OE downregulated proteins involved in biological and metabolic process regulation compared to controls (Supplementary Fig. 6B). The intracellular cholesterol content was further determined TMED10 overexpression significantly reduced intracellular total and free cholesterol levels in OVCAR8 cells (Fig. 5C). And the supernatant of TMED10-OE cells showed reduced apolipoproteins like APOB, APOM and APOH (Supplementary Fig. 6C). Correlation analysis of ovarian cancer genomics data from the cBioPortal data revealed statistically significant inverse associations between TMED10 expression and transcript levels of APOA2, APOB, APOM, and APOE (Fig. 5E and Supplementary Fig. 6D). ELISA confirmed that TMED10-OE markedly decreased APOB and APOA2 secretion in OVCAR8 cells (Fig. 5D). Integrated analysis of RNA-seq profiles from EZH2-overexpressing cells and proteomic datasets from TMED10-OE models revealed that cholesterol metabolism regulators upregulated by EZH2 overexpression-including APOB, HMGCS, DHCR7, and GALNT2 (which modulates lipid metabolism via O-glycosylation)-were conversely downregulated following TMED10 overexpression. This inverse regulatory pattern was further validated through correlation analysis of independent online databases (Fig. 5F and Supplementary Fig. 6E). Additionally, Western blot analysis demonstrated that TMED10-OE downregulated the levels of full-length and transcriptionally active nSREBP2 and SREBP1 forms of SREBP2 and SREBP1 (Supplementary Fig. 6F).

Next, to explore TMED10’s impact on EZH2-regulated cholesterol metabolism, we overexpressed TMED10 in EZH2-OE HEY and SKOV3 cells. Western blot confirmed significant endogenous overexpression of TMED10 in these cells (Fig. 5G). Intracellular cholesterol analysis revealed that TMED10-OE reversed EZH2’s promotion of cholesterol metabolism, reducing total and free cholesterol levels in HEY and SKOV3 cells (Fig. 5H). Moreover, TMED10-OE markedly decreased the secretion of APOB and APOA2 from HEY and SKOV3 cells (Fig. 5I). To validate TMED10’s role in EZH2-driven cholesterol metabolism and secretion, we knocked down TMED10 in EZH2-overexpressing HEY and SKOV3 cells (Supplementary Fig. 6G). However, consistent with TMED10’s standalone effects on ovarian cancer cells, TMED10 knockdown triggered noticeable apoptosis in HEY and SKOV3 cells. Western blot revealed significantly increased expression levels of apoptosis-related proteins Cleaved-PARP and Cleaved-Caspase-3 (Fig. 5J). Consequently, further investigation into TMED10 knockdown’s impact on cholesterol metabolism and secretion was precluded. In summary, our findings reveal a complex interplay between TMED10 and EZH2 in regulating ovarian cancer cell proliferation and cholesterol metabolism, offering novel insights for potential therapeutic strategies.

### EZH2 coordinates metabolic reprogramming through malonyl-CoA diversion and protein malonylation

**Fig. 6:**
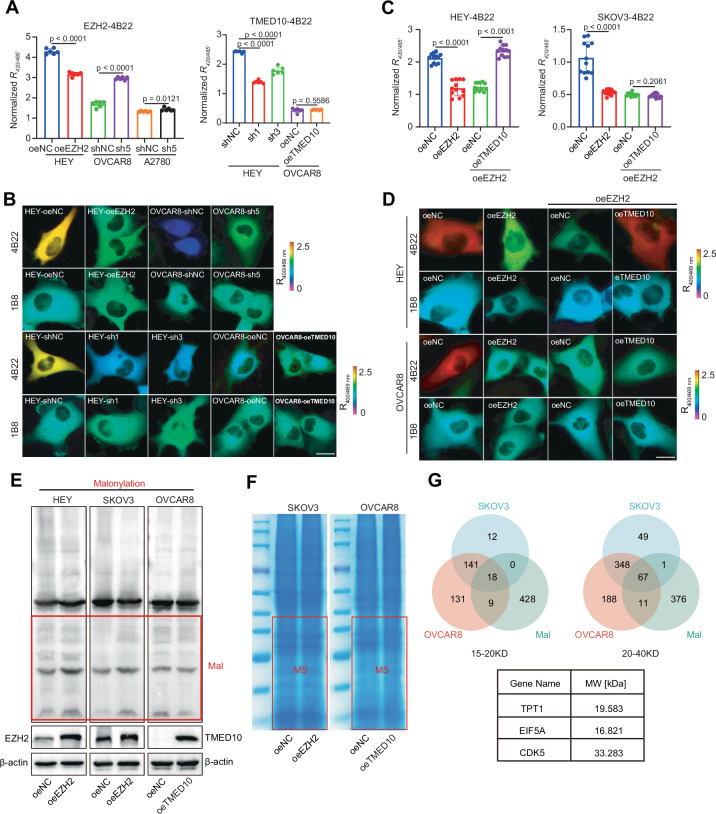
EZH2 coordinates metabolic reprogramming through malonyl-CoA diversion and protein malonylation. **A** Fluorometric analysis of Mal-CoA probe activity in EZH2-OE HEY and EZH2-KD OVCAR8 and A2780 cells using a microplate reader (left). Mal-CoA probe activity in TMED10-OE OVCAR8 and TMED10-KD HEY cells using a microplate reader (right). *n* = 6 cell cultures from three independent experiments. **B** Live-cell imaging of malonyl-CoA (Mal-CoA) probe metabolism in EZH2-OE HEY and EZH2-KD OVCAR8 cells (top), and in TMED10-KD HEY and TMED10-OE OVCAR8 cells (bottom). Scale bar, 50 μm. Data are representative of three independent biological replicates. **C** Fluorometric analysis of Mal-coA probe activity in EZH2-OE HEY and EZH2-KD OVCAR8 cells using a microplate reader. Data represent three independent biological replicates. **C** Fluorometric quantification of Mal-CoA level restoration in EZH2-OE HEY and SKOV3 cells with TMED10 overexpression. *n* = 12 cell cultures from three independent experiments. **D** Live-cell imaging demonstrating Mal-coA level restoration in EZH2-OE HEY and SKOV3 cells with TMED10 overexpression. Scale bar, 50 μm. Data are representative of three independent biological replicates. **E** Immunoblotting analysis of protein malonylation levels in EZH2-OE HEY and SKOV3 cells, as well as TMED10-OE OVCAR8 cells. Representative immunoblots from at least two independent experiments are shown. **F** Coomassie staining of SDS-PAGE gels for EZH2-OE SKOV3 and TMED10-OE OVCAR8 cells. Data are representative of two independent experiments. **G** Venn diagram showing overlapping proteins from mass spectrometry results of SKOV3 and OVCA8 compared to the Malonyl-CoA data (Mal-DB). The representative genes were TPTA, EIF5A, and CDK5. For (**A**, **C**), Data represent the mean ± s.d., and statistical analyses were performed using two-tailed unpaired t-tests.

Our research demonstrates that EZH2 promotes cholesterol biosynthesis by activating cholesterol metabolism-related genes. We employed fluorescent biosensors to monitor real-time metabolic dynamics in live ovarian cancer cells. EZH2-OE HEY cells exhibited significant reductions in malonyl-CoA levels versus controls, while EZH2-KD OVCAR8 and A2780 cells showed noticeable increases (Fig. 6A, B). Concurrently, TMED10-KD in HEY cells significantly reduced malonyl-CoA levels, whereas TMED10-OE in OVCAR8 cells exerted no significant effect on malonyl-CoA concentrations (Fig. 6A, B).

Further metabolic profiling demonstrated that EZH2 overexpression significantly reduced malonyl-CoA levels; subsequent TMED10-OE in these EZH2-high models restored malonyl-CoA concentrations in HEY and SKOV3 cells (Fig. 6C, D). These data demonstrate EZH2-mediated metabolic rewiring that promotes cholesterol biosynthesis via citrate shunting toward sterol production and suppresses fatty acid synthesis through malonyl-CoA depletion.

Given EZH2’s regulation of malonyl-CoA, we explored its overexpression-induced reduction of malonyl-CoA. Western blotting revealed a significant increase in protein malonylation in EZH2-OE HEY and SKOV3 cells but a decrease in TMED10-OE OVCAR8 cells (Fig. 6E). LC-MS/MS analysis of differentially malonylated bands (Fig. 6F) identified 86 proteins (FDR < 0.01, MW 15–20 and 20–40 kDa), with TPT1, EIF5A, and CDK5 prioritized based on cancer relevance (Fig. 6G). However, verification of modification, sites, and mechanisms requires more research. This study establishes EZH2 as a metabolic gatekeeper that redirects acetyl-CoA flux toward cholesterol biosynthesis through malonyl-CoA sequestration via protein malonylation, providing a novel mechanism for sterol-driven oncogenesis.

### The therapeutic synergy between pravastatin and EZH2i in ovarian cancer

**Fig. 7:**
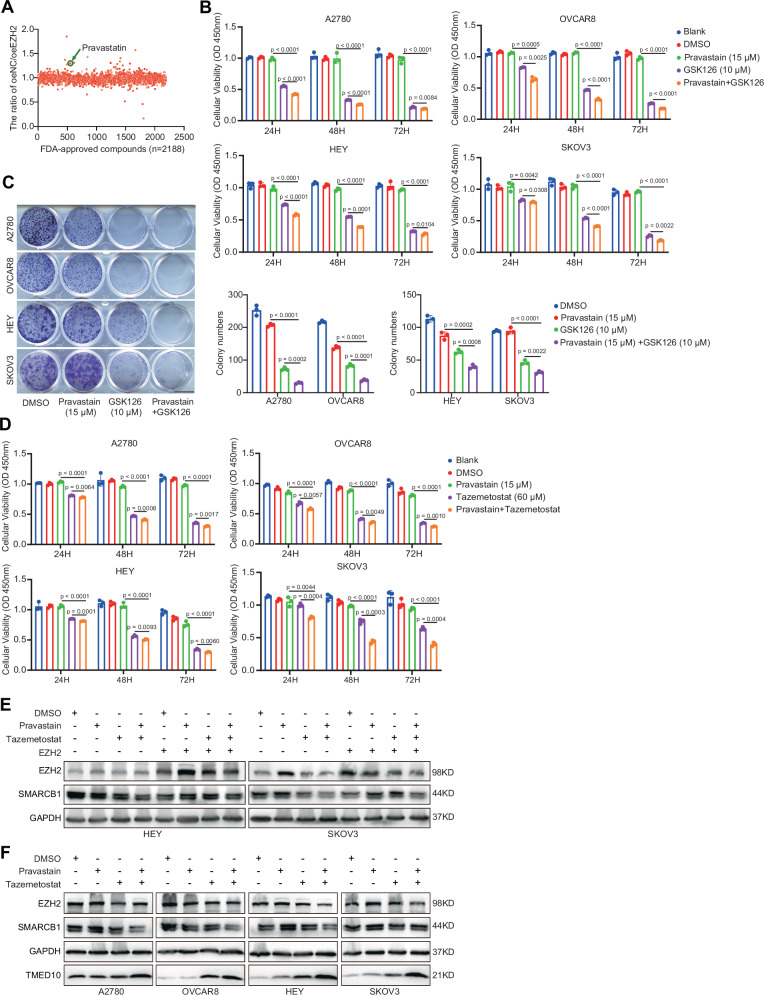
The therapeutic synergy between pravastatin and EZH2i in ovarian cancer. **A** High-throughput drug screening of 2188 FDA-approved compounds on cell viability in EZH2-overexpressing (oeEZH2) HEY and control (oeNC) cells. **B** Cell viability in low EZH2-expressing HEY and SKOV3 cells (bottom), and high EZH2-expressing A2780 and OVCAR8 cells (top), treated with Pravastatin (15 μM), GSK126 (10 μM), or their combination for 24 h, 48 h, or 72 h; n = 3 biological replicates, assessed as two experiments. **C** Clonogenic capacity of OVCAR8, A2780, HEY, and SKOV3 cells treated with Pravastatin (15 μM), GSK126 (10 μM), or their combination for 7 days; scale bar, 200 μm; add medicine the next day, data from three independent cultures. **D** Cell viability in low EZH2-expressing HEY and SKOV3 cells (bottom), and high EZH2-expressing A2780 and OVCAR8 cells (top) treated with Pravastatin (15 μM), Tazemetostat (60 μM), or their combination for 24 h, 48 h, or 72 h; *n* = 3 biological replicates, assessed as two experiments. **E** Immunoblotting analysis of EZH2 and SMARCB1 in HEY and SKOV3-oeEZH2 compared to oeNC cells treated with Pravastatin (15 μM), Tazemetostat (60 μM), or their combination for 72 h. Images are representative of three independent experiments. **F** Immunoblotting analysis of EZH2, TMED10, and SMARCB1 in A2780, OVCAR8, HEY and SKOV3 cells treated with Pravastatin (15 μM), Tazemetostat (60 μM), or their combination for 72 h. Images are representative of two independent experiments. For (**B**–**D**), Data represent the mean ± s.d., and statistical analyses were performed using two-tailed unpaired t-tests.

Because of the crucial role of cholesterol biosynthesis in tumor progression, we explored the therapeutic efficacy of the combination of EZH2 inhibitors (EZH2i) and cholesterol-lowering agents in ovarian cancer. High-throughput drug screen identifies HMG-CoA inhibition as a vulnerability in EZH2-high models. Screening of 2,188 FDA-approved compounds revealed heightened sensitivity of EZH2-overexpressing (OE) HEY cells to pravastatin, an HMG-CoA reductase inhibitor (Fig. 7A). We then assessed the cytotoxic effects of pravastatin, EZH2 inhibitors (GSK126 and Tazemetostat), and their combinations on ovarian cancer cells using CCK-8 and Colony formation assays. Results showed that in cells with low EZH2 expression (HEY, SKOV3, OV90), pravastatin alone had minimal effect, while GSK126 alone significantly inhibited cell viability. Notably, the combination of pravastatin and GSK126 demonstrated a more potent cytotoxic effect than either agent alone (Fig. 7B, C and Supplementary Fig. 7A, B). Similar results were observed in cells with high EZH2 expression (A2780, OVCAR8, ES-2). Moreover, the combination therapy showed greater efficacy in cells with higher EZH2 expression (Fig. 7B, C and Supplementary Fig. 7A, B). Tazemetostat, another EZH2 inhibitor approved by the FDA for epithelioid sarcoma and follicular lymphoma, was also studied. In cells with low EZH2 expression, Tazemetostat alone inhibited cell viability, and its combination with pravastatin further enhanced this inhibition. The same trend was observed in cells with high EZH2 expression (Fig. 7D and Supplementary Fig. 7C). Meanwhile, the combination therapy showed greater efficacy in cells with higher EZH2 expression.

Tazemetostat exhibited favorable tolerability and clinical activity in patients with advanced epithelioid sarcoma characterized by INI1/SMARCB1 deficiency. This agent holds promise for improving treatment outcomes in advanced epithelioid sarcoma [27]. Therefore, we sought to investigate the expression status of SMARCB1 in ovarian carcinomas and whether pravastatin enhances the anti-tumor efficacy of tazemetostat. We first assessed SMARCB1 expression across ovarian carcinoma cell lines, revealing low expression in OVCAR8, A2780, SKOV3, and OV90 cells compared to elevated levels in ES-2. Subsequent validation in EZH2-overexpressing HEY and SKOV3 models demonstrated marginal suppression of SMARCB1 expression in HEY cells but no significant alteration in SKOV3 cells (Supplementary Fig. 7D, E). In EZH2-OE HEY and SKOV3 cells treated with pravastatin and tazemetostat, western blot revealed that tazemetostat monotherapy suppressed both EZH2 and SMARCB1 protein abundance, whereas combination treatment demonstrated enhanced suppression (Fig. 7E). Finally, we evaluated the inhibitory effects of pravastatin and tazemetostat across a panel of ovarian carcinoma cell lines. The results demonstrated significantly enhanced suppression of EZH2 and SMARCB1 protein levels with combination therapy versus tazemetostat monotherapy. Notably, this reduction was more pronounced for SMARCB1 in A2780, OVCAR8, and ES-2 models exhibiting relatively higher baseline SMARCB1 expression. Concomitantly, exogenous suppression of EZH2 expression substantially elevated TMED10 protein abundance (Fig. 7F and Supplementary Fig. 7F).

Given the significant effects of the combination of cholesterol and EZH2 inhibitors, we investigated whether pravastatin could also enhance the sensitivity of PARP inhibitors, using Niraparib as a representative. However, Co-treatment with pravastatin and the PARP inhibitor Niraparib failed to enhance cytotoxicity in either EZH2-high or low models (Supplementary Fig. 7G), confirming the specificity of EZH2-cholesterol metabolic crosstalk. This study provides preclinical evidence for co-targeting EZH2 and cholesterol metabolism as a precision strategy in ovarian cancer, particularly for tumors with elevated EZH2 expression.

### Napabucasin emerges as a potent therapeutic agent for EZH2-high ovarian carcinoma

**Fig. 8:**
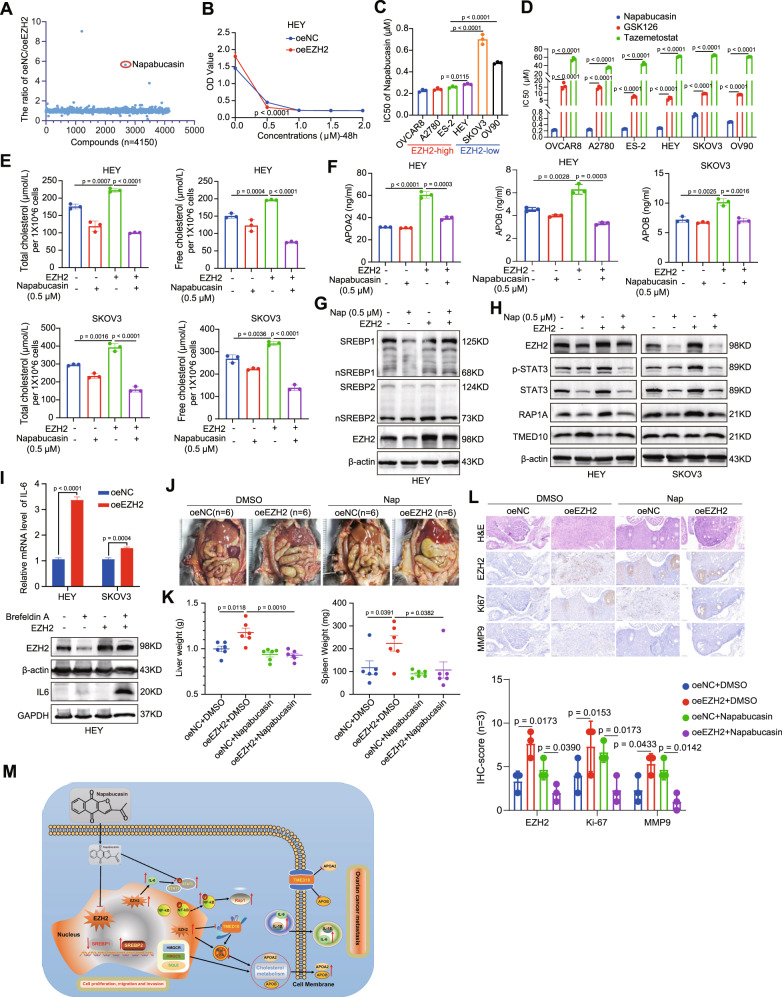
Napabucasin Emerges as a Potent Therapeutic Agent for EZH2-High Ovarian Carcinoma. **A** High-throughput drug screening of 4510 compounds (1962 kinase inhibitors and 2188 FDA-approved agents) on cell viability in EZH2-overexpressing (oeEZH2) HEY and control (oeNC) cells. **B** Napabucasin sensitivity at varying concentrations (48 h treatment) in HEY oeEZH2 and oeNC cells. Data represent three independent biological replicates. **C** IC50 values of Napabucasin against cell viability of EZH2-high (OVCAR8, A2780, ES-2) and EZH2-low (HEY, SKOV3, OV90) cell groups; *n* = 3 biological replicates, assessed as three experiments. **D** IC50 values of Napabucasin against cell viability of a panel of OV cell lines; *n* = 3 biological replicates, assessed as three experiments. **E** Intracellular total cholesterol and free cholesterol levels in EZH2-OE HEY (top) and SKOV3 (bottom) cells treated with Napabucasin (0.5 μM) for 48 h. Data are from three independent biological replicates. **F** APOB and APOA2 secretion levels in EZH2-OE HEY, and APOB secretion in EZH2-OE SKOV3 cells treated with Napabucasin (0.5 μM) for 48 h. Data are derived from three independent biological replicates. **G** Immunoblotting analysis of SREBP1 (full-length), nSREBP1 (nuclear SREBP1), SREBP2 (full-length), nSREBP2 (nuclear SREBP2), and EZH2 protein levels in EZH2-OE HEY cells post 48 h Napabucasin treatment. Images are representative of two independent experiments. **H** Immunoblotting analysis of EZH2, STAT3, p-STAT3, TMED10, and Rap1A protein levels in EZH2-OE HEY and SKOV3 cells following 48 h Napabucasin treatment. Representative immunoblots from at least two independent experiments are shown. **I** RT-qPCR analysis of IL-6 mRNA levels in EZH2-OE HEY and SKOV3 cells (top), results from three independent experiments; Immunoblotting analysis of EZH2 and IL-6 protein levels in HEY-oeEZH2 compared to oeNC cells following 12 h brefeldin A treatment (1:1000) (bottom). Representative immunoblots from two independent experiments are shown. **J** Representative images of ovarian tumor metastasis in ID8 oeEZH2 and oeNC mice treated with DMSO or Napabucasin (*n* = 6 mice per group; single experiment). **K** Liver and spleen weights of ID8 oeEZH2 and oeNC mice after treatment (*n* = 6 mice per group; single experiment). **L** H&E staining and immunohistochemical (IHC) analysis of EZH2, Ki-67, and MMP9 in tumor tissues from treated mice (*n* = 3 independent tumors per group). Scale bar, 50 μm. **M** Summary diagram of the full text. For (**C**–**F**, **I**, **K**, **L**) Data represent the mean ± s.d., and statistical analyses were performed using two-tailed unpaired t-tests.

PARP inhibitors have transformed the therapeutic landscape for ovarian cancer patients with BRCA mutations. However, analysis of cBioPortal data revealed significantly higher EZH2 expression in BRCA wild-type versus BRCA-mutated ovarian cancers, while TMED10 expression was statistically independent of BRCA status (Supplementary Fig. 8A). Critically, our work has validated EZH2 as a pivotal oncogenic target in ovarian cancer metastasis. Consequently, identifying therapeutic agents against this target represents a strategic imperative for ovarian cancer management. We conducted high-throughput drug screening across 1962 kinase inhibitors and 2188 FDA-approved compounds using isogenic HEY-oeEZH2 (overexpression) and -oeNC (empty vector control) cells. Napabucasin demonstrated exceptional selectivity against EZH2-OE cells (Fig. 8A). Next, CCK-8 assays confirmed Napabucasin’s enhanced potency in cells with high EZH2 expression, showing significant differential sensitivity at 0.5 μM (Fig. 8B and Supplementary Fig. 8B). Across six ovarian cancer lines stratified by baseline EZH2 expression (EZH2-high: A2780, OVCAR8, ES-2; EZH2-low: HEY, SKOV3, OV90), Napabucasin exhibited lower IC50 values in the EZH2-high group compared to EZH2-low groups at 48 h, indicating greater sensitivity of Napabucasin to cells with high EZH2 expression (Fig. 8C). To explore whether Napabucasin is more effective than traditional EZH2 inhibitors against ovarian cancer cells, we compared its inhibitory effects with those of the well-known EZH2 inhibitors GSK126 and Tazemetostat. Napabucasin demonstrated significantly lower IC50 values than GSK126 and Tazemetostat at 48 h across all models (Fig. 8D). We also found Napabucasin to outperform Niraparib (Supplementary Fig. 8C).

We investigated Napabucasin’s impact on EZH2-mediated cholesterol dysregulation. Analysis of intracellular cholesterol showed that Napabucasin markedly inhibited total and free cholesterol levels in EZH2-OE HEY and SKOV3 cells (Fig. 8E). ELISA revealed that Napabucasin significantly reduced APOB and APOA2 secretion in EZH2-OE HEY cells compared to controls. Similar suppression was also observed in SKOV3 EZH2-OE models (Fig. 8F). Furthermore, western blot demonstrated that Napabucasin downregulated SREBP2 expression while upregulating SREBP1 protein levels in EZH2-OE cells (Fig. 8G).

Notably, Napabucasin is a promising STAT3 inhibitor currently under clinical trials [28]. We have shown that EZH2-OE activated STAT3 signaling, resulting in increased p-STAT3 (Tyr705) compared to controls in HEY and SKOV3 cells (Supplementary Fig. 8D). Moreover, Napabucasin more effectively suppressed the protein levels of p-STAT3, EZH2, and downstream Rap1A while upregulating TMED10 protein levels in EZH2-OE cells (Fig. 8H). These findings further underscore Napabucasin’s enhanced sensitivity to cells with elevated EZH2 expression. To elucidate STAT3 activation mechanisms, we performed multiplex cytokine analysis of conditioned media from EZH2-OE HEY cells versus controls. This revealed significantly elevated IL-6 secretion in EZH2-OE cultures (Supplementary Fig. 8E). Consistently, correlation analysis of ovarian carcinoma specimens demonstrated a strong positive association between EZH2 and IL6 transcript levels (Supplementary Fig. 8E). Subsequent RT-qPCR and western blotting validation confirmed IL6 upregulation in EZH2-OE HEY and SKOV3 cells (Fig. 8I). Furthermore, ELISA analysis revealed that overexpression of EZH2 significantly increased, while knockdown of EZH2 markedly decreased, IL-6 secretion (Supplementary Fig. 8F). Finally, we used another STAT3 inhibitor-C188-9, to validate the finding that EZH2 overexpression significantly upregulated the level of p-STAT3. Notably, C188-9 exhibited superior suppression efficacy against p-STAT3 in EZH2-high cells. TMED10 expression patterns remained consistent with prior observations (Supplementary Fig. 8G). This research positions EZH2 activation of the IL-6-STAT3 axis and Napabucasin as a first-in-class agent targeting the EZH2-STAT3-metabolic nexus in ovarian cancer, providing a mechanistic rationale for its prioritized clinical development in biomarker-selected populations.

To validate Napabucasin’s therapeutic efficacy, we established an EZH2-OE ID8 murine ovarian cancer model (Supplementary Fig. 8H). In the ovarian orthotopic tumor model, compared to the oeNC+DMSO group, the oeEZH2+DMSO group exhibited a more significant increase in mouse weight, while the oeNC+Napabucasin and oeEZH2+Napabucasin groups experienced slower weight gain, with the latter being the slowest (Supplementary Fig. 8I). Concurrently, compared to oeNC+DMSO controls, oeEZH2+DMSO mice exhibited extensive intra-abdominal metastasis. Strikingly, Napabucasin treatment induced near-complete resolution of metastases in oeEZH2+Napabucasin mice versus oeNC+Napabucasin counterparts (Fig. 8J). Moreover, the oeEZH2+DMSO group displayed enlarged liver and spleen with increased weights, which were reduced and normalized in Napabucasin-treated mice (Fig. 8K). Finally, H&E and immunohistochemical analyses indicated that EZH2, MMP9, and Ki-67 staining intensities were more substantial in the oeEZH2+DMSO group but significantly weaker in the oeEZH2+Napabucasin group (Fig. 8L). This preclinical validation positions Napabucasin as a promising therapeutic for EZH2-driven ovarian cancer, addressing both primary tumor growth and lethal metastatic dissemination.

## Discussion

Our study confirms that EZH2 expression is significantly elevated in both primary ovarian cancer and metastatic lesions, and its high expression correlates with poor prognosis in ovarian cancer patients. However, the role of EZH2 in ovarian cancer metastasis and its regulation of cholesterol metabolism remain largely unexplored. This study demonstrates that EZH2-mediated cholesterol metabolism reprogramming is crucial in ovarian cancer progression. Moreover, it introduces Napabucasin, a small-molecule STAT3 inhibitor, which exhibits enhanced anti-tumor activity against ovarian cancers with high EZH2 expression, offering a promising therapeutic strategy for ovarian cancer treatment (Fig. 8M).

Our results show that, in the BRCA‑WT tumor cohort, EZH2 exhibited only a marginal trend for overall survival and no association with progression‑free survival. In univariate Cox regression analysis, EZH2 expression was not significantly associated with overall survival nor with progression‑free survival, and the EZH2×BRCA status interaction term was not significant for either overall survival or progression‑free survival. These analyses indicate that, although EZH2 is preferentially overexpressed in BRCA‑mutant tumors, our data do not provide strong evidence that EZH2 expression acts as an independent prognostic factor exclusively within the BRCA‑mutant subgroup. Furthermore, Hallmark gene set enrichment analysis (GSEA) revealed that EZH2‑high BRCA‑WT tumors are enriched in proliferation‑ and cell cycle‑related programs, including E2F targets, G2M checkpoint, MYC targets, mitotic spindle, DNA repair, and mTORC1 signaling. Thus, while EZH2 expression did not emerge as a clear independent prognostic factor in this cohort, it consistently marks a biologically distinct subset of BRCA‑WT tumors characterized by activation of proliferative pathways.

In this study, we found that EZH2 exerts its pro‑tumorigenic function by regulating the NF‑κB‑Rap1A axis. However, we did not observe direct binding of EZH2 to the NFKB1, RELA, or RAP1A genes, and alterations in EZH2 expression affected H3K27me3 levels, suggesting that EZH2 may not regulate the expression of these genes by directly binding to their promoters. This is consistent with several reports. For instance, it has been shown that EZH2 can indirectly activate the NF‑κB pathway through its methyltransferase activity by repressing the expression of the negative regulator TNFAIP3/A20 [29]. Another study in nasopharyngeal carcinoma cells demonstrated that EZH2 directly represses IKKα transcription by catalyzing H3K27me3 [30]. Furthermore, evidence indicates that EZH2 may indirectly activate RAP1A by suppressing the negative regulator Rap1GAP [31].

The other important downstream target of EZH2 is cholesterol metabolism. Apolipoprotein A-II (APOA2), a key component of high-density lipoprotein (HDL), regulates cholesterol transport and has been linked to cancer metastasis [32]. However, studies on breast cancer have shown conflicting results [33, 34]. We found that EZH2 overexpression in ovarian cancer cells significantly increased APOA2 expression, and APOA2 knockdown reverses EZH2-driven malignant proliferation and metastasis, highlighting its pro-tumorigenic role in ovarian cancer. Beyond APOA2, we identify apolipoprotein B (APOB), the primary low-density lipoprotein (LDL) protein component [35], as a functionally analogous driver of ovarian cancer progression. Elevated LDL cholesterol levels are positively correlated with breast tumor size, proliferation, and reduced cell adhesion, while also enhancing migration [36, 37]. These findings collectively underscore the pivotal roles of APOA2 and APOB in ovarian cancer progression. Key transcription factors tightly regulate cholesterol homeostasis, including sterol regulatory element-binding protein-2 (SREBP2) and liver X receptors (LXRs). Synthesized in the endoplasmic reticulum, SREBP2 is processed in the Golgi apparatus to its nuclear form (nSREBP2), which dimerizes and translocates to the nucleus to activate target gene transcription, such as HMGCR and SQLE [38]. Increased cholesterol biosynthesis is a hallmark of many cancers [16], as tumor cells reprogram cholesterol metabolism to promote cancer progression by regulating cell proliferation, migration, and invasion [16, 39]. Consistent with these observations, our study reveals that EZH2 overexpression upregulates SREBP2, leading to the transcriptional activation of downstream genes and aberrant cholesterol metabolism in ovarian cancer. Additionally, our study reveals that EZH2 also activates genes related to cholesterol esterification, efflux, and oxidation, further highlighting its role in cholesterol metabolism reprogramming in ovarian cancer.

In the process of fatty acid synthesis, malonyl-CoA combines with fatty acid synthase (FASN), ultimately forming palmitic acid [40]. Interestingly, our study revealed that EZH2 overexpression significantly suppressed the key gene SREBP1 in fatty acid synthesis and reduced malonyl-CoA levels. Consistently, EZH2, which promotes cholesterol synthesis, did not substantially activate p-AMPK levels to activate the AMPK pathway, which primarily inhibits anabolism and promotes catabolism [41]. Moreover, AMPK can block cholesterol biosynthesis by phosphorylating HMGCR [42]. Given cholesterol metabolism’s significant and diverse role in cancer progression, impeding active cholesterol metabolism has emerged as a viable anti-tumor strategy [43, 44].

Statins (HMGCR inhibitors) are the most extensively studied cholesterol metabolism-targeted drugs in cancer patients [45]. In recent years, a large body of epidemiological evidence has demonstrated that statin use significantly improves survival outcomes in ovarian cancer patients [46]. reducing all-cause mortality by 26% [47]. Furthermore, post-diagnostic statin use has been associated with a 10–25% increase in overall survival among patients with gynecologic malignancies [48]. In our study, we confirmed that the combination of pravastatin with the EZH2 inhibitors GSK126 or Tazemetostat exerts a robust synergistic effect against ovarian cancer cells. Tazemetostat holds promise for improving therapeutic outcomes in patients with advanced-stage epithelioid sarcoma characterized by SMARCB1 deficiency [27]. Concurrently, preclinical evidence identifies the IL-6/JAK/STAT3 signaling axis as a therapeutic vulnerability in SMARCB1-deficient bladder carcinomas [49]. Furthermore, our study revealed that the combination of pravastatin and tazemetostat led to a more pronounced reduction in the protein expression levels of both EZH2 and SMARCB1. This effect was notably more evident in cells exhibiting high SMARCB1 expression in ovarian cancer. However, Pravastatin showed no synergistic effect when used with the PARP inhibitor Niraparib, and the specific causes and mechanisms require further in-depth study.

We found that EZH2 negatively regulates TMED10 expression, and overexpressing TMED10 in EZH2-OE cells can reverse the effects of EZH2 on cholesterol metabolism and lipoprotein secretion, while also increasing malonyl-CoA levels. And, EZH2-OE-including APOB, HMGCS, DHCR7, and GALNT2 (which modulates lipid metabolism via O-glycosylation)-were conversely downregulated following TMED10 overexpression. TMED10 orchestrates non-classical protein secretion through the TMED10-channeled unconventional protein secretion (THU) pathway, facilitating the transport of cargo lacking signal peptides into the ER-Golgi intermediate compartment (ERGIC) [15]. Cholesterol is synthesized in the ER and transported to the Golgi via vesicles or OSBP [50, 51]. Once in the Golgi, cholesterol is incorporated into membrane carriers and exits to the plasma membrane [52]. TMED10, as a key organizer of ER-Golgi MCSs, is responsible for the specific transfer of cholesterol and ceramide between these two organelles [53, 54]. Studies have shown that TMED10 influences lipid flux through the secretory pathway, and its silencing alters intracellular cholesterol distribution. In HeLa cells, TMED10 silencing increases cholesteryl esters and upregulates genes involved in cholesterol biosynthesis or absorption [53]. This raises cholesterol precursors, such as malonyl-CoA, which negatively regulate cholesterol metabolism genes, leading to decreased SREBP2 expression. NFAT signaling regulates TMED10 expression via functional NFAT response elements in its promoter [55]. EZH2 inhibitors can upregulate the calcium-calcineurin-NFAT pathway [56]. consistent with our finding that EZH2 upregulation suppresses TMED10 expression, providing a mechanistic link between EZH2 and TMED10 suppression. Additionally, we observed altered TMED10 protein localization in EZH2-OE cells, suggesting that EZH2 may post-translationally modify TMED10, affecting its structure, activity, or protein interactions. These areas will be a focus for our future research.

Given the frequent overexpression of EZH2 in ovarian carcinomas and its established role in driving disease progression, particularly in BRCA wild-type tumors where EZH2 levels are significantly elevated and associated with PARP inhibitor insensitivity—defining novel EZH2 targets for combination therapy represents a critical imperative for improving clinical outcomes in this patient population. To address this need, we performed high-throughput drug screening using EZH2-overexpressing cells and found Napabucasin to be more effective against cells with high EZH2 expression. Notably, Napabucasin demonstrated greater sensitivity than classical EZH2 inhibitors, including GSK126 and Tazemetostat. Napabucasin is a cancer stemness inhibitor that targets STAT3-driven gene transcription [28, 57]. Preclinical studies and early-phase clinical trials have demonstrated promising anti-tumor activity of napabucasin in multiple malignancies, including colorectal, gastric, and pancreatic cancers. However, most subsequent Phase III trials failed to meet their primary endpoints [58–60]. Notably, exploratory analyses suggest a survival benefit of napabucasin in pSTAT3-positive subgroups [59]. highlighting the importance of biomarker-driven patient selection. Meanwhile, preclinical evidence indicates that combination of the EZH2-specific inhibitor DZNep with siSTAT3 significantly increases apoptosis in gastric cancer cells [61]. STAT3 plays a critical role in all stages of cancer metastasis, including invasion and migration [62]. Notably, pro-inflammatory cytokine IL-6 is linked to human tumor development [63]. Epithelial cells expressing receptors for IL-6 can directly activate STAT3 signaling, providing growth and survival signals for early tumor cells [64]. Our study further demonstrates that EZH2 overexpression upregulates IL6 expression and secretion while significantly elevating p-STAT3 levels. Importantly, Napabucasin treatment not only suppressed p-STAT3 expression but also reduced EZH2 and Rap1A protein levels, particularly in cells overexpressing EZH2. This highlights Napabucasin’s potential in treating ovarian cancers with high EZH2 expression. Furthermore, Napabucasin significantly inhibited cholesterol metabolism and lipoprotein secretion in ovarian cancer cells, as well as SREBP2 expression. In vivo experiments further demonstrated Napabucasin’s ability to significantly inhibit ovarian cancer metastasis. Our findings position Napabucasin as a promising therapeutic candidate for EZH2-high ovarian cancer, offering new hope for patients facing this aggressive malignancy. By targeting both epigenetic and metabolic vulnerabilities, Napabucasin exemplifies the potential of multi-pathway inhibition to overcome therapeutic resistance and enhance clinical outcomes.

## Methods

All studies complied with relevant ethical guidelines and were approved by the Institutional Ethics Committee of Tongji University Affiliated Obstetrics and Gynecology Hospital (#KS22335). Mice were housed under controlled environmental conditions (temperature: 25 °C; relative humidity: 60–70%; 12-h light/dark cycle). All animal experiments were performed under protocols approved by the Tongji University Institutional Animal Care and Use Committee.

### Clinical samples

Clinical specimens for this study were collected from patients undergoing laparoscopic surgery for benign ovarian cysts or ovarian cancer at Tongji University Affiliated Obstetrics and Gynecology Hospital. The study involved two groups of patients. The first group included 25 patients with benign ovarian cysts and 92 patients with ovarian cancer (24 of whom had metastatic ovarian cancer) who underwent surgery from January 2017. Fresh tissue samples were gathered during surgery and cryopreserved. Two pathology experts confirmed the tissue pathology types for all patients. The second group comprised patients who underwent laparoscopic surgery or surgery for ovarian cancer between February 2022 and December 2023, from which 28 normal tissue samples (normal fallopian tube and benign ovarian tissue) and 56 ovarian cancer tissue samples were collected for RNA-seq, RT-qPCR, and Western blotting experiments. All diagnoses were pathologically validated after surgery. Sample collection was approved by the hospital’s ethics committee, and informed consent was obtained from all patients. Clinicopathological data are detailed in Supplementary Table 2.

### Cell culture

All cell lines were maintained at 37 °C with 5% CO₂ in humidified cell culture incubators (Thermo Fisher Scientific). The incubators were routinely calibrated and maintained according to the manufacturer’s specifications. Source and media information for all cells are provided in Supplementary Table 1. To ensure experimental integrity, cells were regularly screened for mycoplasma contamination using PCR-based assays, with no positive results detected throughout the study.

### Inhibitors and sources

The inhibitors used in this study- Napabucasin (HY-13919), GSK126 (HY-13470), Tazemetostat (HY-13803), Niraparib (HY-10619), BAY 11-7082 (HY-13453), and C188-9 (HY-112288)-were procured from MedChemExpress (MCE, China).

### Bioinformatics analysis

The data of cell lines were obtained from the Cancer Cell Line Encyclopedia (CCLE, https://portals.broadinstitute.org/ccle/). EZH2 and TMED10 expression in ovarian cancer was obtained from cBioPortal (http://www.cbioportal.org/). Correlation expression in all cancers or only ovarian cancer from the datasets in cBioPortal (id=pancan_pcawg_2020 and ID=Ovary/Fallopian_OC_2024) [65]. Survival analysis was conducted using the Kaplan–Meier Plotter database (http://kmplot.com/analysis/), stratifying ovarian cancer patients into EZH2/TMED10-low and EZH2/TMED10-high subgroups based on median expression thresholds to evaluate progression-free-survival (PFS) differences.

RNA-seq data processed via the STAR pipeline were downloaded from the TCGA data (https://portal.gdc.cancer.gov) (Project TCGA-OV; ovarian serous cystadenocarcinoma) and converted to TPM format. Samples were stratified into low-expression (bottom 50% quantile, *n* = 190) and high-expression (top 50% quantile, *n* = 191) groups based on EZH2 or TMED10 levels. Differential expression analysis was then performed using DESeq2 (v1.36.0) in R.

Expression data of 427 individuals with ovarian cancer (OV) were downloaded from the GDC (https://portal.gdc.cancer.gov/projects). Expression data and clinical data of 88 normal tissues were downloaded from GTEx (https://xenabrowser.net/datapages/). RNA-seq data processed via the STAR pipeline from the pan-cancer TCGA cohort (TCGA-ALL) were downloaded via the GDC. Matched and unmatched tumor-normal pairs were extracted. Statistically appropriate methods from the stats (v4.1.0) and car (v3.1-2) R packages were applied—proceeding only when test assumptions were met. Results were visualized using ggplot2 (v3.4.0). Detailed statistical data are presented in Supplementary Tables 3 and 4.

### Survival analyses

Clinical, BRCA1/2 mutation, and RNA-seq expression data were downloaded from the cBioPortal TCGA high-grade serous ovarian cancer cohort (hgsoc_tcga_gdc). BRCA status was harmonized as wild-type (WT) versus mutant (Mut). Within BRCA-WT tumors, Kaplan–Meier analyses compared patients with high versus low EZH2 expression using a top-versus-bottom quartile approach for the primary analysis, and significance was assessed by log-rank test. EZH2 was also analyzed as a continuous variable by z-score standardization, and Cox proportional hazards models were used to estimate hazard ratios per 1 standard deviation increase in expression. To test whether the prognostic association of EZH2 differed by BRCA status, interaction Cox models were fit in the full cohort using an EZH2 × BRCA status term, with age included as an adjusting covariate where applicable. All analyses were performed in R with the survival and survminer packages.

### Hallmark GSEA in BRCA-WT tumors

RNA-seq expression, BRCA1/2 mutation annotation, and clinical data were obtained from the cBioPortal TCGA high-grade serous ovarian cancer cohort (hgsoc_tcga_gdc). After merging data at the patient level and restricting to BRCA-wild-type (BRCA-WT) tumors, patients were ranked by EZH2 mRNA expression and divided into top- and bottom-quartile groups (101 tumors each). For transcriptome-wide comparison, one representative expression row per gene symbol was retained after collapsing duplicate rows by variance. Differential expression between EZH2-high and EZH2-low BRCA-WT tumors was performed using limma with the contrast High_vs_Low. For Hallmark GSEA, genes were ranked by the limma moderated t statistic, duplicate gene symbols were removed, and enrichment analysis was carried out using fgsea with the MSigDB Hallmark collection (collection = “H”) and 10,000 permutations. Results were ordered by adjusted *P* value and absolute normalized enrichment score (NES), and the top enriched pathways were visualized in dot plots in which x-axis position represents NES, point size represents gene-set size, and color represents −log10(adjusted *P* value). Detailed statistical data are presented in Supplementary Table 3.

### H&E and IHC

The first group of patients’ clinical tissue samples, made into a tissue microarray, were processed as follows: Melt the surface paraffin by baking the tissue microarray in a 60 °C constant-temperature oven for 2 h. Deparaffinize and hydrate it, then repair antigens per IHC antibody instructions. Add 3% hydrogen peroxide to inactivate endogenous peroxidase activity, then block the sections. Incubate with primary and secondary antibodies successively. Finally, carry out the DAB reaction, counterstain with hematoxylin, dehydrate, clear, and mount the sections before scanning.

After euthanasia, mouse tissues were fixed in 4% paraformaldehyde for 24 h, then dehydrated, cleared, paraffin-infiltrated, and embedded. The embedded blocks were sectioned into 4-micron slices for subsequent H&E staining and IHC analysis. All procedures were performed under sterile conditions. H&E staining was conducted first to examine tissue morphology, followed by IHC staining to detect target protein expression. The antibodies used are detailed in Supplementary Table 1.

Immunohistochemical (IHC) scoring was based on staining intensity and the percentage of positive cells. Staining intensity was scored as follows: 0, no staining or background‑like staining; 1, light yellow (above background); 2, yellowish‑brown (clearly above background); 3, dark brown (strong). The percentage of positive cells was scored as: 0, <5%; 1, 5–25%; 2, 26–50%; 3, 51–75%; 4, 76–100%. The final IHC score was calculated as the product of the intensity score and the percentage score, and was categorized into four levels: 0–3 (negative), 4–6 (weak), and 8–12 (strong). Detailed statistical data are presented in Supplementary Table 2.

### RT-qPCR

Total RNA was isolated from tissues and cells using RNAiso Plus (TaKaRa, 9109). Reverse transcription was subsequently performed on a PCR system (Applied Biosystems) to synthesize cDNA following the manufacturer’s instructions of ABclonal’s ABScript III RT Master Mix for qPCR with gDNA Remover (RK20429). Quantitative real-time PCR (RT-qPCR) analysis was then conducted using a real-time PCR detection system (Thermo Fisher) with ABclonal’s BrightCycle Blue Universal SYBR Green qPCR Mix with UDG (RK21220), strictly adhering to the recommended protocols. Primer sequences for all target genes analyzed in RT-qPCR experiments are detailed in Supplementary Table 1. For data normalization, β-actin or GAPDH was employed as a housekeeping gene throughout this study. Raw data for all RT-qPCR experiments in this study are shown in Supplementary Table 5.

### Western blotting

Cellular proteins were extracted using ice-cold RIPA lysis buffer (Beyotime, P0013B) supplemented with protease/phosphatase inhibitor cocktail (NCM Biotech, P002) and PMSF (Beyotime, ST507-10 ml). Lysates were sonicated and centrifuged at 14,000 × *g* for 10 min at 4 °C, and supernatants were collected for protein quantification using the TaKaRa BCA Protein Assay Kit (T9300A) following the manufacturer’s specifications. Protein samples were denatured at 100 °C using a metal bath for 10 min with 5X Omni-Easy™ Instant Loading Buffer (Epizyme, LT101L), then resolved through SDS-PAGE electrophoresis. Separated proteins were transferred to PVDF membranes (Millipore, IPVH00010) and blocked for 1 h with protein-free rapid blocking buffer (Epizyme, PS108P). Membranes underwent overnight incubation at 4 °C with primary antibodies. After washing with Tris-buffered saline containing 0.5% Tween 20, the membrane was incubated with horseradish peroxidase-conjugated secondary antibody at room temperature for 1 h. Protein signals were visualized using a chemiluminescence detection system (Tanon). Complete antibody specifications are detailed in Supplementary Table 1.

### RNA isolation, RNA-seq, and analysis

Total RNA was isolated from EZH2-oeNC and EZH2-OE HEY cells (cultured to ~80% confluence) using RNAiso Plus (TaKaRa, 9109). mRNA was enriched from total RNA via oligo-dT magnetic bead selection and fragmented enzymatically. Subsequent cDNA library preparation involved sequential cDNA synthesis, purification, end repair, poly-A tailing, adapter ligation, and PCR amplification. Paired-end sequencing (150 bp) was performed on the Illumina HiSeq2500 platform (Sequanta). Gene/transcript expression quantification was conducted using RSEM (v1.3.3) with default parameters. Raw counts underwent normalization via the TMM (trimmed mean of M-values) method and CPM (counts per million) transformation using edgeR (v3.28.1) in the R environment. Functional enrichment analysis was performed with clusterProfiler (v4.0), applying statistical thresholds of *p* < 0.01 for Gene Ontology (GO) terms and *p* < 0.1 for KEGG pathways. Significantly enriched terms were identified through hypergeometric testing with Benjamini-Hochberg correction. Detailed data is provided in Supplementary Tables 7 and 9.

### Plasmid construction, lentiviral transduction, and generation of stable cell lines

The PGMLV-CMV-Mcs-3xFlag-EZH2-mCherry-T2A-Puro and PGMLV-CMV-Mcs-3xFlag-TMED10-mCherry-T2A-Puro overexpression plasmids were commercially obtained from Genomeditech. shRNA constructs (shNC, shEZH2, and shTMED10) were acquired from Genechem. Dox-induced EZH2 knockdown lentivirus with pLent-TRE3G-ZsGreen-mir30-hPGK-rtTA-SV40-Puro vector was purchased from Shandong Weizen Biotechnology Co., LTD. All other plasmids were generated through experimental construction.

Linearized cloning vectors and inserts were prepared and subjected to agarose gel electrophoresis, followed by homologous recombination for overexpression plasmid construction. For knock-down plasmid construction, sequences and primers were designed and annealed. Vectors were then digested and run on agarose gels. Oligos were ligated to digested vectors using T4 DNA Ligase (NEB, M0202S). The mixtures were transformed, plated, and single colonies were picked. Finally, sequencing was performed, and bacterial cultures with correct sequences were frozen.

HEK293T cells were seeded in 10 cm dishes and transfected at ~60% confluence with 8 μg of target plasmid combined with packaging plasmids (2.67 μg pMD2.G + 5.3 μg psPAX2) using PEI MAX transfection reagent (Polysciences, 24765-100). Viral supernatants were harvested at 48 h and 72 h post-transfection, filtered through 0.45 μm membranes, and concentrated via ultracentrifugation.

For transduction, target cells in 6-well plates were exposed to 1 mL lentiviral supernatant supplemented with 1 μL Polybrene (Genechem, REVG0001). The selection was initiated 48 h later using either puromycin (Solarbio, P8230-25 mg) or blasticidin S hydrochloride (Solarbio, B9300-10mg). Transduction efficiency was validated through RT-qPCR and immunoblotting before functional assays. All cell lines were maintained within 10 passages throughout the experiments. Complete plasmid sequences are detailed in Supplementary Table 1.

### siRNA transfection

Target cells were seeded in 6-well plates 24 h before transfection to achieve 60–70% confluency. Transfection complexes were prepared by incubating 10 μL of siRNA with 5 μL Lipofectamine 3000 (Invitrogen, 2532085) in a serum-free medium for 15 min at room temperature. Complexes were gently dispersed across cell monolayers, followed by incubation under standard culture conditions. RNA was harvested 48 h post-transfection for knockdown validation via qRT-PCR analysis or subsequent functional assays. The siRNA sequence targeting APOB (siAPOB) is provided in Supplementary Table 1.

### Proteomic profiling

TMED10-oeNC and TMED10-OE OVCAR8 cells were cultured in a complete medium until they reached approximately 70% confluence. After three washes with a serum-free medium, the cells were maintained in serum-free conditions for 48 h to collect the conditioned medium. Cellular debris was removed through sequential centrifugation supplemented with a protease inhibitor cocktail. Concentrated proteins were obtained via ultrafiltration at 4 °C and 5000 × *g* using 10 kDa centrifugal devices (Millipore), followed by buffer exchange with 8 M urea. Protein concentrations were quantified using a BCA assay. Samples underwent liquid chromatography-tandem mass spectrometry (LC-MS/MS) analysis (PTM Biolabs Inc) with a Q Exactive HF-X system (Thermo Scientific). Raw data were processed using MaxQuant (v2.0.3.1) and the UniProt human database (release 2023_01), applying a 1% false discovery rate (FDR) threshold. Differentially expressed proteins (DEPs) were defined as a fold change >1.5 or <0.67 with *p* < 0.05 (Student’s t-test). Functional annotation was carried out in Perseus (v1.6.15.0) utilizing Gene Ontology (GO) and KEGG pathway databases, with enrichment significance determined through hypergeometric testing (*p* < 0.05, Benjamini-Hochberg correction). Detailed data are provided in Supplementary Table 7.

### Chip-seq

ChIP DNA degradation and contamination was monitored on agarose gels. DNA purity was checked using the NanoPhotometer® spectrophotometer (IMPLEN, CA, USA). DNA concentration was measured using Qubit® DNA Assay Kit in Qubit® 3.0 Flurometer (Cosmos Wisdom Technologies, CA, USA). The purified DNA was used for ChIP-seq library preparation. The library was constructed by Origene Corporation (Hangzhou, China). Subsequently, pair-end sequencing of sample was performed on Illumina platform (Illumina, CA, USA). Library quality was assessed on the Agilent Bioanalyzer 2100 system. Raw data of fastq format were firstly processed using fastp software. All the downstream analysis were based on the clean data with high quality. Reference genome and gene model annotation files were downloaded from genome website directly. Index of the reference genome was built using BWA (version 0.7.17) and clean reads were aligned to the reference genome using BWA mem (version 0.7.17). Different peak analysis was based on the fold enrichment of peaks of different experiments. A peak was determined as different peak when the odds ratio between two groups was more than 2. Using the same method, genes associated with different peaks were identified, and also do GO and KEGG enrichment analysis. Detailed data are provided in Supplementary Table 9.

### LC-MS/MS proteomics

After Western blot electrophoresis, the gel was stained with Coomassie Brilliant Blue. The target gel bands were excised and destained with an appropriate amount of silver staining destaining solution. The gel pieces were dehydrated with 200 μL of pure acetonitrile for 5 min and then air‑dried at room temperature for 10 min. Subsequently, 200 μL of protein reduction solution was added to fully cover the gel pieces, and the samples were incubated in a thermomixer at 1000 rpm and 50 °C for 30 min. The gel pieces were then dehydrated with 200 μL of pure acetonitrile in a thermomixer at 25 °C and 1000 rpm for 5 min. Trypsin was added to completely cover the gel pieces, followed by incubation in a thermomixer at 37 °C and 1000 rpm for 16 h (overnight). Then, 150 μL of peptide extraction buffer was added, and the samples were incubated in a thermomixer at 1000 rpm and 25 °C for 30 min. The digested solution was transferred to a new EP tube, and the extraction was repeated once. The FASP (filter-aided sample preparation) procedure was then performed, followed by desalting and lyophilization. The dried peptides were dissolved in 20 μL of 0.1% formic acid and subjected to LC‑MS/MS analysis. The raw mass spectrometry data (RAW format) were searched and analyzed using Proteome Discoverer software (version 1.4, Thermo Fisher Scientific). Detailed data are provided in Supplementary Table 5.

### Transwell assay

A Transwell assay was performed to evaluate cellular migration and invasion. For migration assays, the upper chamber (8.0 μm pore size, Corning, 3422) was used without Matrigel (Corning, 356234), while for invasion assays, 70 μL of Matrigel was added to the upper chamber. A mixture of 200 μL serum-free medium and cell suspension was placed in the upper chamber, and 600 μL medium with 10% FBS was added to the lower chamber. After 48 h of incubation, the Transwell chambers were removed. The remaining cells were fixed with 4% paraformaldehyde for 30 min at room temperature, washed with PBS, and then stained with crystal violet for 30 min. Migrated and invaded cells were observed and photographed under an inverted microscope (Olympus). The number of migrated and invaded cells was analyzed using ImageJ software. Detailed statistical data about the experimental results are provided in Supplementary Table 6.

### CCK-8 assay

Cells (1 × 10^3^) were seeded in quintuplicate wells of 96-well plates (100 μL cell suspension per well) with perimeter wells filled with 100 μL PBS to minimize evaporation. Following initial adherence (4–6 h at 37 °C, 5% CO₂), Day 0 baseline measurements were acquired by replacing the medium with 90 μL fresh medium plus 10 μL CCK-8 reagent (MCE, HY-K0301) under light-restricted conditions. Plates were incubated for 2 h with gentle agitation before measuring absorbance at 450 nm using a microplate reader. Subsequent measurements (Days 1–5) followed identical protocols with daily medium replacement. Detailed statistical data about the experimental results are provided in Supplementary Table 6.

### Clonogenic assay

Cells were seeded in 6-well plates at a density of 1000 cells/well in 2 mL complete medium and maintained at 37 °C with 5% CO₂ for 14 days to allow colony formation. Colonies were fixed with 4% paraformaldehyde (RT, 30 min), stained with 0.5% crystal violet (Sigma-Aldrich, V5265; RT, 30 min), and air-dried. Data represent mean ± SD from three independent experiments. Detailed statistical data about the experimental results are provided in Supplementary Table 6.

### Cholesterol quantification analysis

Cellular total cholesterol and free cholesterol levels were determined enzymatically using commercial assay kits (Applygen, Total Cholesterol Assay Kit, E1015; Free Cholesterol Assay Kit, E1016) following the manufacturer’s protocols. All measurements were performed in triplicate biological replicates. Detailed statistical data about the experimental results are provided in Supplementary Table 8.

### ELISA

An ELISA kit for human APOB (KE00158) and IL6 (KE00385) were purchased from Proteintech; a kit for human ApoA2 (E-EL-H6039) from Elabscience; and a Human IFN-gamma ELISA Kit (RK00015) from ABclonal. All assays were performed according to the kit instructions. Standard curves were generated through four-parameter logistic regression (SoftMax Pro v7.1), with analyte concentrations calculated via weighted least squares fitting. All data represent mean ± SD from three independent experimental replicates. Detailed statistical data about the experimental results are provided in Supplementary Table 8.

### Metabolic probe transfection and biosensing

The plasmid was transfected into 293T cells at a ratio of pMD2.G: pSPAX2: target plasmid = 1:3:4. Supernatants were collected at 48 and 72 h post-transfection, filtered through a 0.45 μm filter, and ultracentrifuged to collect viral particles. The virus was diluted and used to infect target cells in the presence of 8 μg/mL Polybrene. After 4–6 h, fresh medium replaced the old. Cells were selected with antibiotics 48 h post-infection, with the antibiotic concentration reduced later for maintenance.

For real-time metabolic monitoring, 1 × 10³ cells/well were seeded in black-walled 96-well plates (Corning, 3340). Cells were transfected with biosensor plasmids using Lipofectamine3000™. Following 36–48 h expression, cells were washed twice with HBSS (Gibco, 14025092) and imaged using an inverted fluorescence microscope (Olympus IX73) with dual excitation/emission settings: 420/532 nm (ratiometric mode) and 485/532 nm (intensity mode). Fluorescence quantification was conducted using a multimode microplate reader (MD, M5) at 37 °C with environmental control. Detailed statistical data about the experimental results are provided in Supplementary Table 8.

### Coomassie Brilliant Blue staining

Following SDS-PAGE electrophoresis, gels were rinsed in ultrapure water (Milli-Q, 18.2 MΩ cm). Staining was performed using Coomassie Brilliant Blue (Epizyme, PS111) at room temperature for 20 min. Destaining involved five sequential 20-mins wash in ultrapure water under continuous agitation (100 rpm), followed by overnight equilibration in fresh ultrapure water to enhance contrast. Image acquisition was performed using a digital imaging system (Azure Biosystems C600) under uniform white light illumination. Gel band intensities were quantified via Image Lab™ software (v6.1, Bio-Rad) using local background subtraction.

### Nuclear-cytoplasmic fractionation assay

Cells cultured in 10-cm dishes at ~60% confluence were treated with 3 μM BAY 11-7082 (NF-κB inhibitor; MCE, HY-13453) for 48 h under standard culture conditions (37 °C, 5% CO₂). Following manufacturer protocols, fractionation was performed using the Nuclear and Cytoplasmic Protein Extraction Kit (Beyotime, P0027).

### LPS stimulation in RAW264.7 cells

RAW264.7 murine macrophages were maintained in a specialized medium (Pricella, CM-0190) and seeded in 6-well plates at 2 × 10⁵ cells/well. Following 24 h adherence, cells were treated with LPS (Bioss, bs-8000P-5mg) in concentration- and time-dependent regimens: [1]. Untreated control (LPS-); [2]. 25 ng/mL LPS for 6 h; [3] 100 ng/mL LPS for 6 h; [4] 25 ng/mL LPS for 2 h; [5] 100 ng/mL LPS for 2 h. Cells were lysed for immunoblot analysis of p-NF-κB. p-NF-κB levels served as positive controls for nuclear translocation assays.

### Multiplex cytokine profiling

Conditioned media were collected from cells cultured in 6-cm dishes following 48 h serum starvation (3 mL serum-free medium). Cytokine concentrations were quantified using the ABplex Human 23-Plex Custom Panel (ABclonal, RK04352) on the ABplex-100 flow-based multiplex immunoassay system (ABclonal). 5 μL magnetic bead suspension was briefly combined with 50 μL standard or sample, followed by 1 h incubation at 37 °C. After magnetic separation and washing, a 50 μL detection antibody cocktail was added (37°C, 30 min), followed by streptavidin-PE conjugate incubation (37 °C, 15 min, light-protected). Fluorescence intensity was measured using dual-laser detection (488 nm and 635 nm excitation), with analyte concentrations calculated via five-parameter logistic regression (ABplex Analysis Software v2.0). Detailed statistical data about the experimental results are provided in Supplementary Table 8.

### IC50 and cell viability assays

Ovarian cancer cell lines were seeded in 96-well plates at optimized densities: HEY (1.2 × 10³), A2780 (3 × 10³), OVCAR8 (3 × 10³), SKOV3 (2.5 × 10³), ES-2 (1.5 × 10³), and OV90 (2.5 × 10³) cells/well. Following 24 h adherence, cells were treated with small molecule inhibitors at six concentrations: Napabucasin (0–3.2 μM), GSK126 (0–50 μM), Tazemetostat (0–150 μM), and Niraparib (0–75 μM). After 48 h incubation (37 °C, 5% CO₂), viability was assessed using CCK-8 reagent in serum-containing medium (10% v/v). A microplate reader measured absorption at 450 nm (reference 630 nm). Dose-response curves were generated via four-parameter logistic regression (GraphPad Prism v8.0). Detailed statistical data about the experimental results are provided in Supplementary Table 6.

### High-throughput compound screening

EZH2-oeNC and EZH2-OE HEY cells were seeded in 384-well plates (1 × 10³ cells/well) using an automated liquid handling system (Tecan Freedom EVO®). Following 24 h incubation (37 °C, 5% CO₂), compound libraries (2,188 FDA-approved drugs and 1962 kinase inhibitors, MCE) were dispensed at 1 μM final concentration via a robotic workstation (PerkinElmer Janus®). After 48 h of treatment, cell viability was assessed using CellTiter-Glo® Luminescent Cell Viability Assay (Promega, G7572) on a multimode plate reader (ImageXpress Nano). Using standardized operating protocols, screening was performed at the National Center for Translational Medicine (Ruijin Hospital, Shanghai Jiao Tong University School of Medicine).

### Animal studies

#### Orthotopic ovarian cancer model

Female C57BL/6 mice (5–6 weeks old) were obtained from JSJ (Shanghai) and maintained under specific pathogen-free conditions following the NIH Guide for the Care and Use of Laboratory Animals. The Institutional Animal Care and Use Committee of Tongji University School of Medicine approved all experimental protocols.

For tumor implantation, mice were anesthetized with tribromoethanol (AibeiBio, M2910; 10 μL/g, i.p.) After depilation and disinfection of the surgical area, a lower left or right abdominal incision was made to locate the ovary. A mixture of 50 μL Matrigel (Corning, 356234) and 50 μL cell suspension (2 × 10^6^ cells) was slowly injected into the ovary. After matrix polymerization, the peritoneum and skin were closed with 4-0 absorbable sutures (Ethicon). After gel solidification, the wound was sutured, and the mice were placed in a warm environment to recover. The wound was disinfected once daily for the first 2 days. Mice were euthanized by cervical dislocation after 6 weeks of regular breeding (weighed every week), followed by specimen collection and photography.

#### Intraperitoneal drug administration

On postoperative day 5, by referring to previous studies [66, 67], mice received intraperitoneal injections of Napabucasin (20 mg/kg) or vehicle control every other day for 37 days. Drug formulation was prepared immediately before administration: 7.2 mg Napabucasin was dissolved in 360 μL DMSO (20 mg/mL stock), followed by sequential addition of 1440 μL PEG300, 180 μL Tween80, and 1620 μL sterile saline (0.9% NaCl), with vortex mixing until complete solubilization. Vehicle control contained equivalent volumes of excipients without drugs. Injections (200 μL) were administered in the lower left abdominal quadrant using a 27-gauge needle, alternating sides between treatments. Mice were euthanized by cervical dislocation under anesthesia (tribromoethanol, i.p.). Tumor tissues were harvested for H&E staining and IHC. Detailed statistical data about the experimental results are provided in Supplementary Table 8.

### Statistics and reproducibility

Data were analyzed using SPSS 22.0 or GraphPad Prism 8.0. An unpaired t-test was used for two-group comparisons, and one-way ANOVA for multiple groups. Graphs show means ± s.e.m. unless stated otherwise. Sample sizes were not pre-determined statistically. No data were excluded, and experiments were not blinded. All experiments were performed at least thrice independently. For in vivo studies, mice were randomly grouped after tumor cell injection. Researchers conducting mouse experiments were not blinded to the hypotheses. Histological staining quantification was done blindly, and image analysis software was used for unbiased analysis of batch-processed images. Statistical significance was set at *p* < 0.05, with exact levels indicated. Figure legends specify statistical tests and sample sizes (*n*).

## Supplementary information

supFigure 1

supFigure 2

supFigure 3

supFigure 4

supFigure 5

supFigure 6

supFigure 7

supFigure 8

supFigure 9

Supplementary Material Legends

Original Data

Supplementary Table 1

Supplementary Table 2

Supplementary Table 3

Supplementary Table 4

Supplementary Table 5

Supplementary Table 6

Supplementary Table 7

Supplementary Table 8

Supplementary Table 9

## Data Availability

The data or reagents are available from the corresponding author upon reasonable request.
