# Supplementary figures and images for: Targeting EZH2-driven cholesterol metabolic vulnerability through Napabucasin suppresses ovarian cancer metastasis
Source: Cell Death Dis. 2026 Jun 27;17(1):603. doi: 10.1038/s41419-026-08894-9 (PMC13315729; doi:10.1038/s41419-026-08894-9)

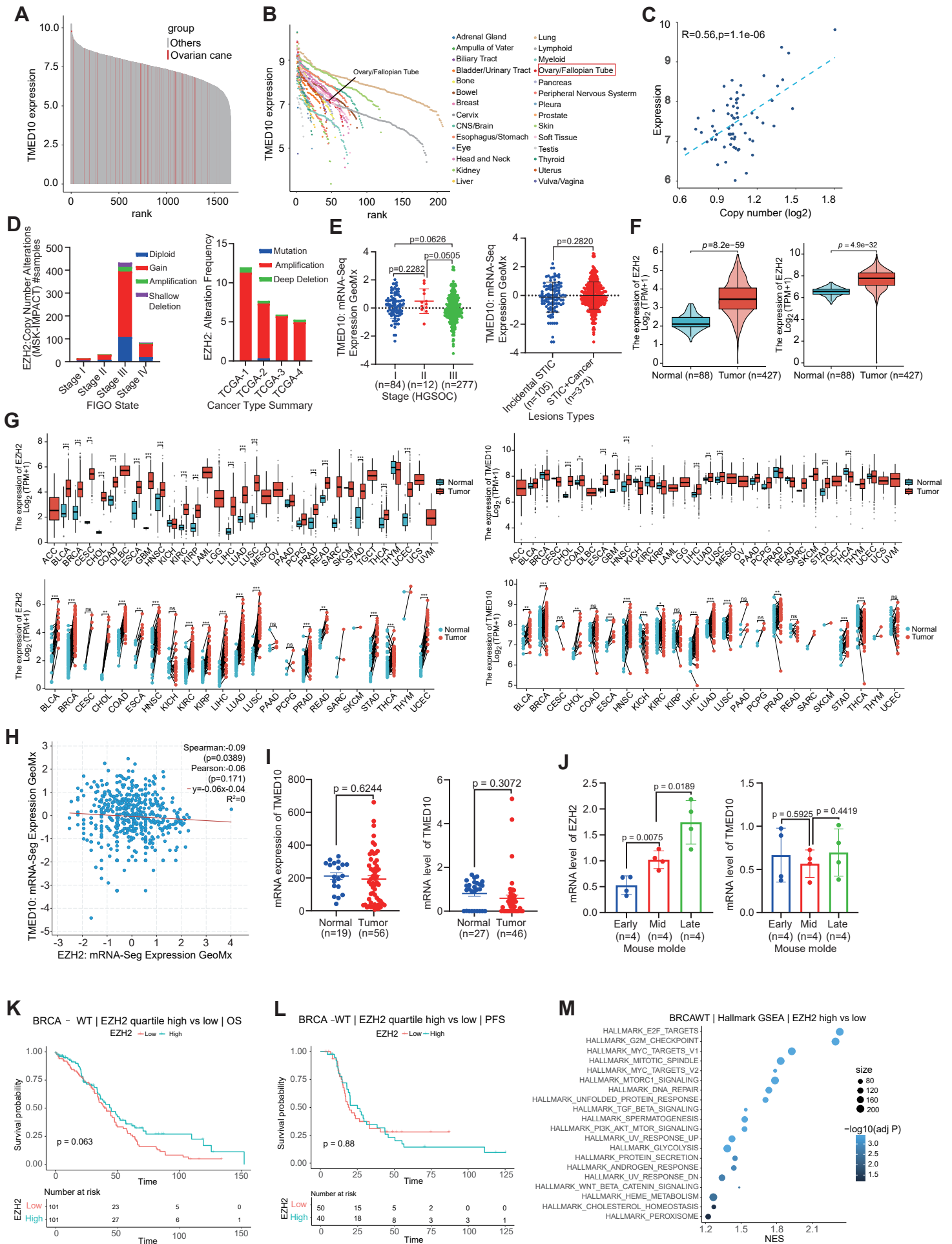

Supplement: Supplementary file 1 — supFigure 1 [file 41419_2026_8894_MOESM1_ESM.pdf]

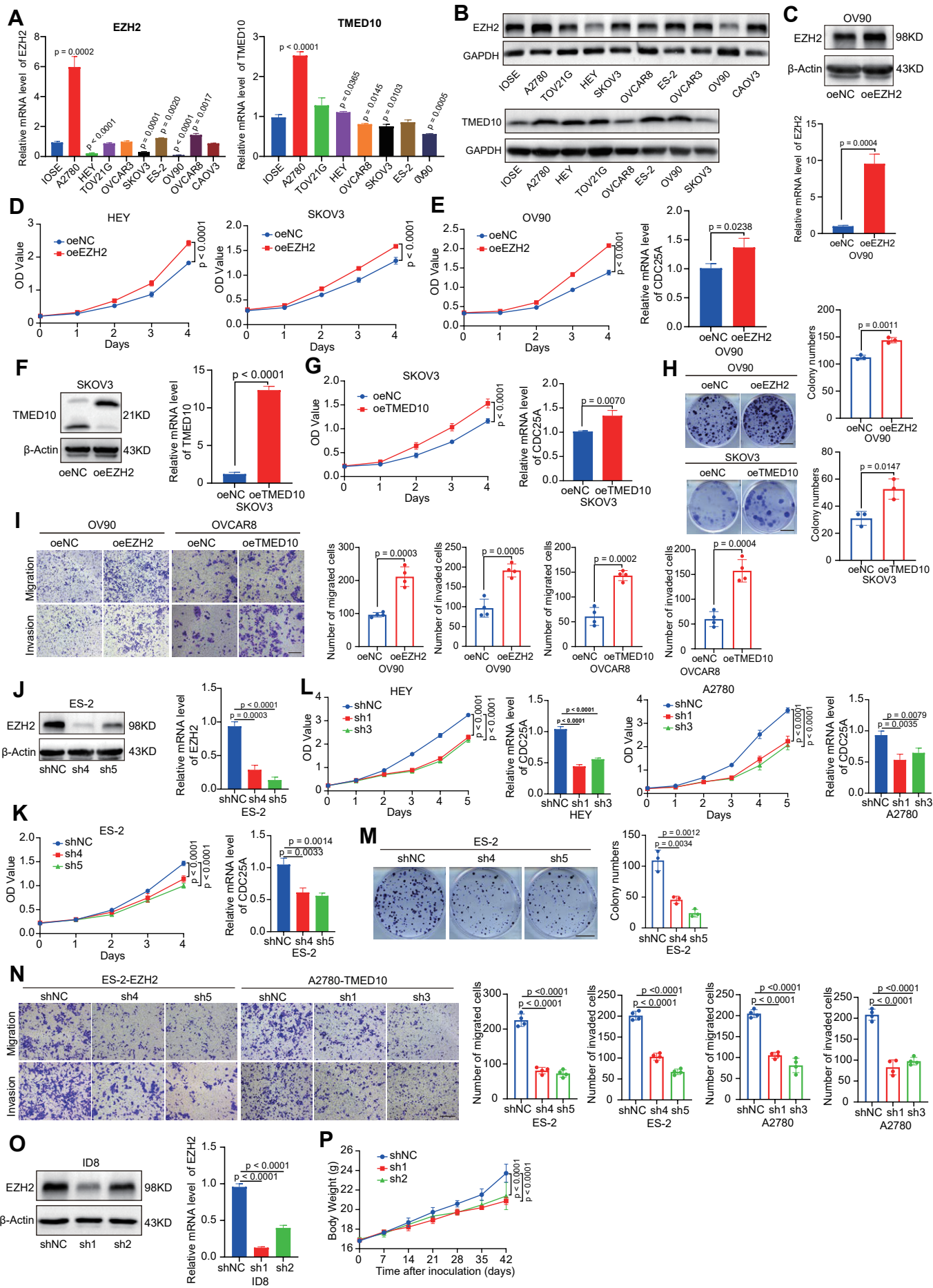

Supplement: Supplementary file 2 — supFigure 2 [file 41419_2026_8894_MOESM2_ESM.pdf]

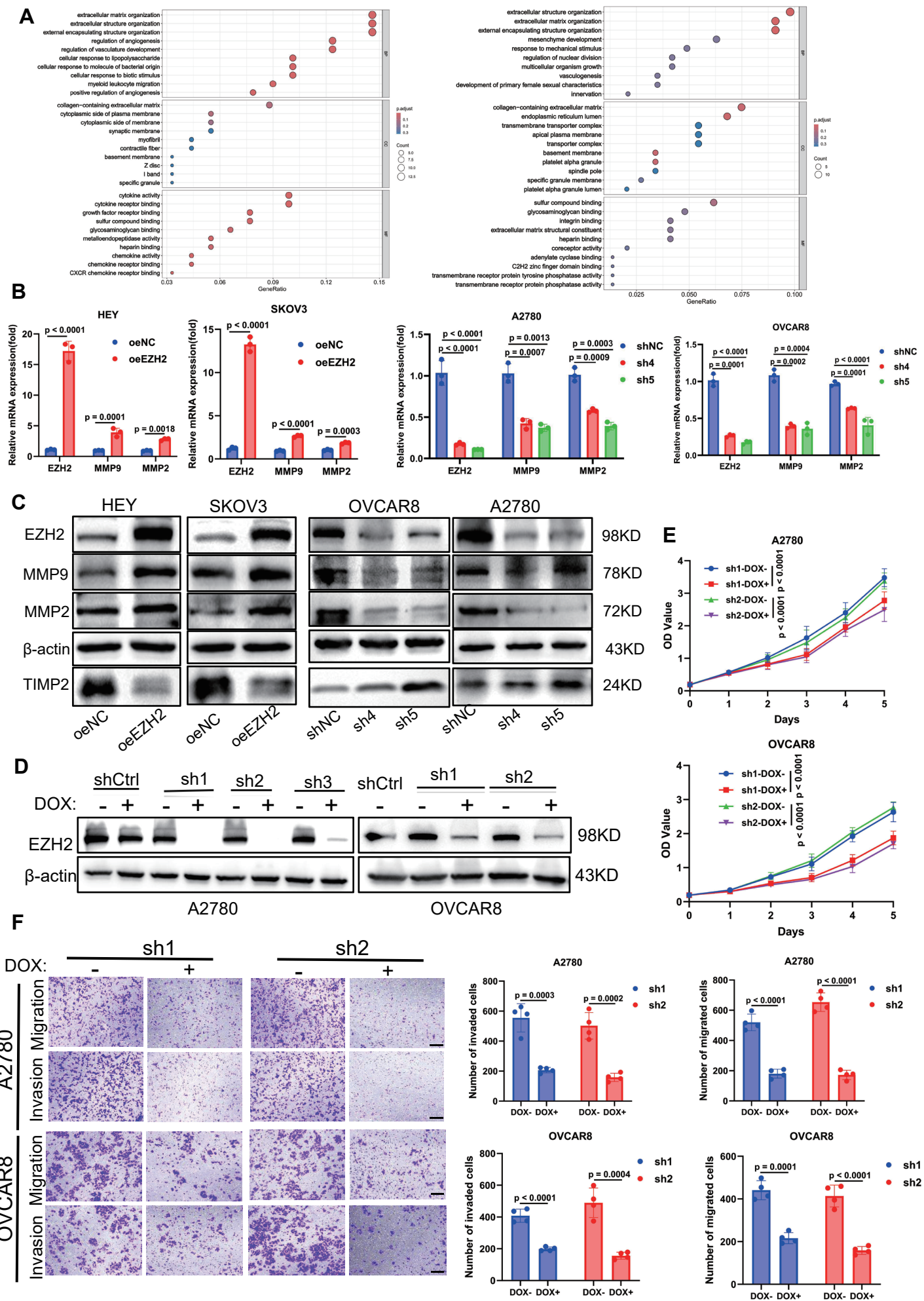

Supplement: Supplementary file 3 — supFigure 3 [file 41419_2026_8894_MOESM3_ESM.pdf]

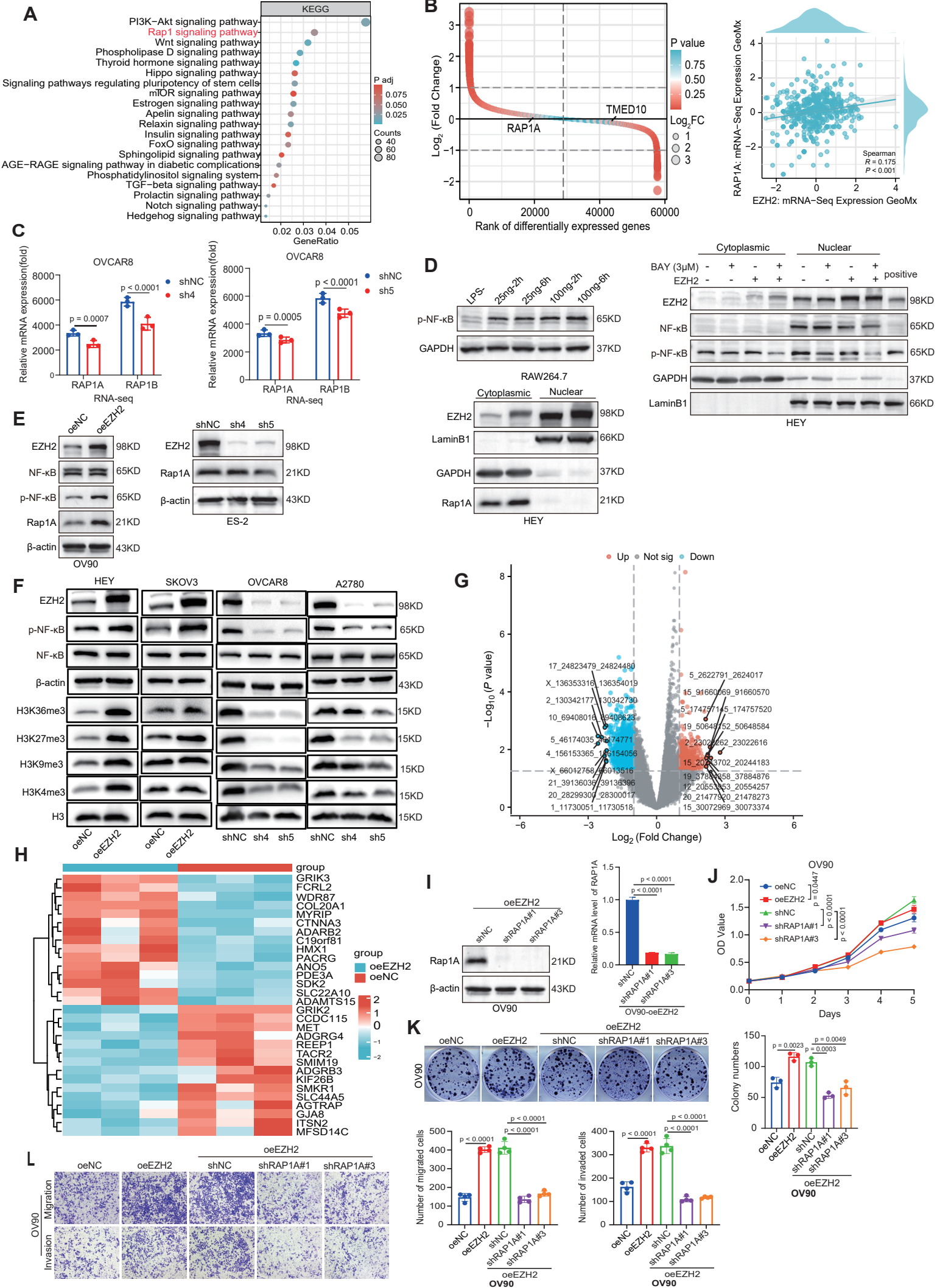

Supplement: Supplementary file 4 — supFigure 4 [file 41419_2026_8894_MOESM4_ESM.pdf]

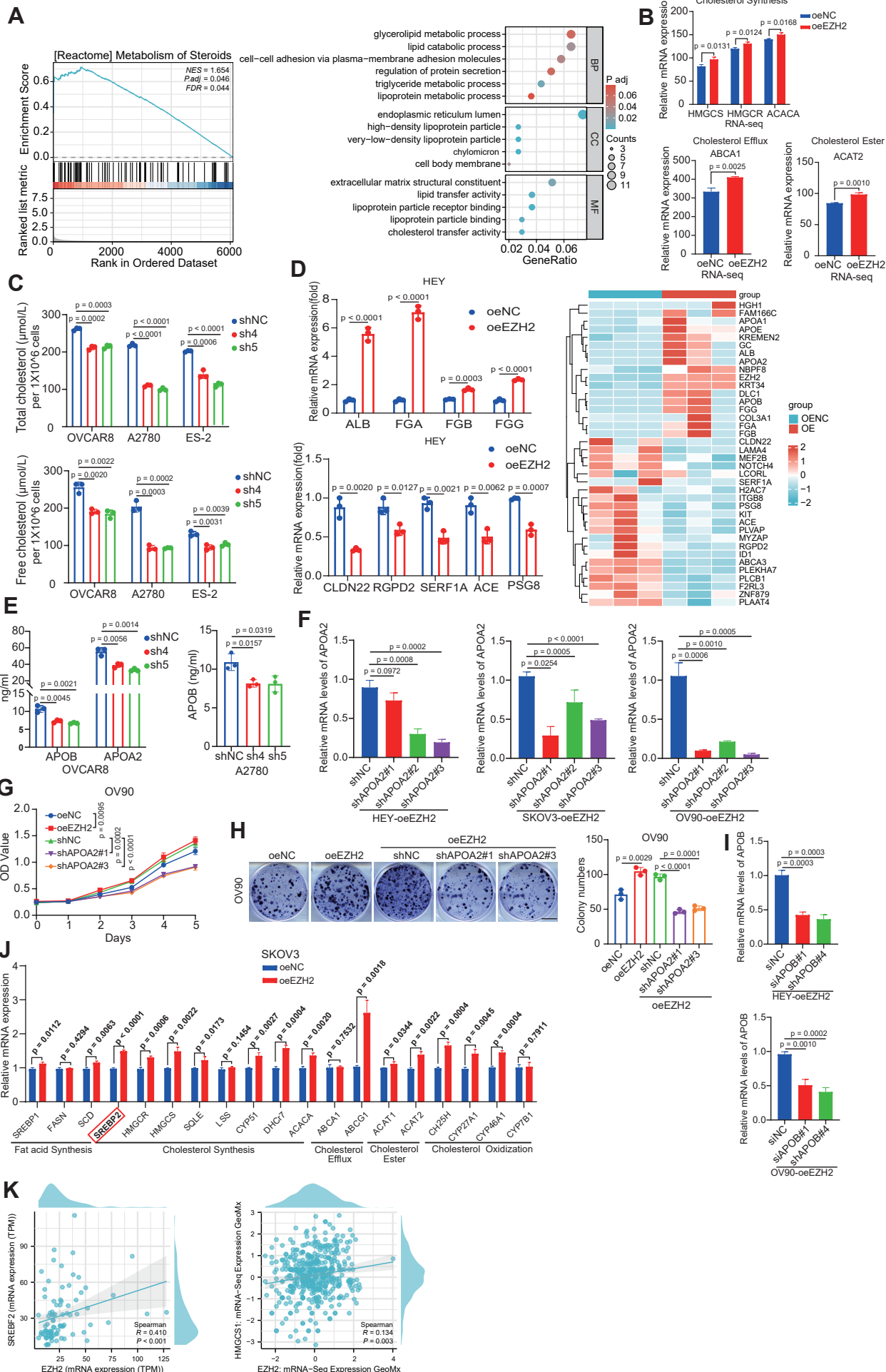

Supplement: Supplementary file 5 — supFigure 5 [file 41419_2026_8894_MOESM5_ESM.pdf]

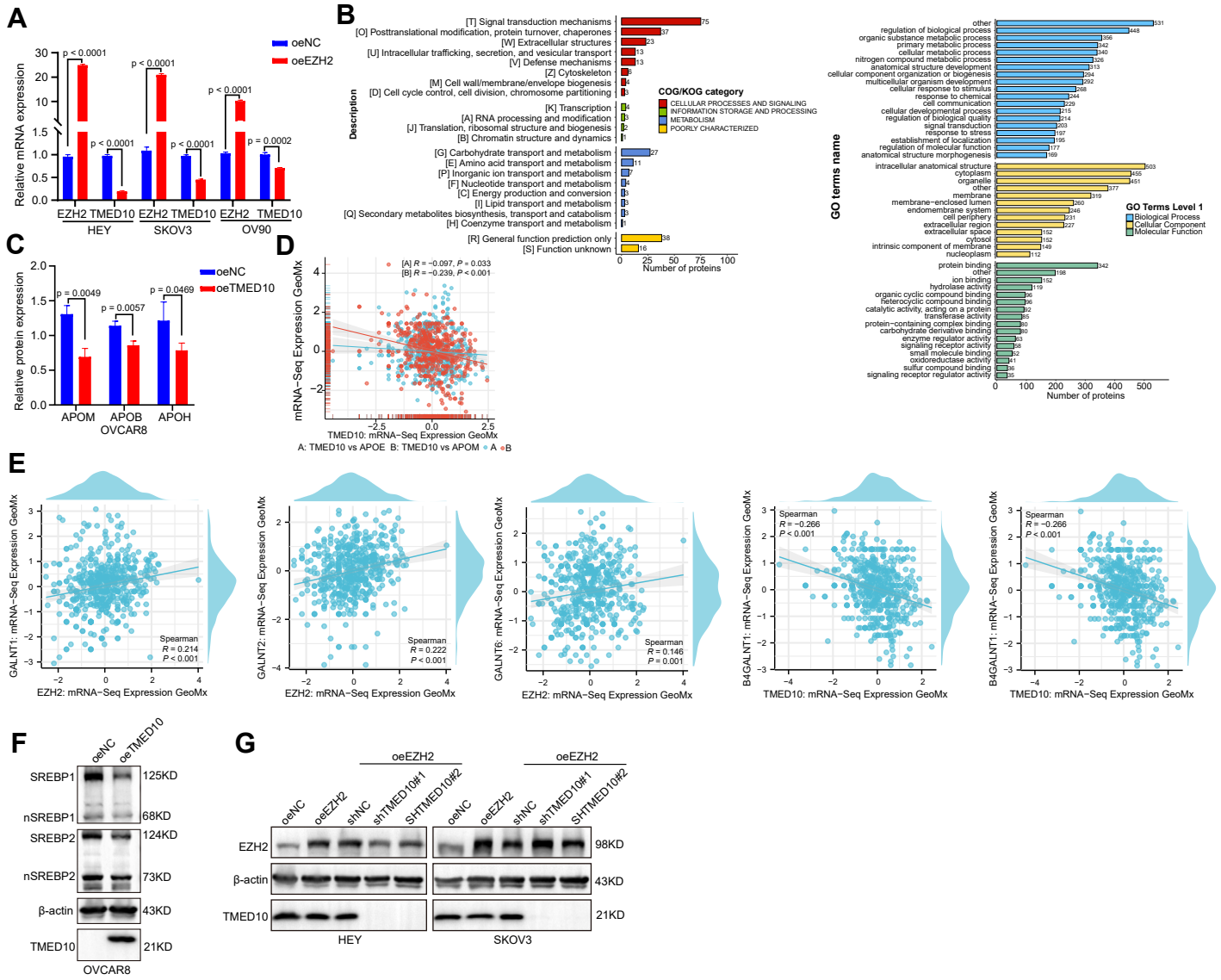

Supplement: Supplementary file 6 — supFigure 6 [file 41419_2026_8894_MOESM6_ESM.pdf]

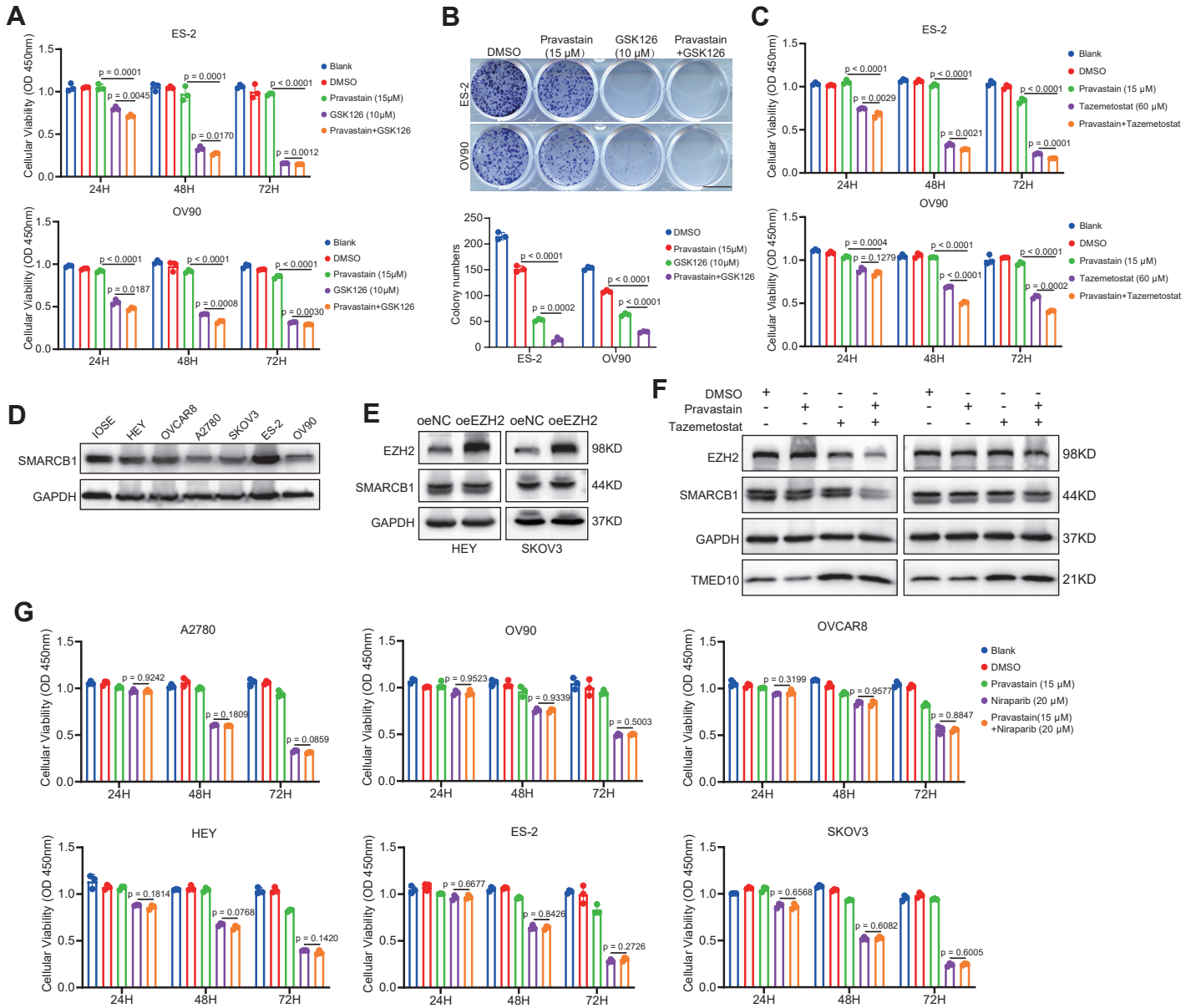

Supplement: Supplementary file 7 — supFigure 7 [file 41419_2026_8894_MOESM7_ESM.pdf]

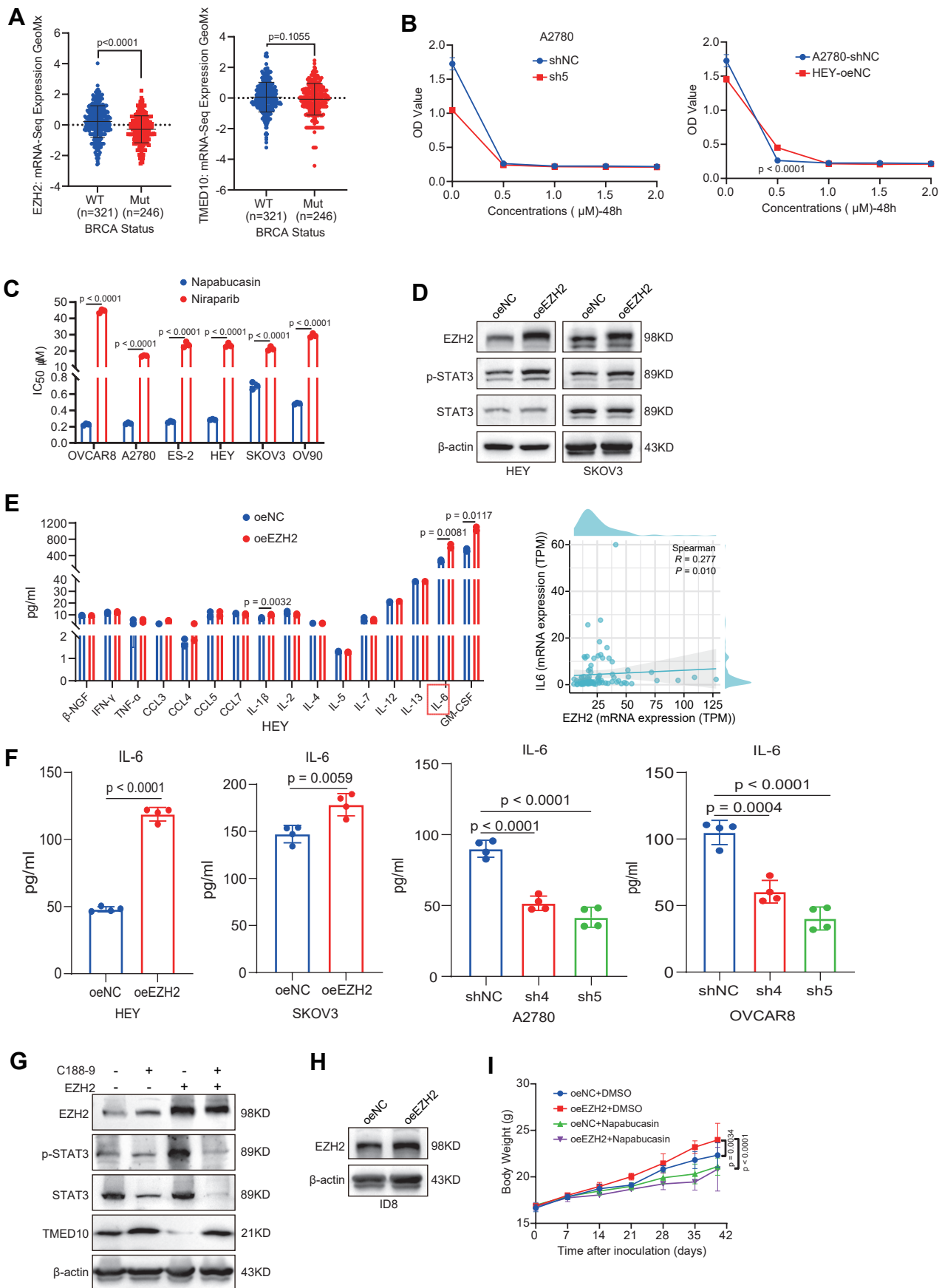

Supplement: Supplementary file 8 — supFigure 8 [file 41419_2026_8894_MOESM8_ESM.pdf]

**A**

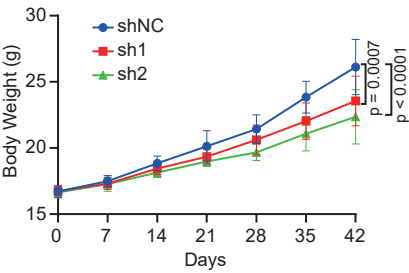

**B**

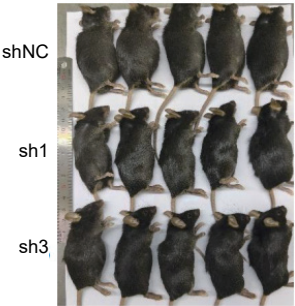

**C**

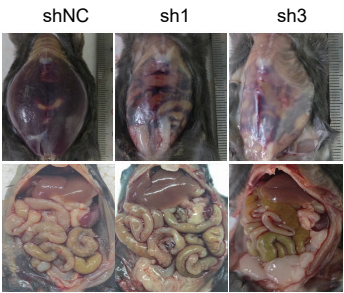

**D**

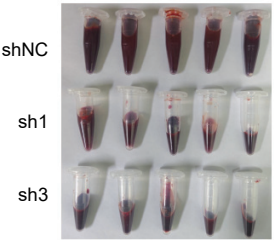

Supplement: Supplementary file 9 — supFigure 9 [file 41419_2026_8894_MOESM9_ESM.pdf]
