# Supplementary material for: Targeting EZH2-driven cholesterol metabolic vulnerability through Napabucasin suppresses ovarian cancer metastasis
Source: Cell Death Dis. 2026 Jun 27;17(1):603. doi: 10.1038/s41419-026-08894-9 (PMC13315729; doi:10.1038/s41419-026-08894-9)
Supplement: Supplementary file 10 — Supplementary Material Legends [file 41419_2026_8894_MOESM10_ESM.docx]

**Supplementary material legends**

**Supplementary Fig. 1**

**A**, All cell lines annotated in the Cancer Cell Line Encyclopedia (CCLE) were arranged according to TMED10 transcript levels from the highest (left) to the lowest (right). Each line represents a different cell line. Ovarian cancer cell lines are denoted by red lines. **B**, All cell lines annotated in the CCLE were grouped according to the cancer type. For each cancer type, cell lines were arranged according to the levels of TMED10 transcripts, from highest to lowest. Each dot represents a different cell line. **C**, Correlation between the TMED10 gene copy number and gene expression in ovarian cancer cell lines from the CCLE. Each dot represents a different cell line. Pearson correlation coefficient and p-value are shown. **D**, In the cBioPortal data, the copy number variation of EZH2 in different stages of ovarian cancer according to FIGO staging (left), and the copy number variation of EZH2 in different types of ovarian cancer in the TCGA data (right). **E**, TMED10 expression in different stages of HGSOC (left) and in different stages of ovarian cancer progression (STIC, and invasive ovarian carcinoma; right) in the cBioPortal data (id=Ovary/Fallopian_OC_2024). **F**, Comparative analysis of EZH2 (left) or TMED10 (right) expression between tumor tissues (TCGA database) and normal tissues (GTEx database) across ovarian cancer. **G**, Paired or unpaired tumor-normal analysis of EZH2 (left) or TMED10 (right) expression in matched specimens from the TCGA pan-cancer cohort. Data were analyzed by a two-tailed unpaired t-test. **H**, Correlation between the EZH2 and TMED10 gene expression (normalized FPKM) in ovarian cancer from the cBioPortal data (id=Ovary/Fallopian_OC_2024). **I**, RNA-Seq analysis of TMED10 mRNA expression in 19 normal tissues and 56 ovarian cancer (OC) tissues (left) and RT-qPCR validation of TMED10 mRNA levels in 27 normal and 46 OC tissues (right). **J**, EZH2 (left) or TMED10 (right) mRNA expression in ovarian cancer tissues at different progression stages (Early-stage: n=4, Mid-stage: n=4, Late-stage: n=4) from mouse models. **K,** Kaplan–Meier overall survival (OS) analysis in BRCA-wild-type (BRCA-WT) high-grade serous ovarian cancer patients from the TCGA/cBioPortal cohort, stratified by EZH2 mRNA expression. Tick marks indicate censored cases. The table below the plot shows the number of patients at risk at the indicated time points. Statistical significance was assessed by log-rank test. **L,** Kaplan–Meier progression-free survival (PFS) analysis in BRCA-wild-type (BRCA-WT) high-grade serous ovarian cancer patients from the TCGA/cBioPortal cohort, stratified by EZH2 mRNA expression. Tick marks indicate censored cases. The table below the plot shows the number of patients at risk at the indicated time points. Statistical significance was assessed by log-rank test. **M,** Hallmark gene set enrichment analysis (GSEA) was performed on the TCGA high-grade serous ovarian cancer BRCA-wild-type subgroup after stratifying tumors by EZH2 mRNA expression. The dot plot shows the top 20 Hallmark pathways ranked by adjusted significance. Dot position indicates normalized enrichment score (NES), dot size indicates pathway size, and dot color indicates -log10(adjusted P value). Positive NES values indicate enrichment in EZH2-high tumors. For **E-G, I. J**, Data represent the mean ± s.d., and statistical analyses were performed using two-tailed unpaired t-tests.

**Supplementary Fig. 2**

**A**, EZH2 (left) or TMED10 (right) mRNA expression levels in normal ovarian epithelial cell line IOSE and ovarian cancer (OV) cell lines. Data are derived from three independent biological replicates. **B**, EZH2 (up) or TMED10 (down) protein expression in IOSE and OV cell lines. Representative immunoblots from two independent experiments are shown. **C**, Immunoblotting and RT-qPCR analysis of EZH2 overexpression in OV90 cells transfected with OE or control (oeNC) constructs. Representative data from at least three independent experiments are shown. **D**, Cell viability of HEY-oeEZH2 (left) and SKOV3-oeEZH2 versus oeNC cells over a 4-day culture period. The data represent three independent biological replicates. **E**, Cell viability of OV90-oeEZH2 and oeNC cells over a 4-day culture period. The data represent three independent biological replicates. **F**, Immunoblotting and RT-qPCR analysis of TMED10 overexpression in SKOV3 cells transfected with OE or control (oeNC) constructs. Representative data from at least three independent experiments are shown. **G**, Cell viability of SKOV3-oeTMED10 and oeNC cells over a 4-day culture period. The data represent three independent biological replicates. **H**, Clonogenic capacity of OV90-oeEZH2 (up) and SKOV3-oeTMED10 compared to oeNC cells after 14-day culture. Scale bar, 200 μm. Data are from three independent biological replicates. **I**, Migration and invasion abilities of OV90-oeEZH2 (left) and OVCAR8-oeTMED10 compared to oeNC cells. Representative images of migrated/invaded cells are shown. Scale bar, 100 μm. Data represent three independent biological replicates. **J**, Immunoblotting and RT-qPCR analysis of EZH2 knockdown in ES-2 cells using two independent shRNAs or shNC. Representative immunoblots from at least three experiments are shown. **K**, Cell viability of ES-2-shEZH2 and shNC cells during 4-day culture. Data are derived from three independent biological replicates. **L**, Cell viability of HEY (left) and A2780 (right)-shTMED10 versus shNC cells during 5-day culture. Data are derived from three independent biological replicates. **M**, Colony formation capacity of ES-2-shNC and shEZH2 cells after 14-day culture. Scale bar, 200 μm. Data represent three independent biological replicates. **N**, Migration and invasion assays of ES-2- shEZH2 (left) and A2780-shTMED10 (right) compared to shNC cells. Representative images are shown. Scale bar, 100 μm. Data are from three independent biological replicates. **O**, Immunoblotting and RT-qPCR validation of EZH2 knockdown in ID8 cells using two shRNAs or shNC. Representative immunoblots from at least three experiments are shown. **P**, Body weight of mice bearing ID8-shEZH2 or shNC tumors (n=5 mice per group; single experiment). Weights were recorded weekly. For **A, C-O**, Data represent the mean ± s.d., and statistical analyses were performed using two-tailed unpaired t-tests.

**Supplementary Fig. 3**

**A**, GO enrichment analysis of RNA-seq data of EZH2- knockdown OVCAR8 cells (sh4 vs shNC: left; sh5 vs shNC: right). **B**, MMP2 and MMP9 mRNA expression levels in HEY and SKOV3 cells with EZH2 overexpression, and in A2780 and OCAR8 cells with EZH2 knockdown. Data are derived from three independent biological replicates. **C**, Protein expression levels of MMP2 and MMP9 in HEY and SKOV3 cells with EZH2 overexpression (left) and A2780 and OCAR8 cells with EZH2 knockdown (right) were examined by immunoblotting. Blots are representative of at least two independent experiments. **D**, The knockdown efficiency of EZH2 in A2780 and OVCAR8 after 72 hours of DOX induction was detected by immunoblotting compared with DOX-control. Representative immunoblots from at least three independent experiments are shown. **E**, In A2780 and OVCAR8 cells, compared with the DOX-negative group, the cell viability was detected by CCK-8 after 72 hours of DOX-induced EZH2 knockdown for 5 days. Data represent three independent biological replicates. **F**, In A2780 and OVCAR8 cells, migration and invasion assays were performed after knockdown of EZH2 by DOX for 72 h compared with DOX-negative cells. Representative images are shown. Scale bar, 100 μm. Data are from three independent biological replicates. For **B**, **F**, Data represent the mean ± s.d., and statistical analyses were performed using two-tailed unpaired t-tests. For growth curve analysis involving multiple time points, repeated measures one-way ANOVA was used to evaluate the interaction between time and group, followed by Bonferroni's post hoc test for pairwise comparisons at each time point.

**Supplementary Fig. 4**

**A**, KEGG pathway enrichment analysis of differentially expressed genes upregulated with TMED10 in ovarian carcinomas (TCGA data). **B**, Ranked list plot of differentially expressed genes associated with EZH2 in ovarian carcinoma RNA-seq data (TCGA data) (left); Correlation between the EZH2 and RAP1A gene expression (normalized FPKM) in ovarian cancer from the cBioPortal data (id=Ovary/Fallopian_OC_2024) (right). **C**, Expression levels of RAP1A and RAP1B in RNA-Seq results after EZH2 knockdown in OVCAR8 cells. **D**, Immunoblotting analysis of p-NF-κB protein levels in RAW264.7 cells treated with LPS at varying concentrations (0, 25, 100 ng/mL) for different durations (0, 2, 6 h) (top); subcellular localization and expression changes of EZH2, RAP1A in HEY-oeEZH2 and oeNC cells (bottom); subcellular localization and expression changes of EZH2, p-NF-κB, and NF-κB in HEY oeEZH2 and OENC cells treated with BAY (3 μM, 48 h) (right). Representative immunoblots from at least three independent experiments are shown.  **E**, Immunoblotting analysis of RAP1A protein levels in ES-2 shEZH2 and shNC cells (right), and p-NF-κB, NF-κB, and Rap1A protein levels in VO90-oeEZH2 and oeNC cells (left). Blots are representative of at least three independent experiments. **F**, Immunoblotting shows changes in the protein levels of p-NF-κB, NF-κB, H3k36me3, H3k27me3, H3k9me3, and H3k46me3 after EZH2 overexpression in HEY and SKOV3 cells (left), and after EZH2 knockdown in OVCAR8 and A2780 cells, Changes in the expression levels of these proteins (right). Representative immunoblots from at least three experiments are shown.

**G**, Volcano plot shows the difference peaks (padj<0.05, |log₂FC|>1) in CHIP-Seq results of HEY-oeNC cells and HEY-oeEZH2 cells, and shows the top 10 most significant difference peaks. Both oeEZH2 and oeNC groups contain three biological replicates, assessed as one experiment. **H**, Heat map showing the top 15 significantly altered genes when EZH2 was knocked down in OVCAR8 cells. **I**, Immunoblotting and RT-qPCR validation of Rap1A knockdown in EZH2-overexpressing OV90 cells using two independent shRNAs or shNC. Representative immunoblots from at least three experiments are shown. **J**, Cell viability of EZH2-overexpressing OV90 cells with Rap1A knockdown shRNA or control (shNC) during 5-day culture. Data represent three independent biological replicates. **K**, Clonogenic capacity of Rap1A-knockdown OV90-oeEZH2 cells after 14-day culture. Scale bar, 200 μm. Data are derived from three independent biological replicates. **L**, Migration and invasion abilities of Rap1-knockdown OV90 oeEZH2 cells. Representative images of migrated/invaded cells are shown. Scale bar, 100 μm. Data are from three independent biological replicates. For **C**, **I-L**, Data represent the mean ± s.d., and statistical analyses were performed using two-tailed unpaired t-tests.

**Supplementary Fig. 5**

**A**, GSEA of upregulated pathways in HEY EZH2-OE cells; NES, normalized enrichment score. Data were analyzed using a two-sided permutation test, with P-values adjusted using the Benjamini-Hochberg method (left); GO enrichment analysis of upregulated genes from RNA-Seq in HEY-oeEZH2 and oeNC cells (right). **B**, mRNA expression levels of cholesterol synthesis, efflux, and esterification-related genes from RNA-Seq in HEY-oeEZH2 and oeNC cells. **C**, Total cholesterol and free cholesterol levels in EZH2-KD OVCAR8, A2780, and ES-2 cells. Data are derived from three independent biological replicates. **D**, RT-qPCR was used to detect the expression of significantly differentially expressed genes in the RNA-seq results after overexpression of EZH2.Data are derived from three independent biological replicates (left). Heatmap of the top 38 DEGs of the up (n = 19) and down (n = 19) of RNA-Seq in HEY-oeEZH2 and oeNC cells (right). **E**, As determined by ELISA, APOB and APOA2 levels in conditioned media were collected from OVCAR8 and A2780 cells knockdown EZH2 following 48-hour culture. Data are derived from three independent biological replicates. **F**, RT-qPCR analysis of APOA2 mRNA knockdown levels in EZH2-OE HEY, SKOV3 and OV90 cells. Data from three independent experiments. **G**, Viability of EZH2-OE OV90 cells transfected with two independent shAPOA2 constructs or non-targeting control (shNC) over 5 days. Data from three independent cultures. **H**, Clonogenic capacity of OV90 EZH2-OE cells with APOA2 knockdown after 14 days; scale bar, 200 μm; data from three independent cultures. **I**, RT-qPCR validation of APOB knockdown (siAPOB) in EZH2-OE HEY and OV90 cells. Data from three independent experiments. **J**, RT-qPCR analysis of mRNA expression levels of cholesterol synthesis, efflux, esterification, and oxidation-related genes in EZH2-OE SKOV3 cells. Data are from three independent biological replicates. **K**, Correlation between the EZH2 and SREBF2 or HMGCS1 gene expression (normalized FPKM) in ovarian cancer from the cBioPortal data (id=Ovary/Fallopian_OC_2024). For **B**, **C-F**, **H**, **J**, Data represent the mean ± s.d., and statistical analyses were performed using two-tailed unpaired t-tests. For **G**, Data analysis involving multiple time points, repeated measures one-way ANOVA was used to evaluate the interaction between time and group, followed by Bonferroni's post hoc test for pairwise comparisons at each time point.

**Supplementary Fig. 6**

**A**, RT-qPCR analysis of TMED10 mRNA levels in EZH2-OE HEY, SKOV3 and OV90 cells. Data from three independent experiments. **B**, COG/KOG classification of down-regulated proteins in proteomic sequencing of OVCAR8 TMED10-OE and oeNC cell culture supernatants (left). GO enrichment analysis of proteomic sequencing data from conditioned media of TMED10-overexpressing OVCAR8 cells (right). **C**, Proteomic sequencing analysis of APOB, APOM, and APOH protein levels in TMED10-modulated cells. **D**, Correlation between the TMED10 and APOE or APOM gene expression (normalized FPKM) in ovarian cancer from the cBioPortal data (id=Ovary/Fallopian_OC_2024). **E**, Correlation between the EZH2 and GALNT1, GALNT2, or GALNT6 gene expression; TMED10 and B4GALNT1 or GALNT18 gene expression (normalized FPKM) in ovarian cancer from the cBioPortal data (id=Ovary/Fallopian_OC_2024). **F**, Immunoblotting analysis of SREBP2 (full-length), nSREBP2 (nuclear SREBP2), SREBP1 (full-length), and nSREBP1 (nuclear SREBP1) protein levels in TMED10-expressing OVCAR8 cells. Representative immunoblots from at least two experiments are shown. **G**, TMED10 knockdown validation in EZH2-OE HEY and SKOV3 cells using two shRNAs or shNC. Representative immunoblots from at least two experiments are shown. For **A, C**, Data represent the mean ± s.d., and statistical analyses were performed using two-tailed unpaired t-tests.

**Supplementary Fig. 7**

**A**, Cell viability in low EZH2-expressing OV90 cells (bottom), and high EZH2-expressing ES-2 cells (top) treated with Pravastatin (15 μM), GSK126 (10 μM), or their combination for 24 h, 48 h, or 72 h; n = 3 biological replicates, assessed as one experiment. **B**, Clonogenic capacity of ES-2 and OV90 cells treated with Pravastatin (15 μM), GSK126 (10 μM), or their combination for 7 days; scale bar, 200 μm; add medicine the next day, data from three independent cultures. **C**, Cell viability in low EZH2-expressing OV90 cells (bottom), and high EZH2-expressing ES-2 cells (top) treated with Pravastatin (15 μM), Tazemetostat (60 μM), or their combination for 24 h, 48 h, or 72 h; n = 3 biological replicates, assessed as one experiment. **D**, Immunoblotting analysis of SMARCB1 in ovarian cancer cells compared to IOSE cells. Images are representative of three independent experiments. **E**, Immunoblotting analysis of EZH2 and SMARCB1 in HEY and SKOV3-oeEZH2 cells compared to oeNC cells. Images are representative of three independent experiments. **F**, Immunoblotting analysis of EZH2, TMED10, and SMARCB1 in ES-2 and OV90 cells treated with Pravastatin (15 μM), Tazemetostat (60 μM), or their combination for 72 h. Images are representative of two independent experiments. **G**, Cell viability in six ovarian cancer cells treated with Pravastatin (15 μM), Niraparib (20 μM), or their combination for 24 h, 48 h, or 72 h; n = 3 biological replicates, assessed as one experiment. For **A-C, G**, Data represent the mean ± s.d., and statistical analyses were performed using two-tailed unpaired t-tests.

**Supplementary Fig. 8**

**A**, EZH2 or TMED10 mRNA expression in BRCA wild-type (WT) versus BRCA-mutated (Mut) ovarian carcinoma patients (cBioPortal data, id=Ovary/Fallopian_OC_2024). The original data are shown in the Supplementary Table 2, 3. **B**, Napabucasin sensitivity at varying concentrations (48 h treatment) in A2780 EZH2-KD and shNC cells (left); Napabucasin sensitivity at varying concentrations (48 h treatment) in A2780-shNC (EZH2-high) compared to HEY-oeNC (EZH2-low) cells (right). Data represent three independent biological replicates. **C**, IC50 values of Napabucasin and Niraparib against cell viability of EZH2-high (OVCAR8, A2780, ES-2) and EZH2-low (HEY, SKOV3, OV90) cell groups; n = 3 biological replicates, assessed as one experiment. **D**, STAT3 and p-STAT3 protein levels in EZH2-OE HEY and SKOV3 cells. Representative immunoblots from at least two experiments are shown. **E**, Flow cytometry-based multiplex analysis of cytokine levels in conditioned media from EZH2-OE HEY cells cultured for 48 h (left); n=2, data are representative of two independent experiments. Correlation between the EZH2 and IL-6 gene expression in ovarian cancer from the cBioPortal data (right) (id=Ovary/Fallopian_OC_2024). **F**, IL-6 secretion levels were measured by ELISA in EZH2‑overexpressing HEY and SKOV3 cells, as well as in EZH2‑knockdown A2780 and OVCAR8 cells. Data are from three independent biological replicates. **G**, Immunoblotting analysis of EZH2, STAT3, p-STAT3, and TMED10 protein levels in EZH2-OE HEY cells with C188-9 (25μm, 48 h) treatment. Representative immunoblots from two independent experiments are shown. **H**, Immunoblotting analysis of EZH2 overexpression efficiency in ID8 cells. Representative immunoblots from three independent experiments are shown. **I**, Body weight monitoring of ID8 oeEZH2 and oeNC tumor-bearing mice treated with DMSO or Napabucasin (20 mg/kg, 200 μL/dose, every other day, i.p.) (n=6 mice per group; single experiment). Weights were measured weekly. For **A, C-F**, Data represent the mean ± s.d., and statistical analyses were performed using two-tailed unpaired t-tests. For **B, I,** Data were analyzed by one-way ANOVA with an LSD test.

**Supplementary Fig. 9**

**A**, Body weight of mice bearing ID8-shEZH2 or shNC tumors (n=5 mice per group; single experiment). Weights were recorded weekly. **B**, Representative images of C57BL/6 mice that were orthotopically injected with ID8‑shNC, ID8‑sh1, or ID8‑sh2 cells and euthanized 42 days post‑injection (n=5 mice per group; single experiment). **C**, Representative images of ascites and intraperitoneal metastasis in C57BL/6 mice orthotopically injected with ID8‑shNC, ID8‑sh1, or ID8‑sh2 cells and euthanized 42 days post‑injection (n=5 mice per group; single experiment). **D**, Compared with ID8‑shNC controls, ascites production was reduced in mice following EZH2 knockdown. For **A,** Data were analyzed by one-way ANOVA with an LSD test.

**Table legends**

**Supplementary Table 01.** Summary of all cell lines, primers, antibodies, and plasmid sequences employed in this study.

**Supplementary Table 02.** Expression levels of EZH2 and TMED10 from RNA‑seq of clinical samples, clinicopathological information of cases subjected to immunohistochemical analysis, and expression levels of EZH2 and TMED10 in mouse samples.

**Supplementary Table 03.** Expression levels of EZH2 and TMED10 in cBioPortal data across different tumor types, ovarian cancer FIGO stages and progression stages, as well as according to BRCA mutation status; and prognostic analysis of EZH2 in the BRCA-WT subgroup.

**Supplementary Table 04.** RNA‑seq data of EZH2/TMED10‑high versus EZH2/TMED10‑low groups from the TCGA database, and expression levels of EZH2 and TMED10 in ovarian cancer and pan‑cancer cohorts.

**Supplementary Table 05.** Raw data of all RT‑qPCR results and the mass spectrometry raw data for SKOV3‑oeEZH2 and OVCAR8‑oeTMED10 cells used in this study.

**Supplementary Table 06.** Raw data of CCK‑8, Cell counts, Transwell, colony formation, and IC50 assays analyzed in this study.

**Supplementary Table 07.** RNA‑seq data and enrichment analysis results of HEY‑oeNC versus HEY‑oeEZH2 cells, as well as proteomics data and enrichment analysis results of OVCAR8‑oeNC versus OVCAR8‑oeTMED10 cells.

**Supplementary Table 08.** Raw data of ELISA, metabolic probe assays, multiplex cytokine assays, and animal experiment analyses performed in this study.

**Supplementary Table 09.** RNA‑seq analysis data of OVCAR8‑shNC versus sh4/sh5 cells, and ChIP‑seq analysis data of HEY‑oeNC versus HEY‑oeEZH2 cells.
