# Supplementary material for: Targeting EZH2-driven cholesterol metabolic vulnerability through Napabucasin suppresses ovarian cancer metastasis
Source: Cell Death Dis. 2026 Jun 27;17(1):603. doi: 10.1038/s41419-026-08894-9 (PMC13315729; doi:10.1038/s41419-026-08894-9)

Fig.1 K

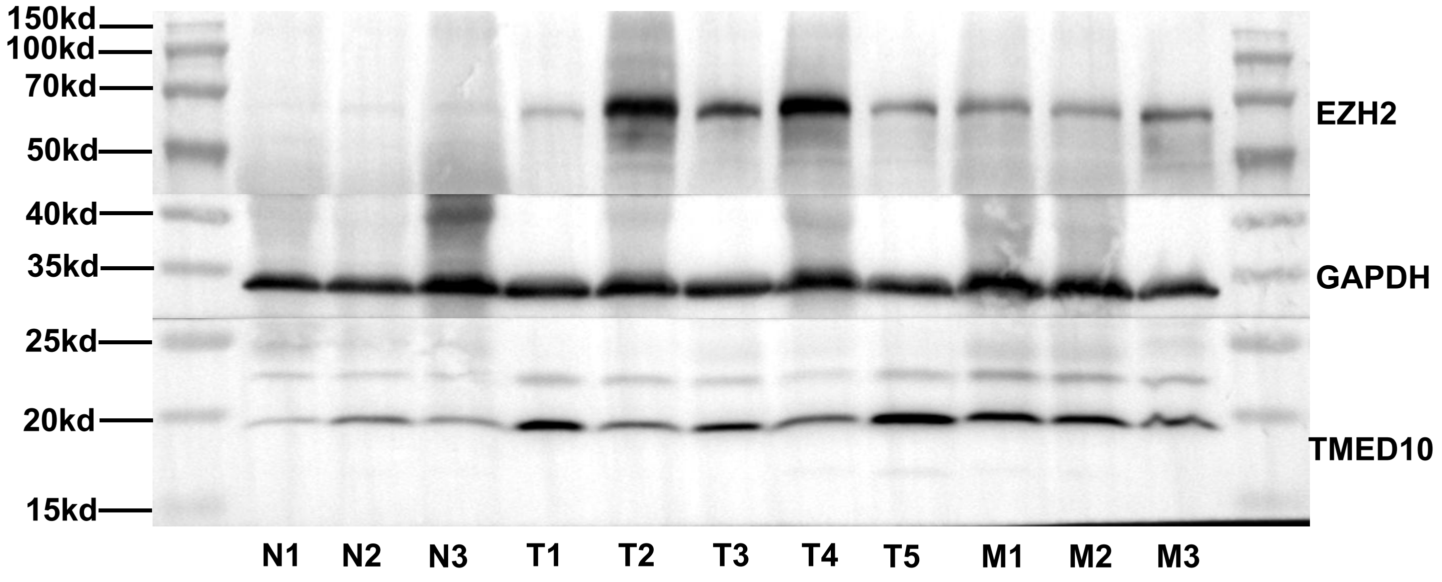
150kd
100kd
70kd
50kd
40kd
35kd
25kd
20kd
15kd
EZH2
GAPDH
TMED10
N1
N2
N3
T1
T2
T3
T4
T5
M1
M2
M3

Fig.2 A

Fig.2C

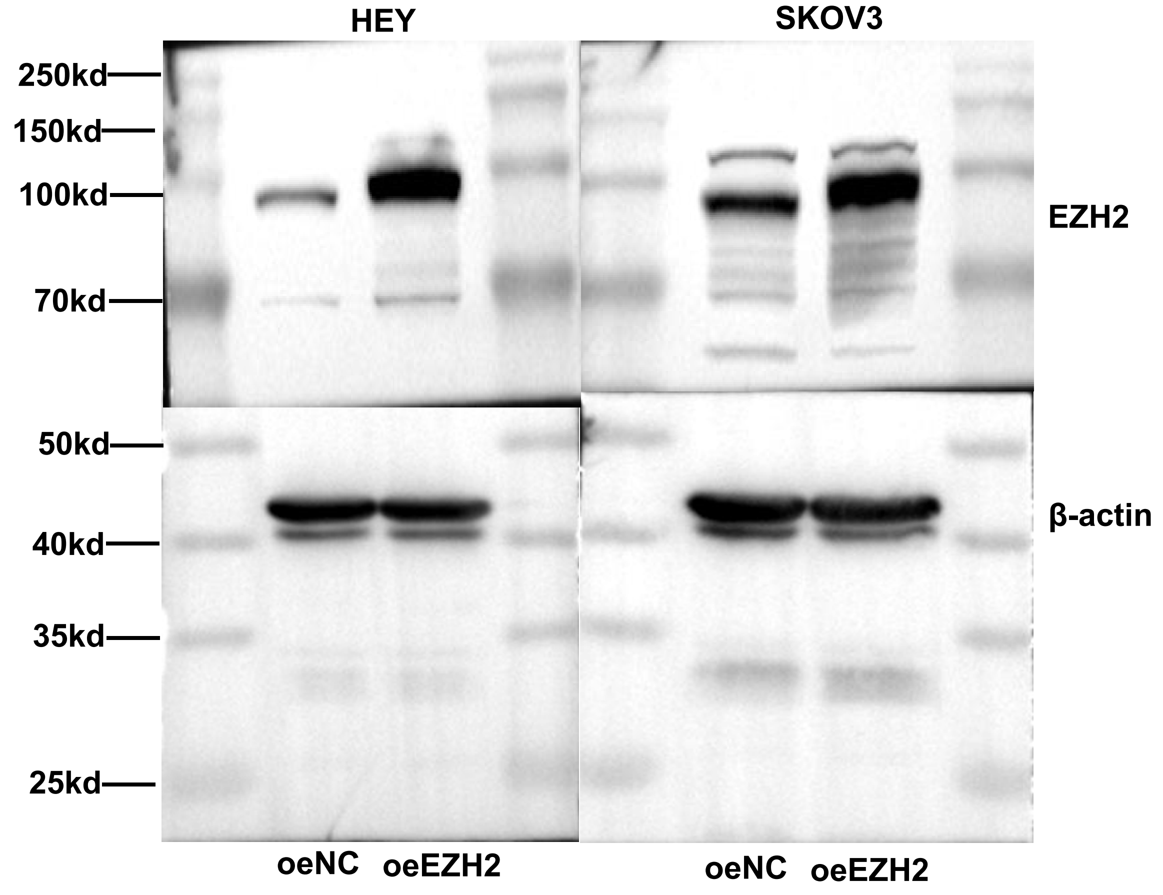

Fig.2 E

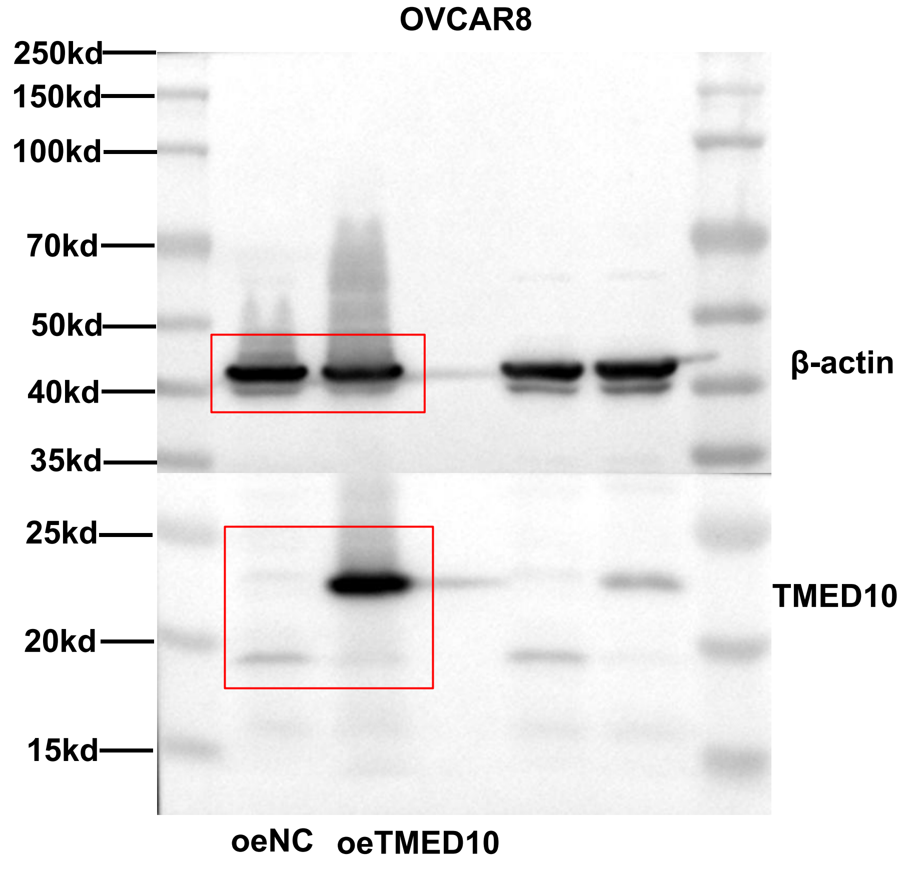

Fig.2 I

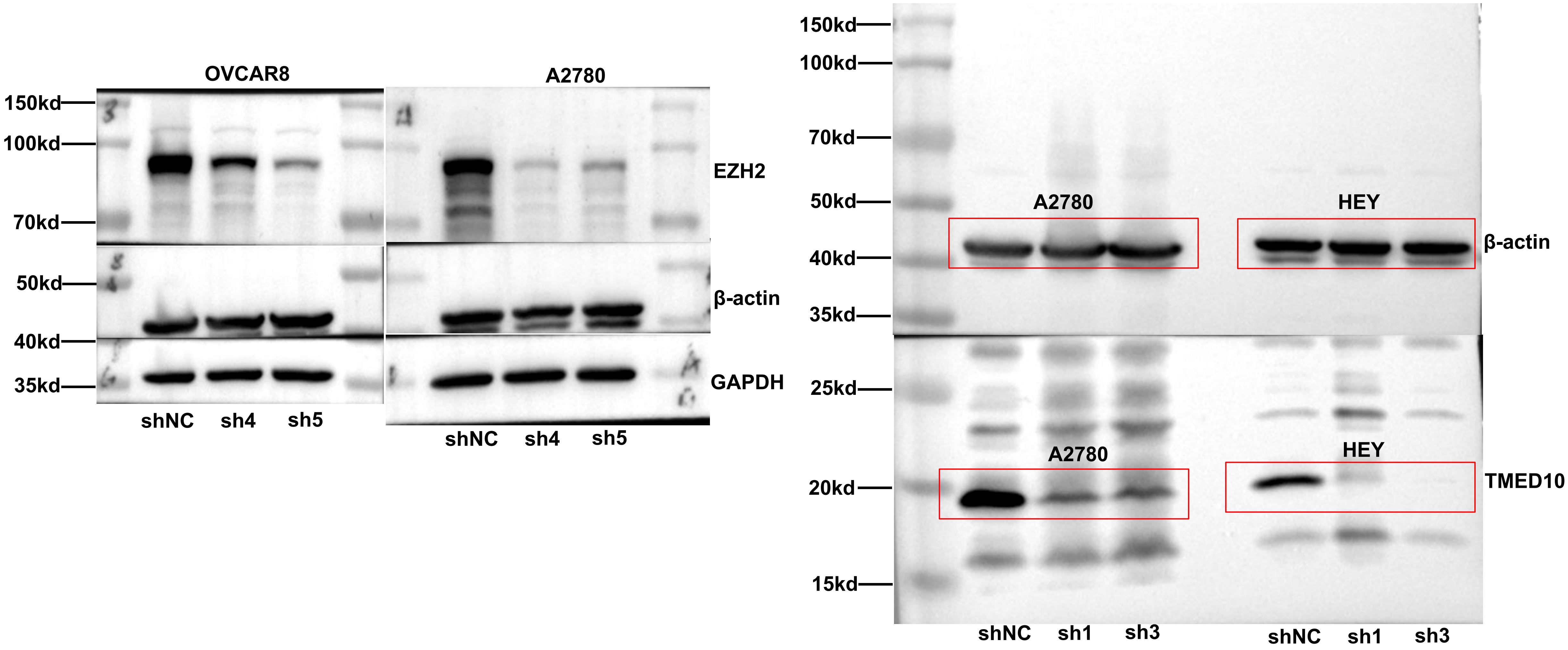

OVCAR8
A2780
150kd
100kd
70kd
50kd
40kd
35kd
EZH2
β-actin
GAPDH
shNC
sh4
sh5
shNC
sh4
sh5
150kd
100kd
70kd
50kd
40kd
35kd
25kd
20kd
15kd
A2780
HEY
β-actin
A2780
HEY
TMED10
shNC
sh1
sh3
shNC
sh1
sh3

Fig.3 C

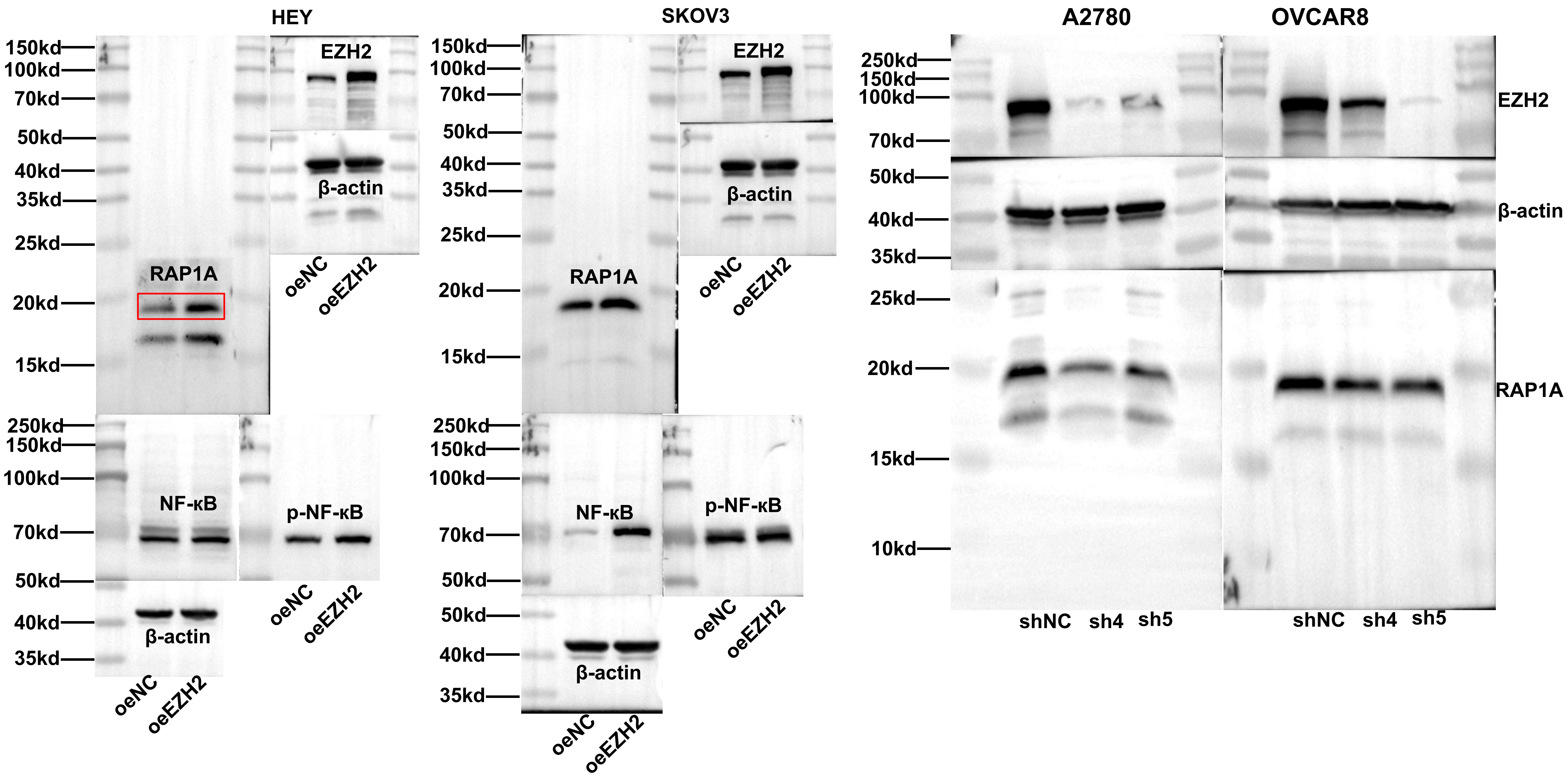
HEY
SKOV3
A2780
OVCAR8
150kd
100kd
70kd
50kd
40kd
35kd
25kd
20kd
15kd
250kd
10kd
EZH2
β-actin
RAP1A
NF-κB
p-NF-κB
oeNC
oeEZH2
shNC
sh4
sh5

Fig.3 D

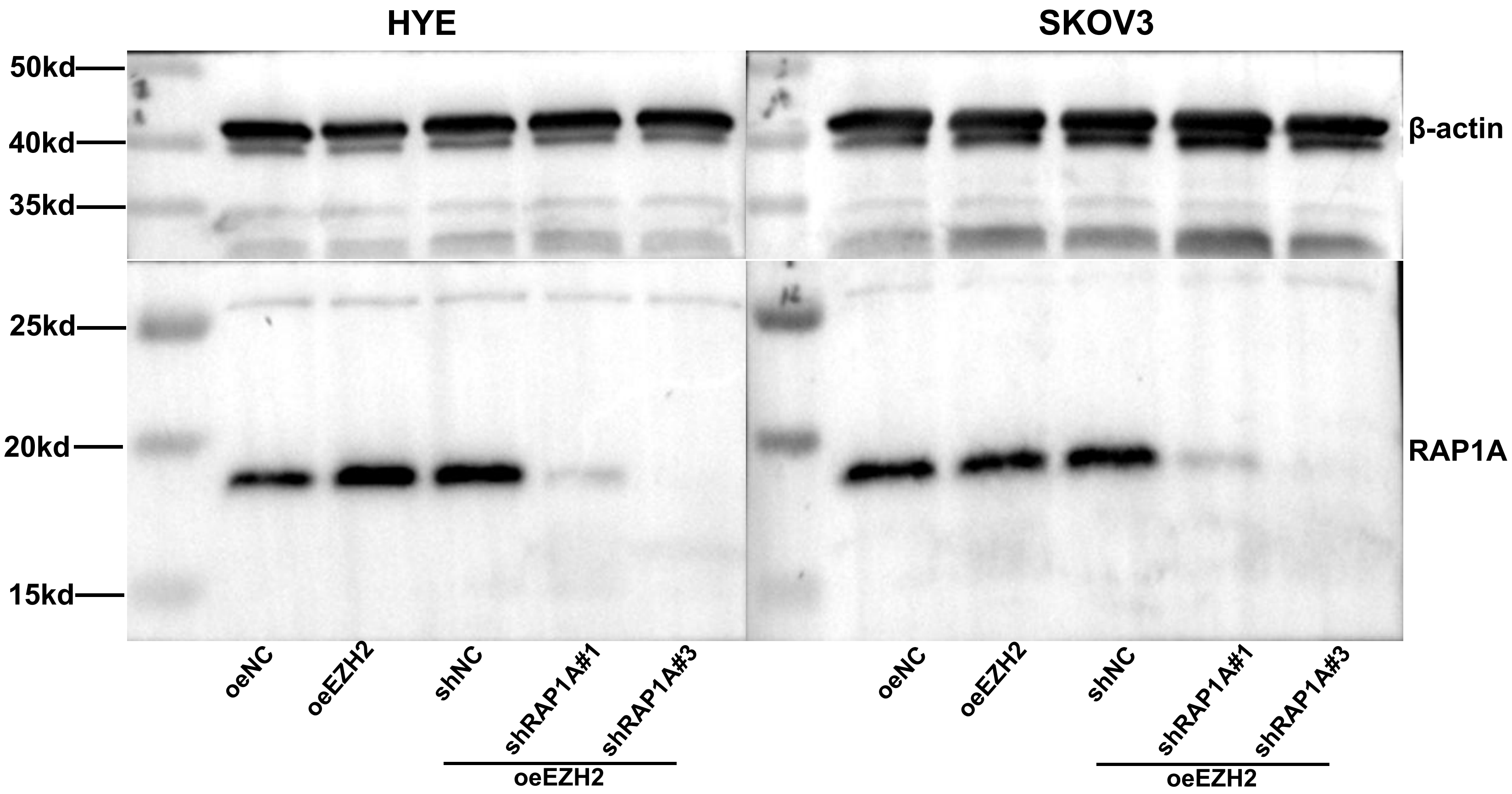
HYE
SKOV3
50kd
40kd
35kd
25kd
20kd
15kd
β-actin
RAP1A
oeNC
oeEZH2
shNC
shRAP1A#1
shRAP1A#3
oeEZH2
oeNC
oeEZH2
shNC
shRAP1A#1
shRAP1A#3
oeEZH2

Fig.4 K

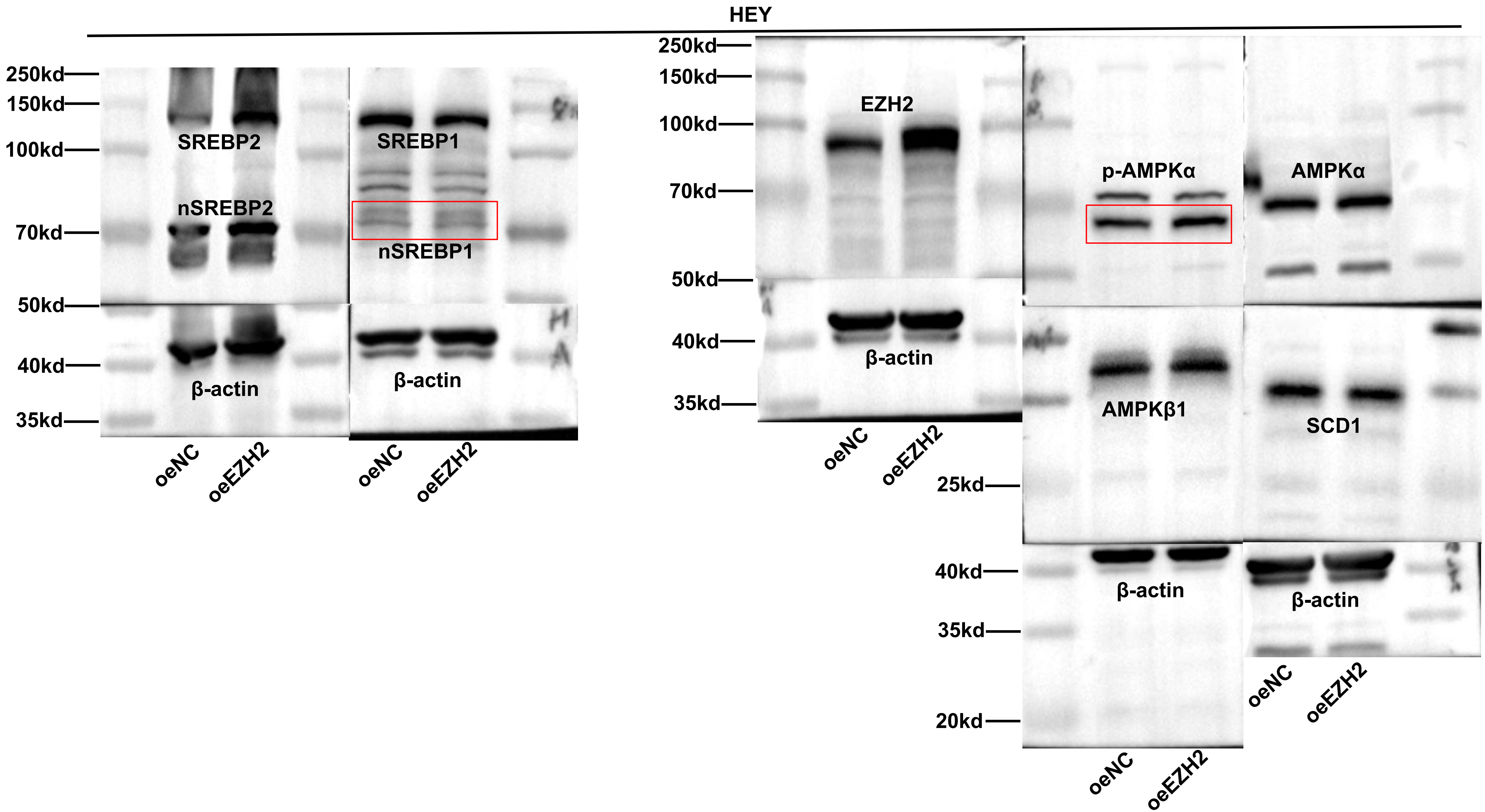
HEY
250kd
150kd
100kd
70kd
50kd
40kd
35kd
SREBP2
nSREBP2
β-actin
SREBP1
nSREBP1
β-actin
oeNC
oeEZH2
oeNC
oeEZH2
250kd
150kd
100kd
70kd
50kd
40kd
35kd
EZH2
β-actin
oeNC
oeEZH2
p-AMPKα
AMPKα
AMPKβ1
SCD1
25kd
40kd
35kd
20kd
β-actin
β-actin
oeNC
oeEZH2
oeNC
oeEZH2

Fig.4 K

SKOV3

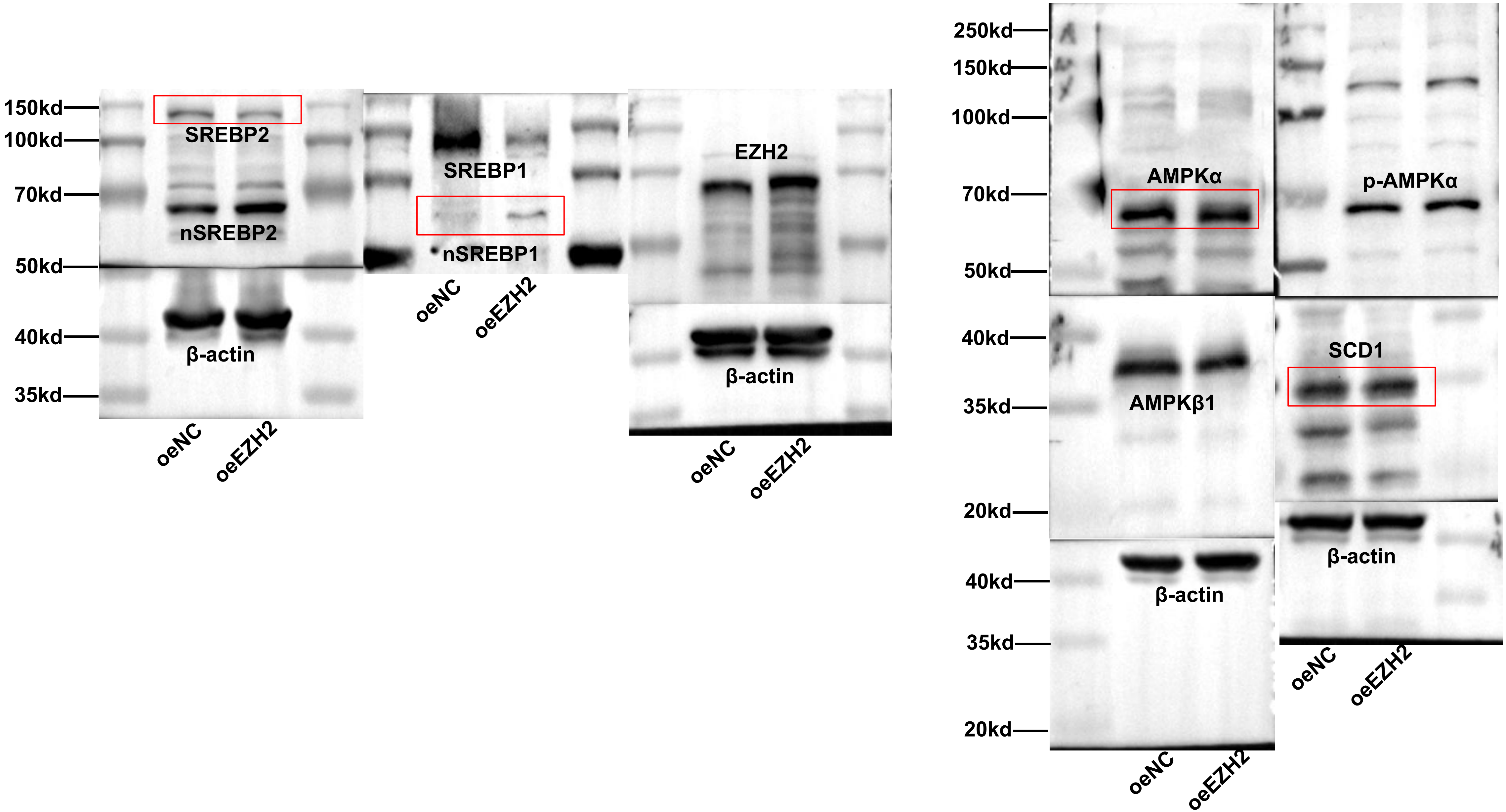

150kd
100kd
70kd
50kd
40kd
35kd
SREBP2
nSREBP2
β-actin
oeNC
oeEZH2
SREBP1
nSREBP1
oeNC
oeEZH2
EZH2
β-actin
oeNC
oeEZH2
250kd
150kd
100kd
70kd
50kd
40kd
35kd
20kd
AMPKα
p-AMPKα
AMPKβ1
SCD1
β-actin
40kd
35kd
20kd
β-actin
oeNC
oeEZH2
oeNC
oeEZH2

Fig.4 L

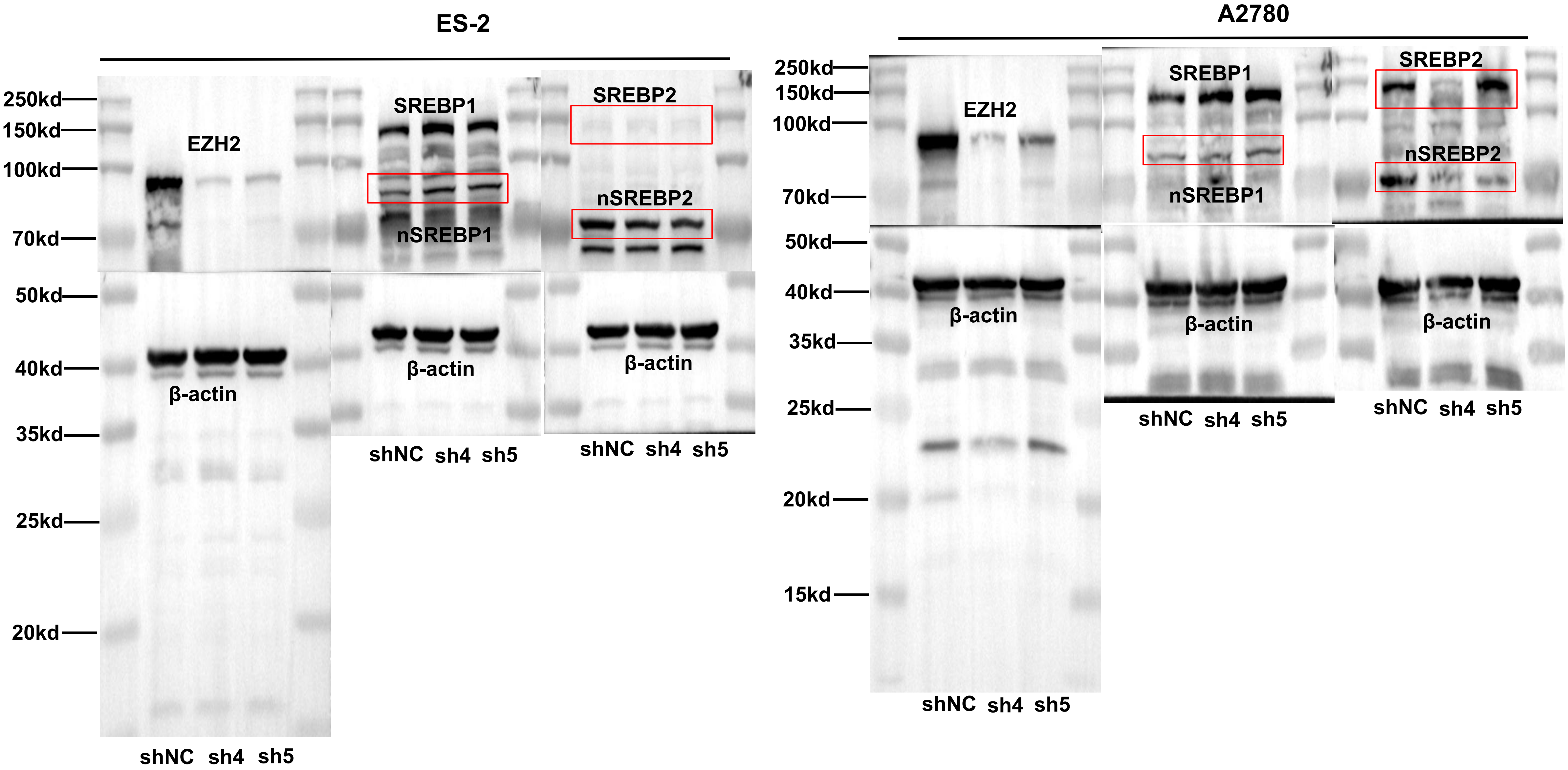
ES-2
A2780
250kd
150kd
100kd
70kd
50kd
40kd
35kd
25kd
20kd
15kd
EZH2
SREBP1
nSREBP1
SREBP2
nSREBP2
β-actin
shNC sh4 sh5

Fig.4 L

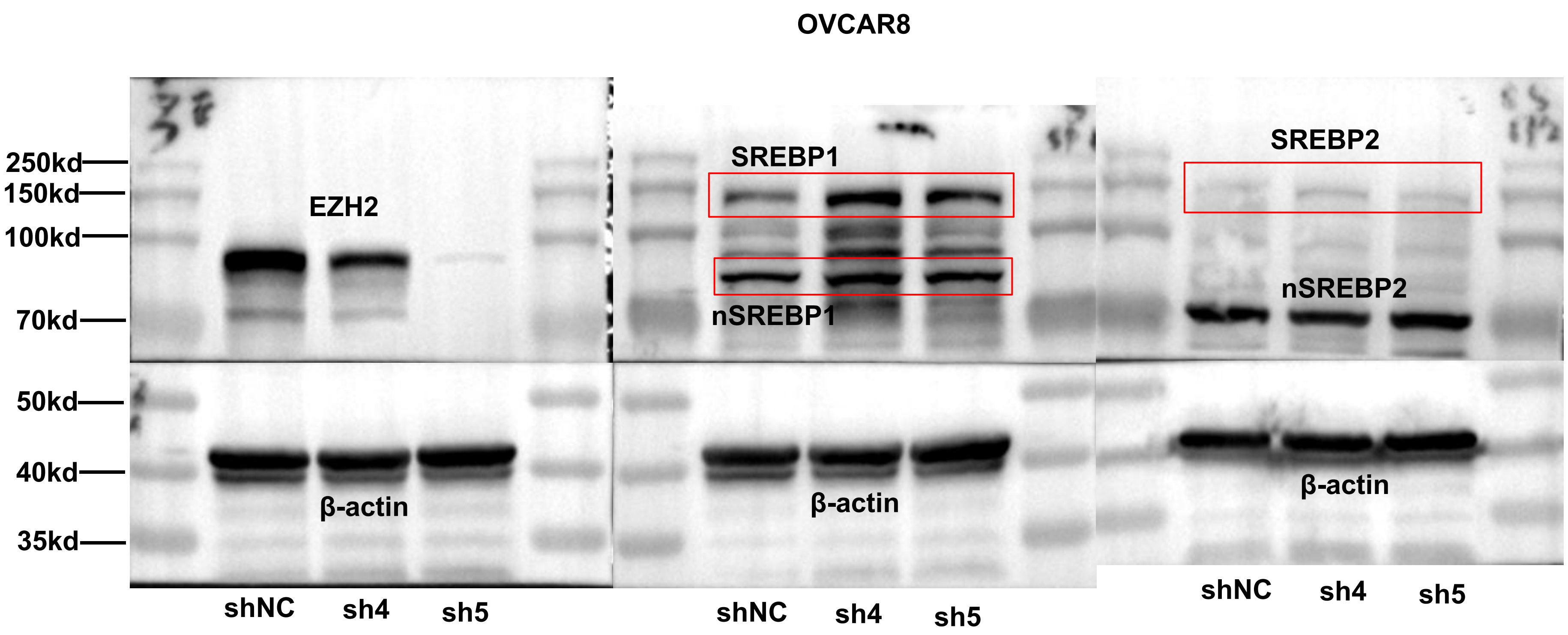

OVCAR8
250kd
150kd
100kd
70kd
50kd
40kd
35kd
EZH2
SREBP1
nSREBP1
SREBP2
nSREBP2
β-actin
β-actin
β-actin
shNC
sh4
sh5
shNC
sh4
sh5
shNC
sh4
sh5

Fig.5 A

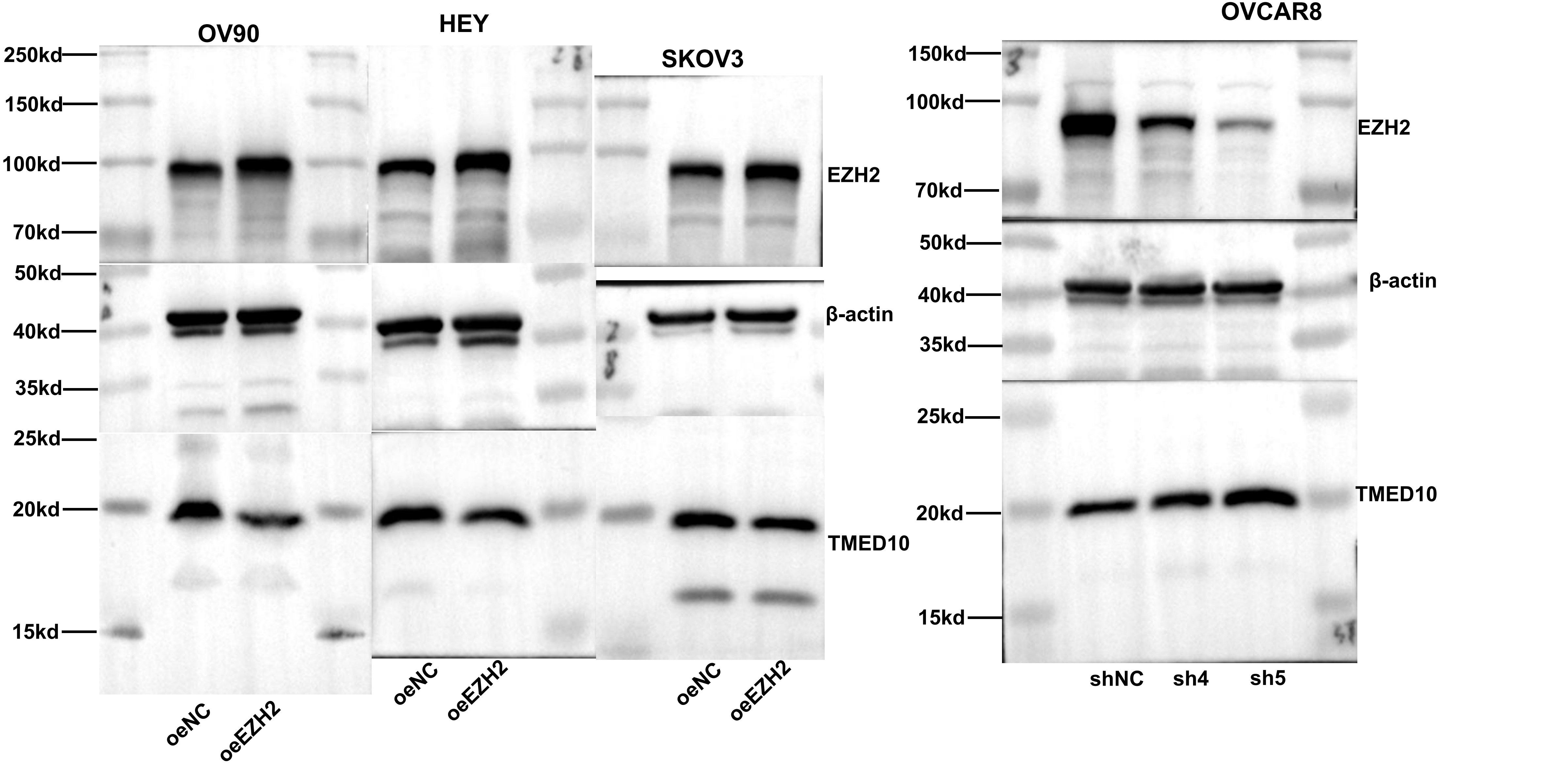
OV90
HEY
SKOV3
OVCAR8
250kd
150kd
100kd
70kd
50kd
40kd
35kd
25kd
20kd
15kd
EZH2
β-actin
TMED10
oeNC
oeEZH2
oeNC
oeEZH2
oeNC
oeEZH2
150kd
100kd
70kd
50kd
40kd
35kd
25kd
20kd
15kd
EZH2
β-actin
TMED10
shNC
sh4
sh5

Fig.5 G

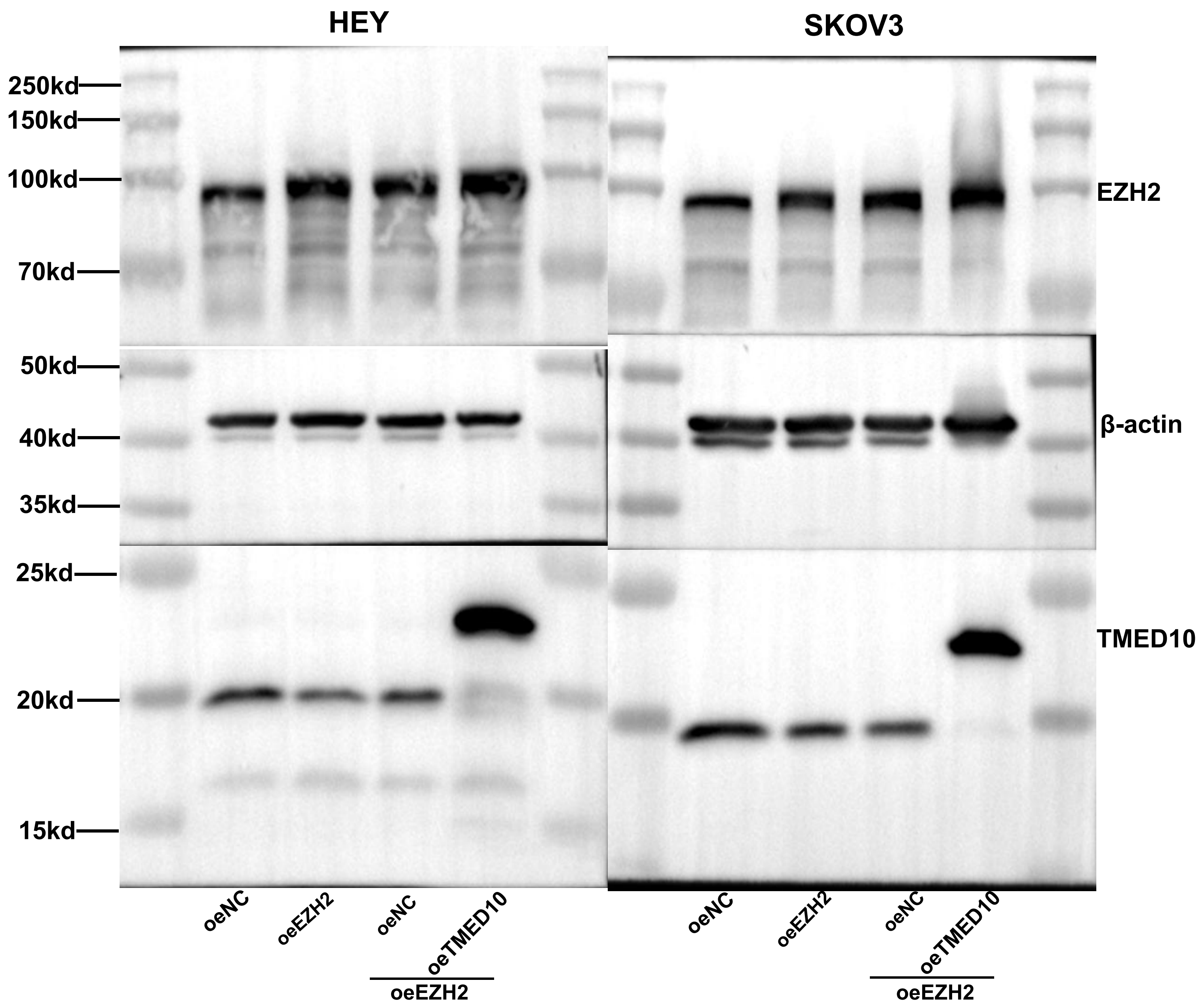
HEY
SKOV3
250kd
150kd
100kd
70kd
50kd
40kd
35kd
25kd
20kd
15kd
EZH2
β-actin
TMED10
oeNC
oeEZH2
oeNC
oeTMED10
oeEZH2
oeNC
oeEZH2
oeNC
oeTMED10
oeEZH2

Fig.5 J

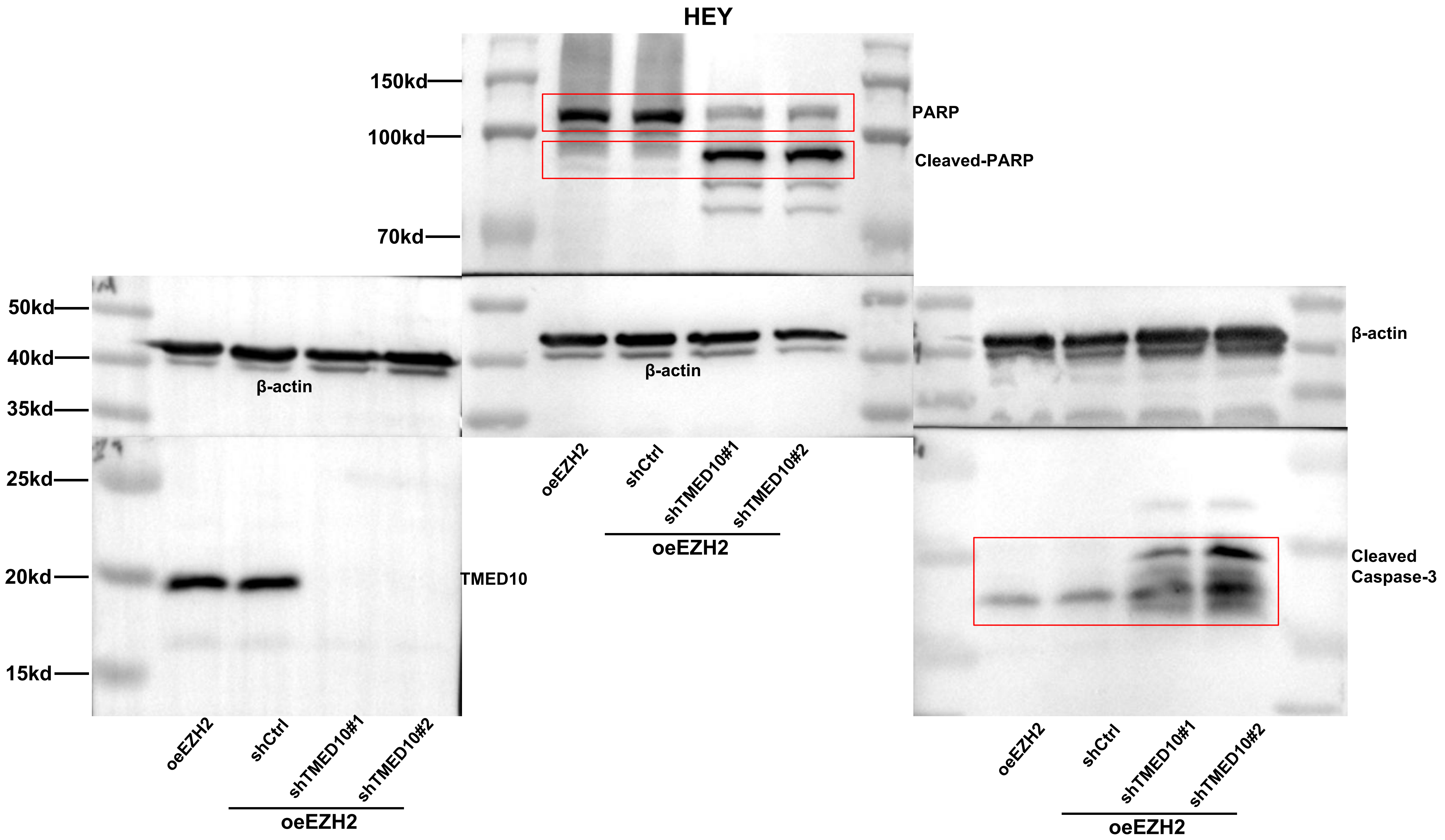
HEY
150kd
100kd
70kd
PARP
Cleaved-PARP
50kd
40kd
35kd
β-actin
β-actin
β-actin
25kd
20kd
15kd
TMED10
Cleaved Caspase-3
oeEZH2
shCtrl
shTMED10#1
shTMED10#2
oeEZH2

Fig.5 J

SKOV3

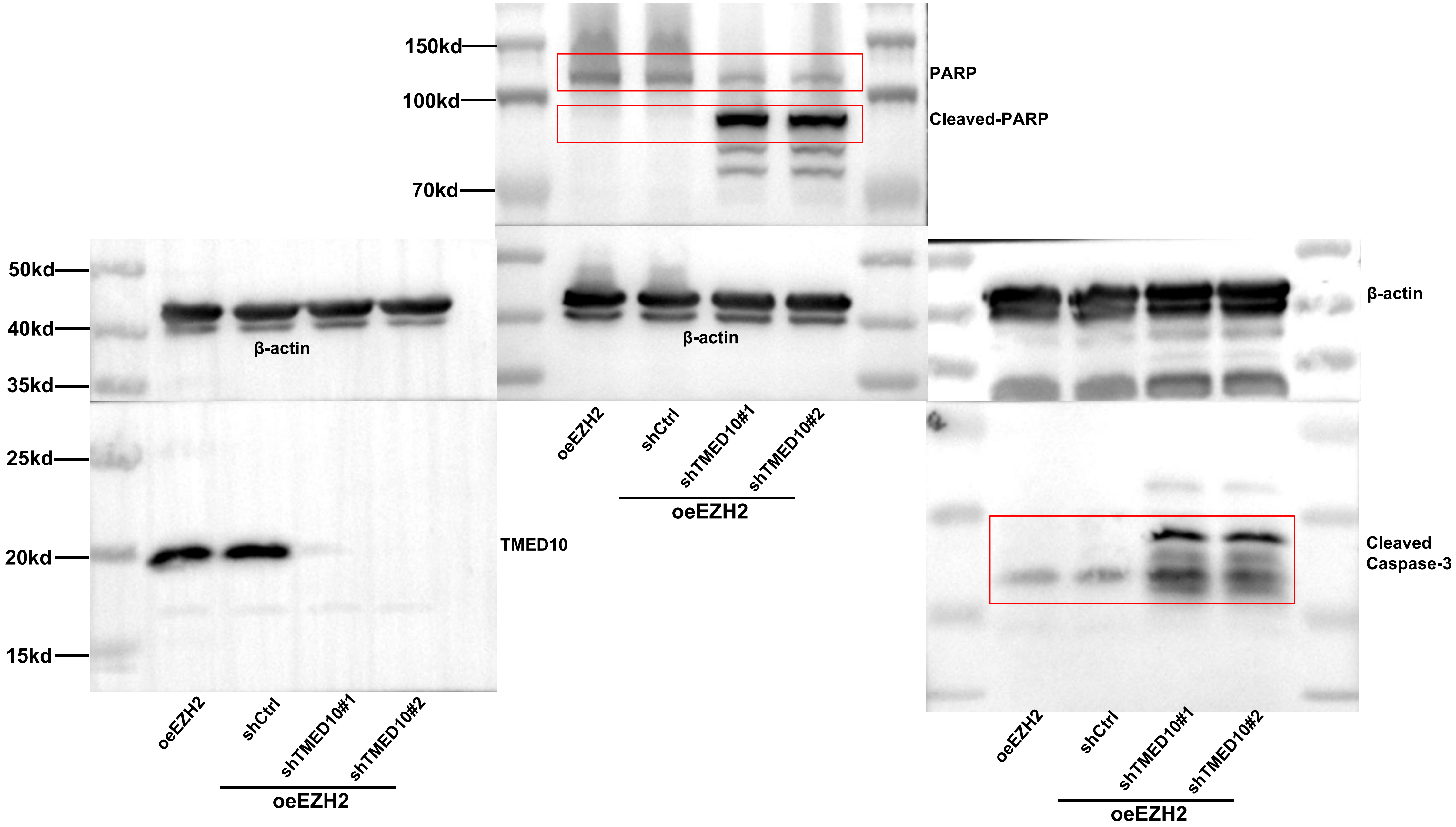
150kd
100kd
70kd
PARP
Cleaved-PARP
50kd
40kd
35kd
β-actin
β-actin
β-actin
25kd
20kd
15kd
TMED10
Cleaved
Caspase-3
oeEZH2
shCtrl
shTMED10#1
shTMED10#2
oeEZH2
oeEZH2
shCtrl
shTMED10#1
shTMED10#2
oeEZH2
oeEZH2
shCtrl
shTMED10#1
shTMED10#2
oeEZH2

Fig.6 E

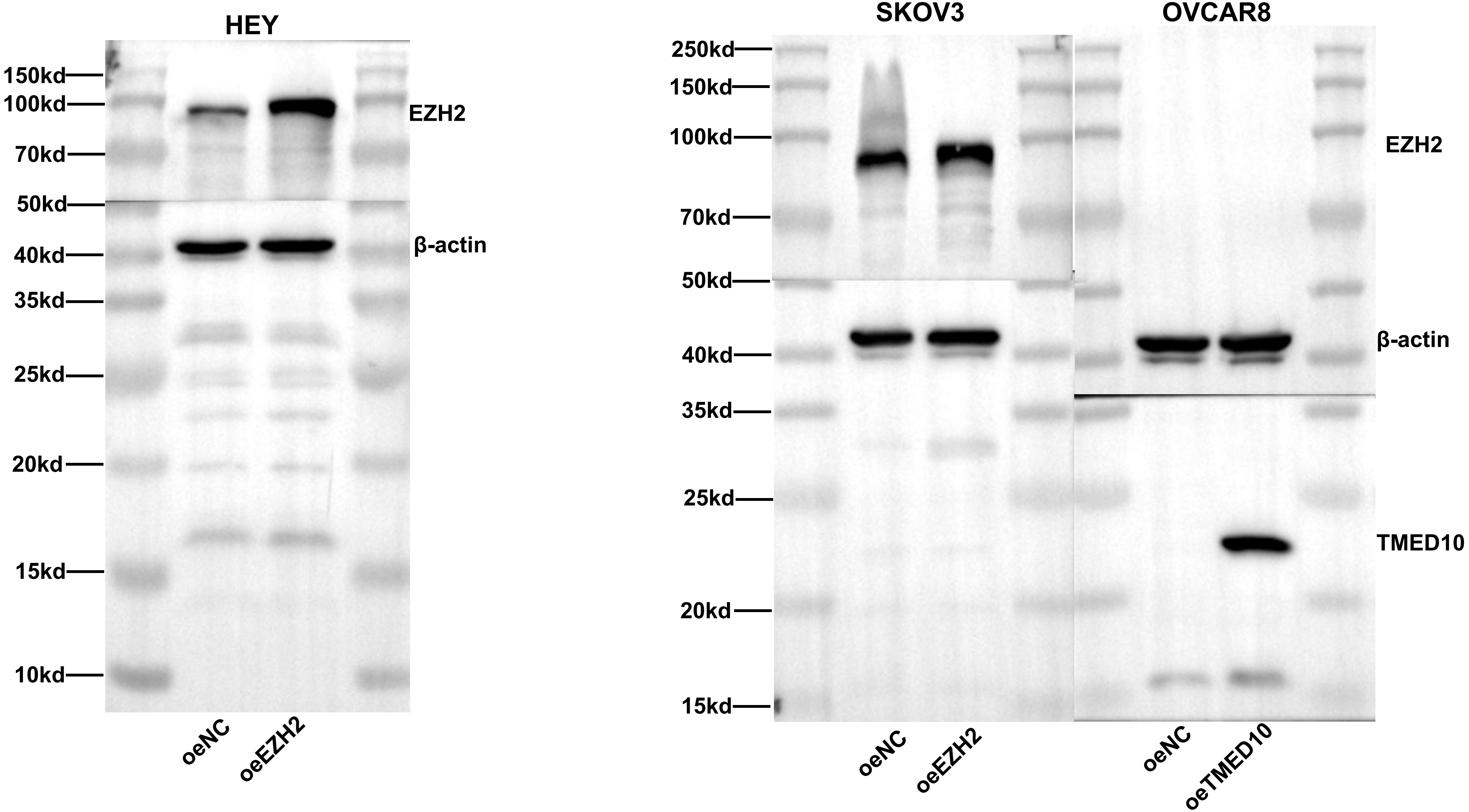
HEY
150kd
100kd
70kd
50kd
40kd
35kd
25kd
20kd
15kd
10kd
EZH2
β-actin
oeNC
oeEZH2
SKOV3
OVCAR8
250kd
150kd
100kd
70kd
50kd
40kd
35kd
25kd
20kd
15kd
EZH2
β-actin
TMED10
oeNC
oeEZH2
oeNC
oeTMED10

Fig.6 E

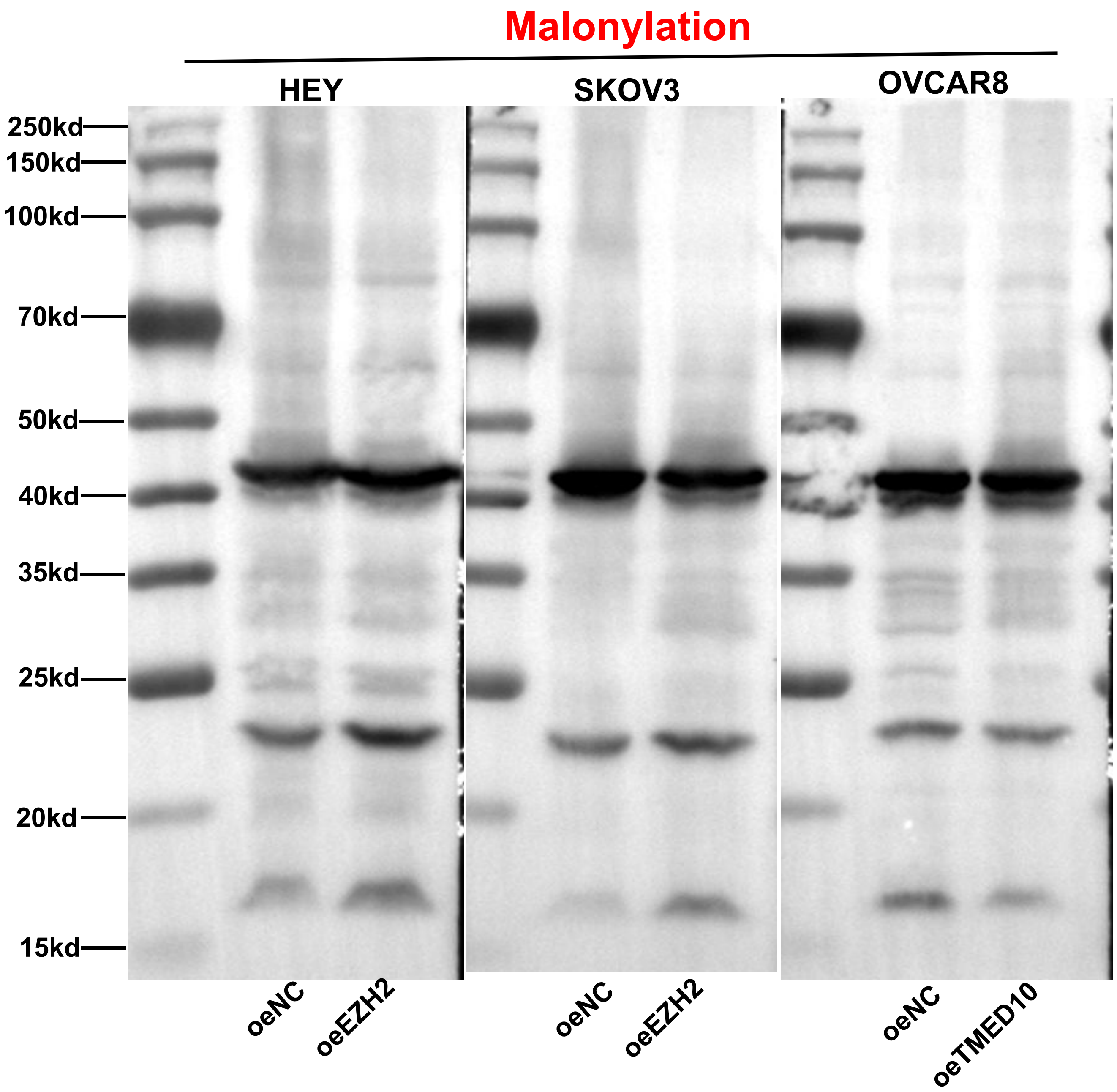
Malonylation
HEY
SKOV3
OVCAR8
250kd
150kd
100kd
70kd
50kd
40kd
35kd
25kd
20kd
15kd
oeNC
oeEZH2
oeNC
oeEZH2
oeNC
oeTMED10

Fig.7 E

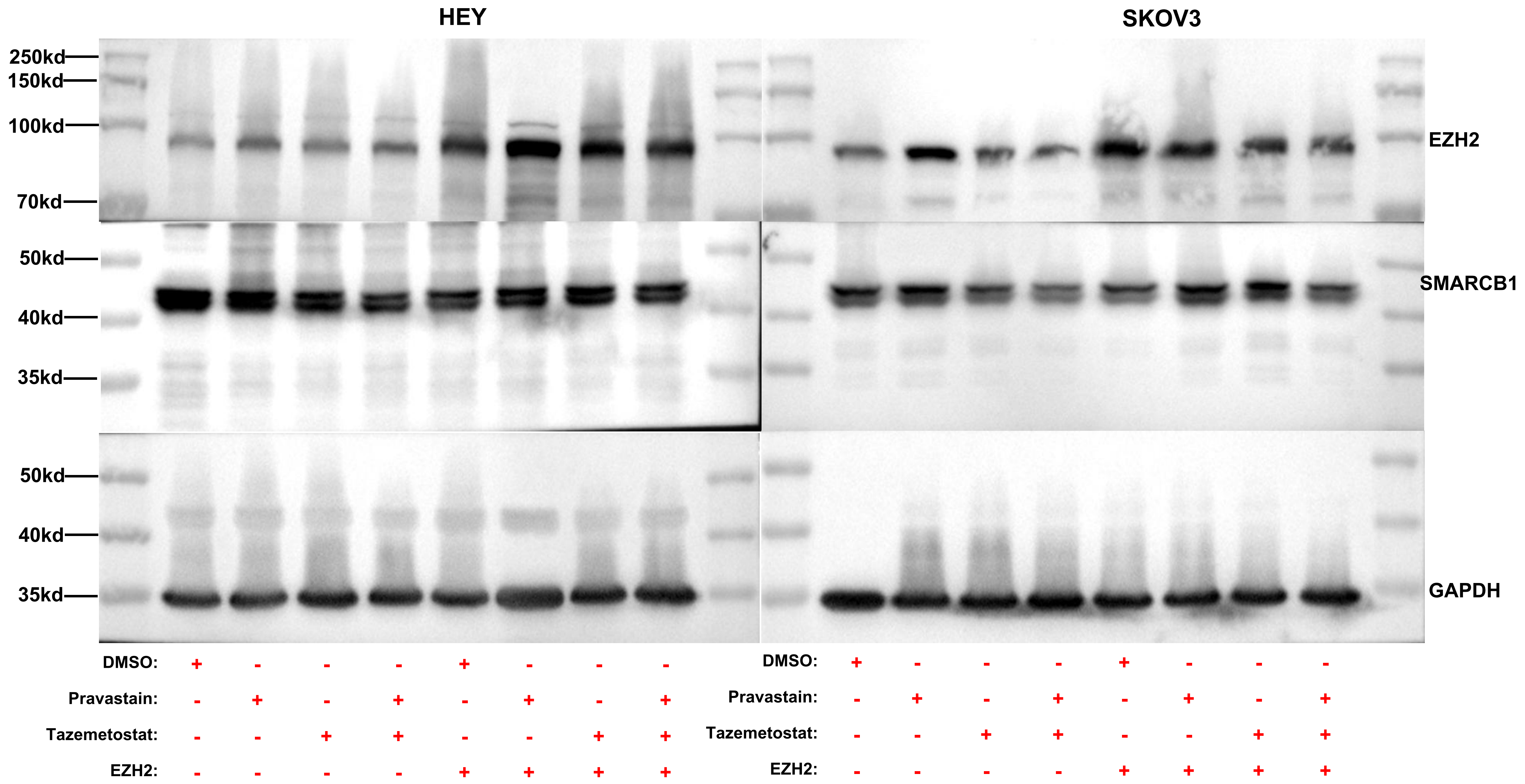

HEY
SKOV3
250kd
150kd
100kd
70kd
50kd
40kd
35kd
50kd
40kd
35kd
EZH2
SMARCB1
GAPDH
DMSO: + - - - + - - -
Pravastain: - + - + - + - +
Tazemetostat: - - + + - - + +
EZH2: - - - - + + + +
DMSO: + - - - + - - -
Pravastain: - + - + - + - +
Tazemetostat: - - + + - - + +
EZH2: - - - - + + + +

Fig.7 F

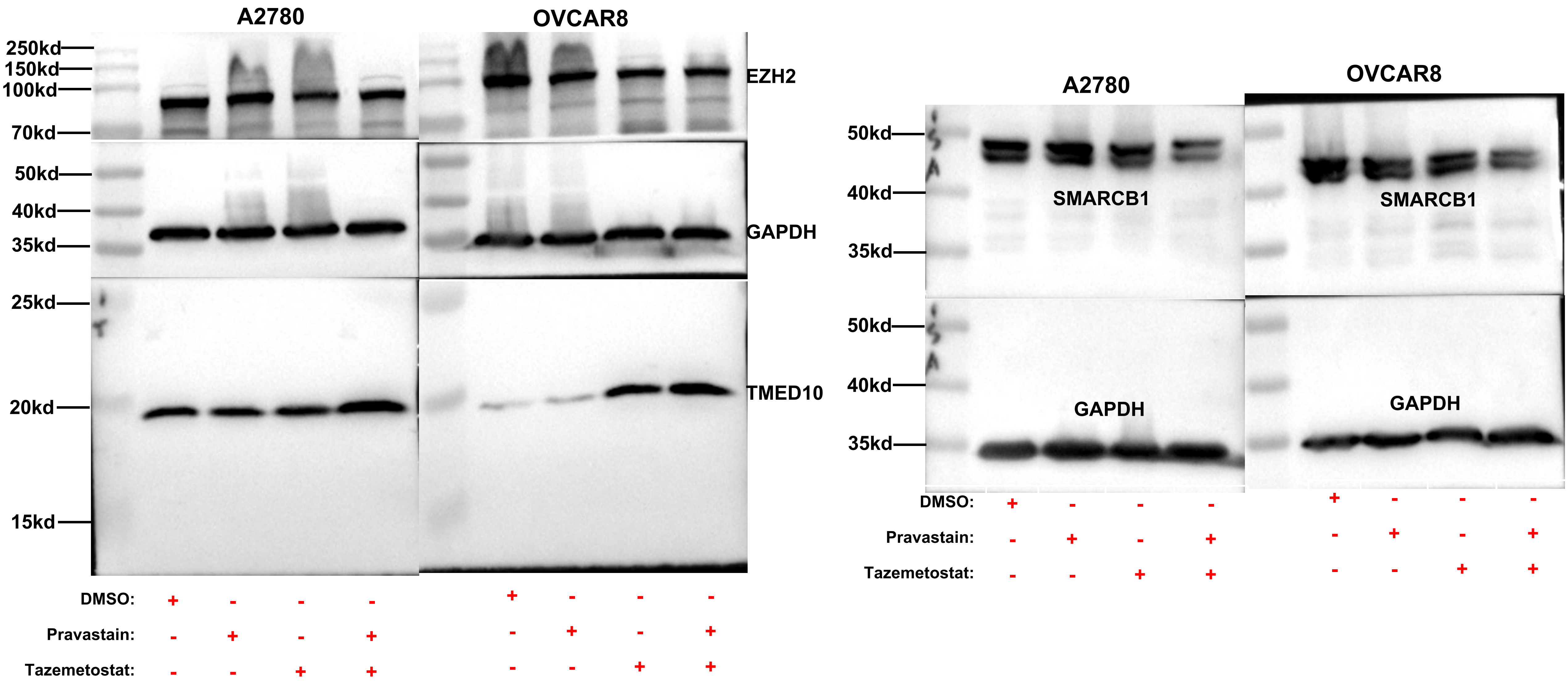
A2780
OVCAR8
250kd
150kd
100kd
70kd
50kd
40kd
35kd
25kd
20kd
15kd
EZH2
GAPDH
TMED10
DMSO: + - - - + - - -
Pravastain: - + - + - + - +
Tazemetostat: - - + + - - + +
A2780
OVCAR8
50kd
40kd
35kd
SMARCB1
SMARCB1
50kd
40kd
35kd
GAPDH
GAPDH
DMSO: + - - - + - - -
Pravastain: - + - + - + - +
Tazemetostat: - - + + - - + +

Fig.7 F

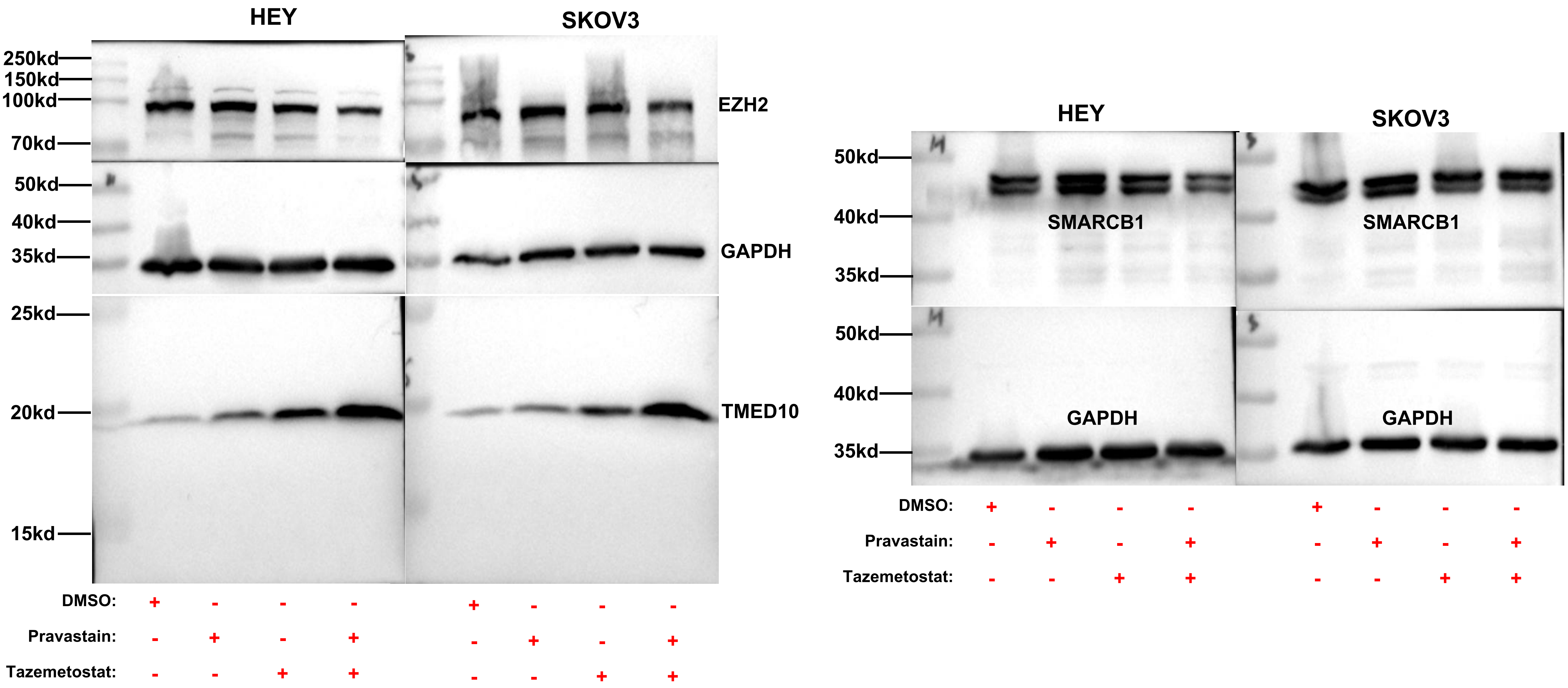
HEY
SKOV3
250kd
150kd
100kd
70kd
EZH2
50kd
40kd
35kd
GAPDH
25kd
20kd
TMED10
15kd
DMSO: + - - - + - - -
Pravastain: - + - + - + - +
Tazemetostat: - - + + - - + +
HEY
SKOV3
50kd
40kd
35kd
SMARCB1
SMARCB1
50kd
40kd
35kd
GAPDH
GAPDH
DMSO: + - - - + - - -
Pravastain: - + - + - + - +
Tazemetostat: - - + + - - + +

Fig.8 G

HEY

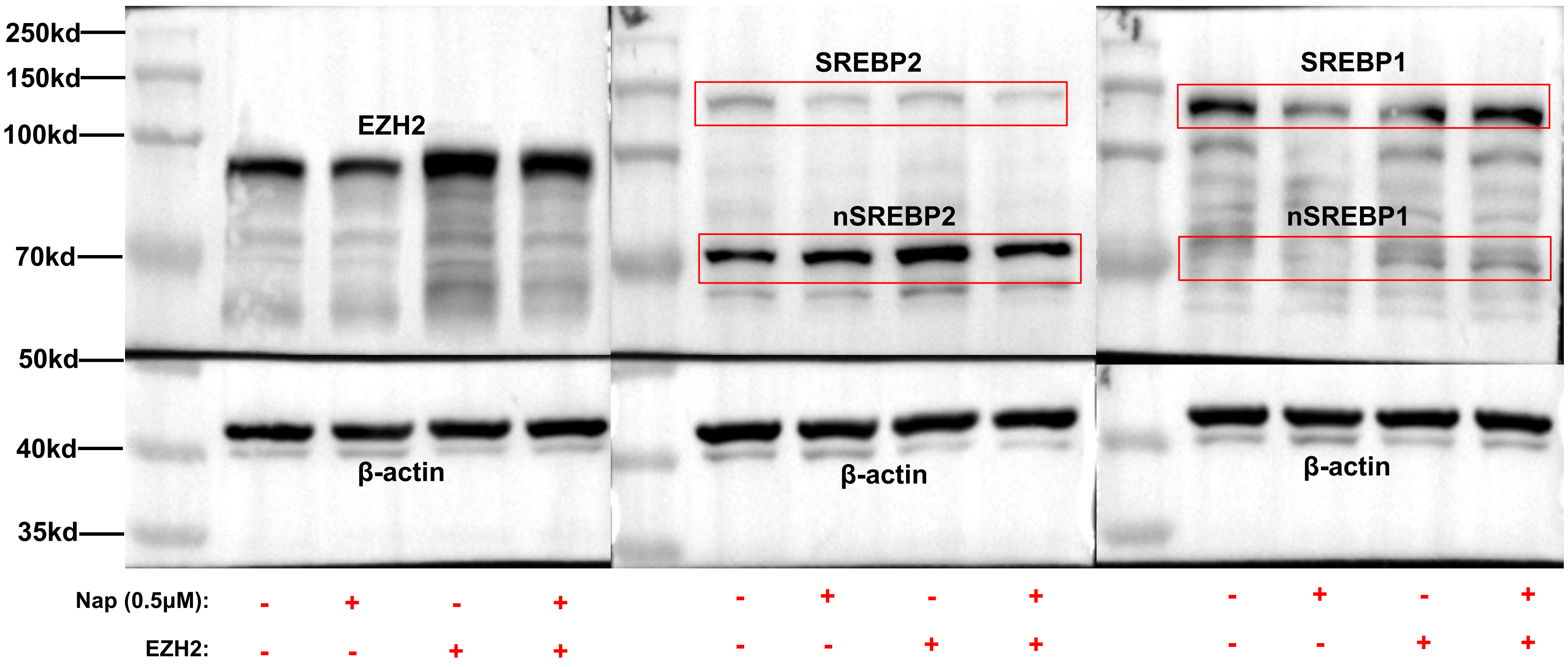
250kd
150kd
100kd
70kd
50kd
40kd
35kd
EZH2
β-actin
SREBP2
nSREBP2
β-actin
SREBP1
nSREBP1
β-actin
Nap (0.5μM): - + - + - + - + - + - +
EZH2: - - + + - - + + - - + +

Fig.8 H

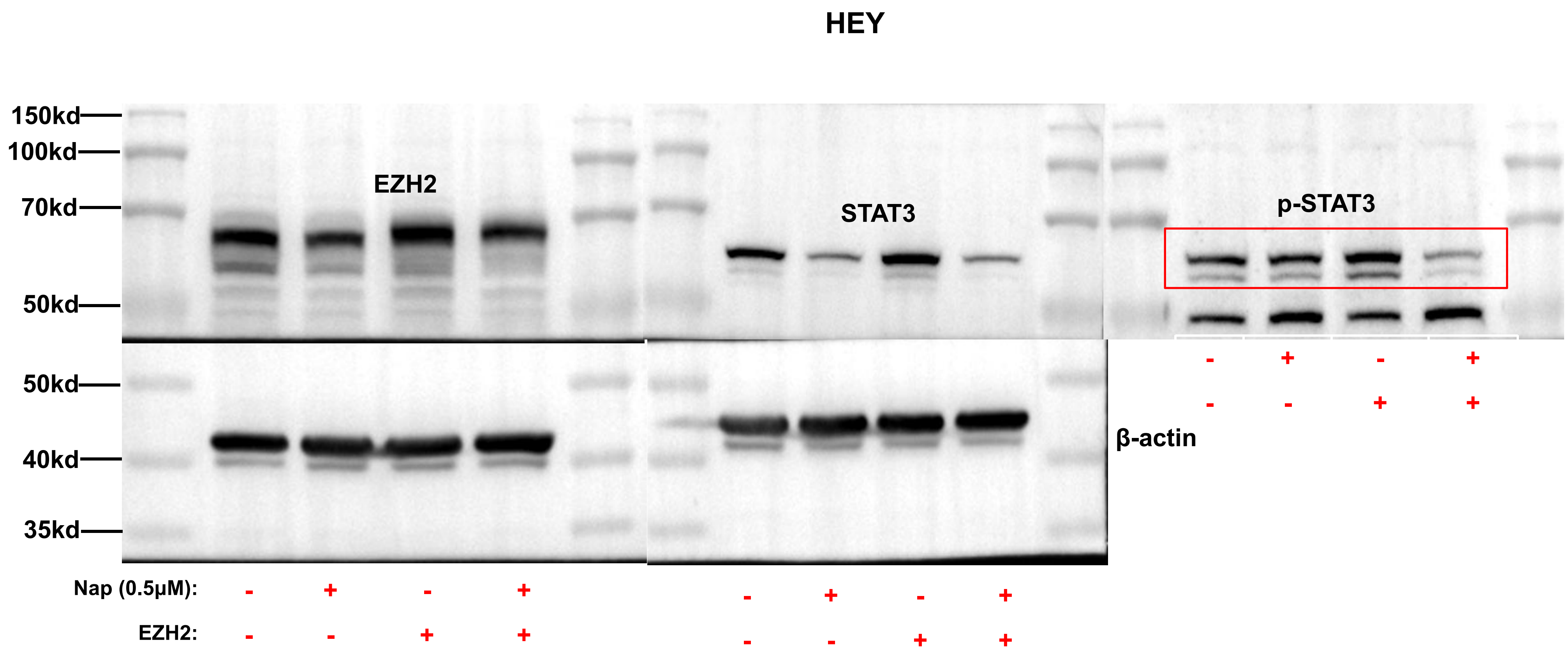
HEY
150kd
100kd
70kd
50kd
50kd
40kd
35kd
EZH2
STAT3
p-STAT3
β-actin
Nap (0.5μM): - + - +
EZH2: - - + +
- + - +
- - + +
- + - +
- - + +

Fig.8 H

HEY

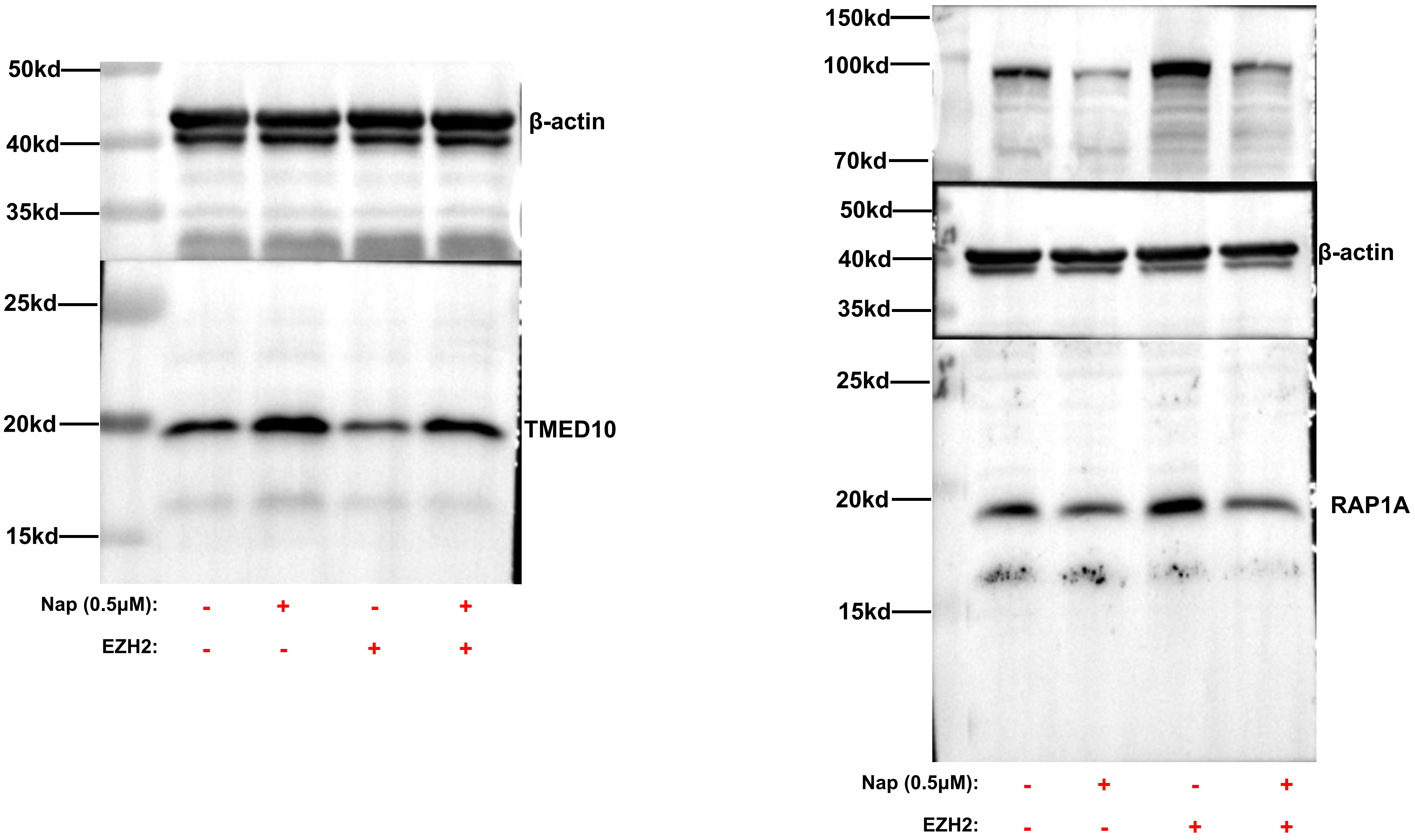

50kd
40kd
35kd
25kd
20kd
15kd
β-actin
TMED10
Nap (0.5μM): - + - +
EZH2: - - + +
150kd
100kd
70kd
50kd
40kd
35kd
25kd
20kd
15kd
β-actin
RAP1A
Nap (0.5μM): - + - +
EZH2: - - + +

Fig.8 H

SKOV3

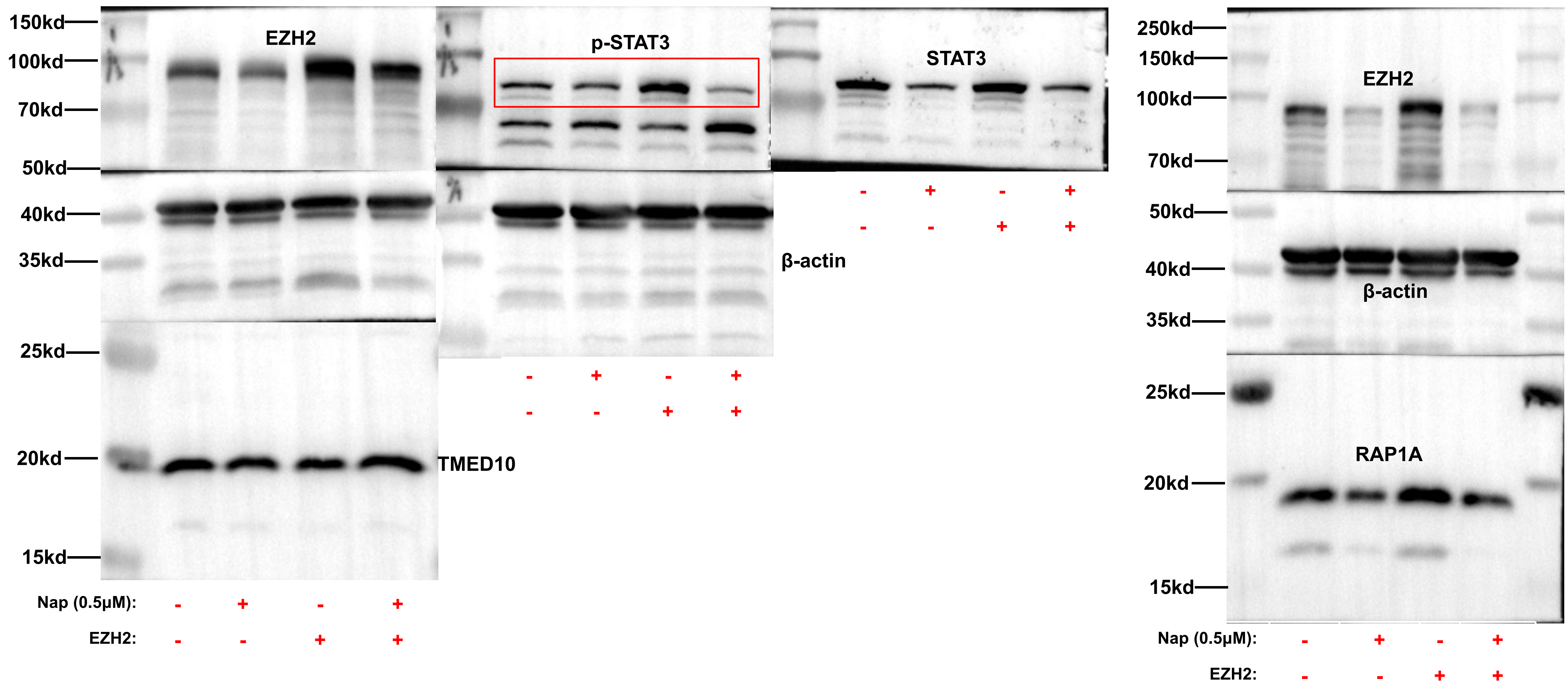
150kd
100kd
70kd
50kd
40kd
35kd
25kd
20kd
15kd
EZH2
p-STAT3
STAT3
β-actin
TMED10
250kd
150kd
100kd
70kd
50kd
40kd
35kd
25kd
20kd
15kd
EZH2
β-actin
RAP1A
- + - +
- - + +
- + - +
- - + +
Nap (0.5μM): - + - +
EZH2: - - + +
Nap (0.5μM): - + - +
EZH2: - - + +

Fig.8 I

HEY

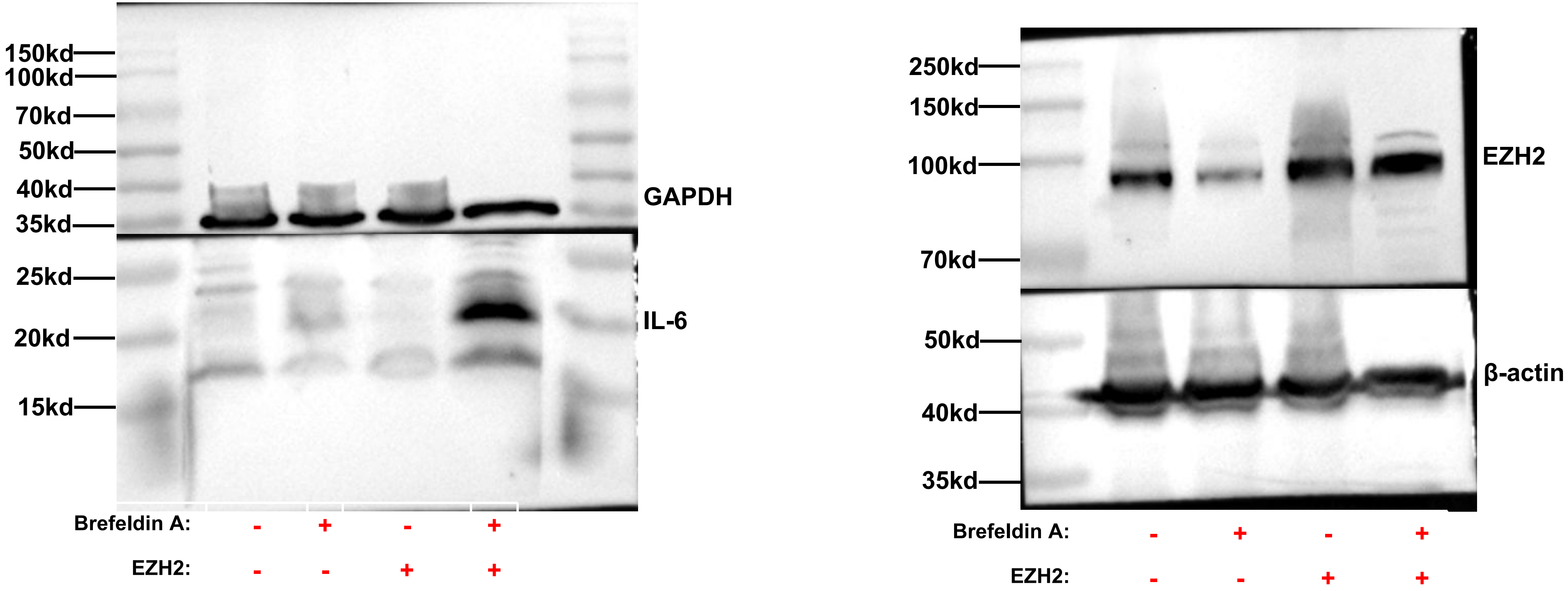
150kd
100kd
70kd
50kd
40kd
35kd
25kd
20kd
15kd
GAPDH
IL-6
Brefeldin A: - + - +
EZH2: - - + +
250kd
150kd
100kd
70kd
50kd
40kd
35kd
EZH2
β-actin
Brefeldin A: - + - +
EZH2: - - + +

Supplementary Fig.2B

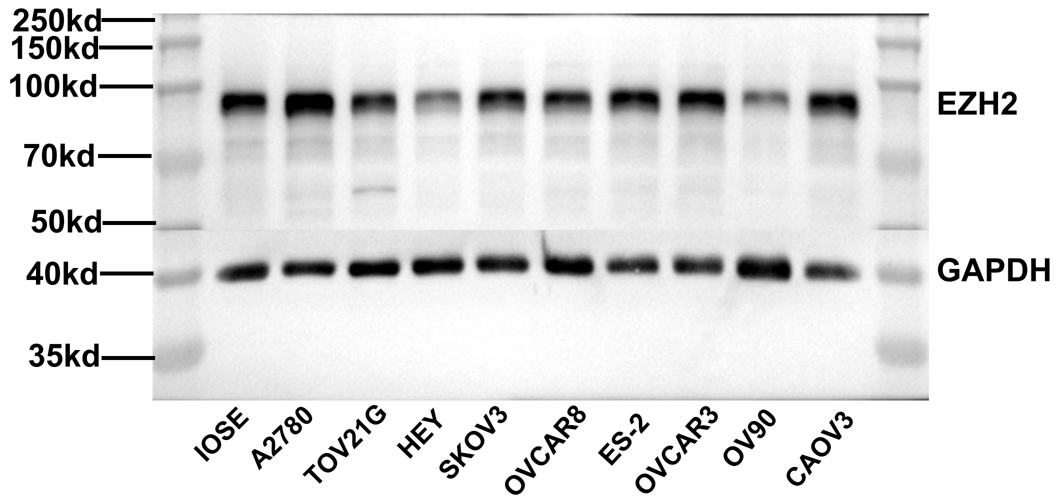

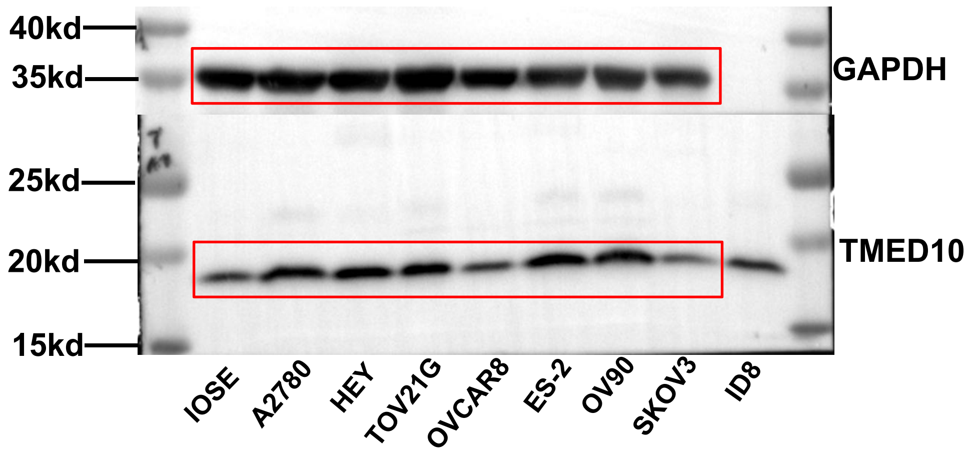

Supplementary Fig. 2C

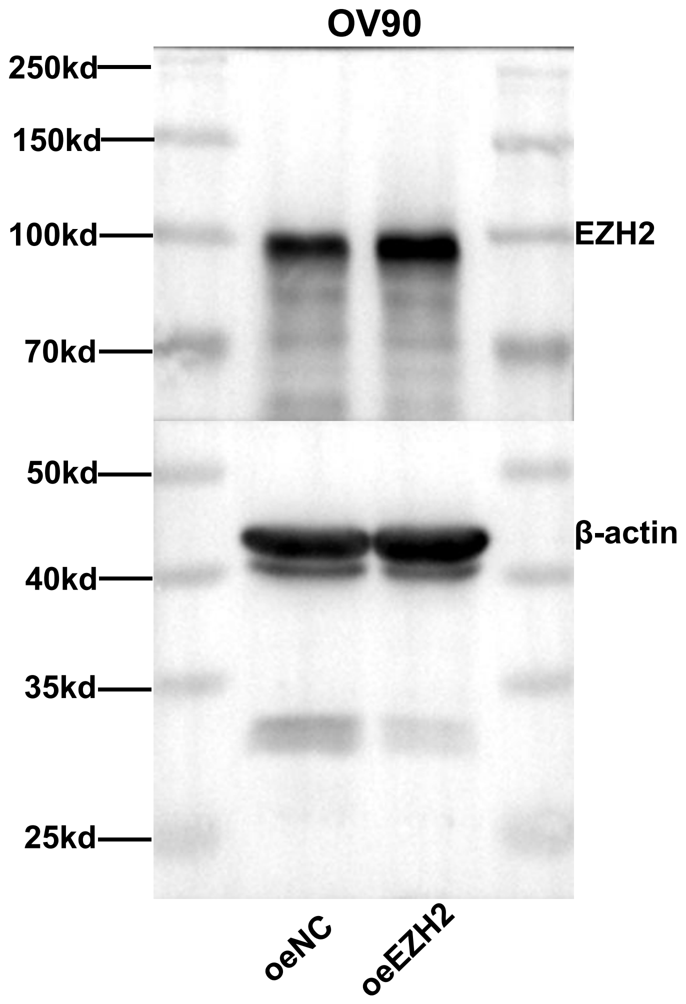

Supplementary Fig.2 F

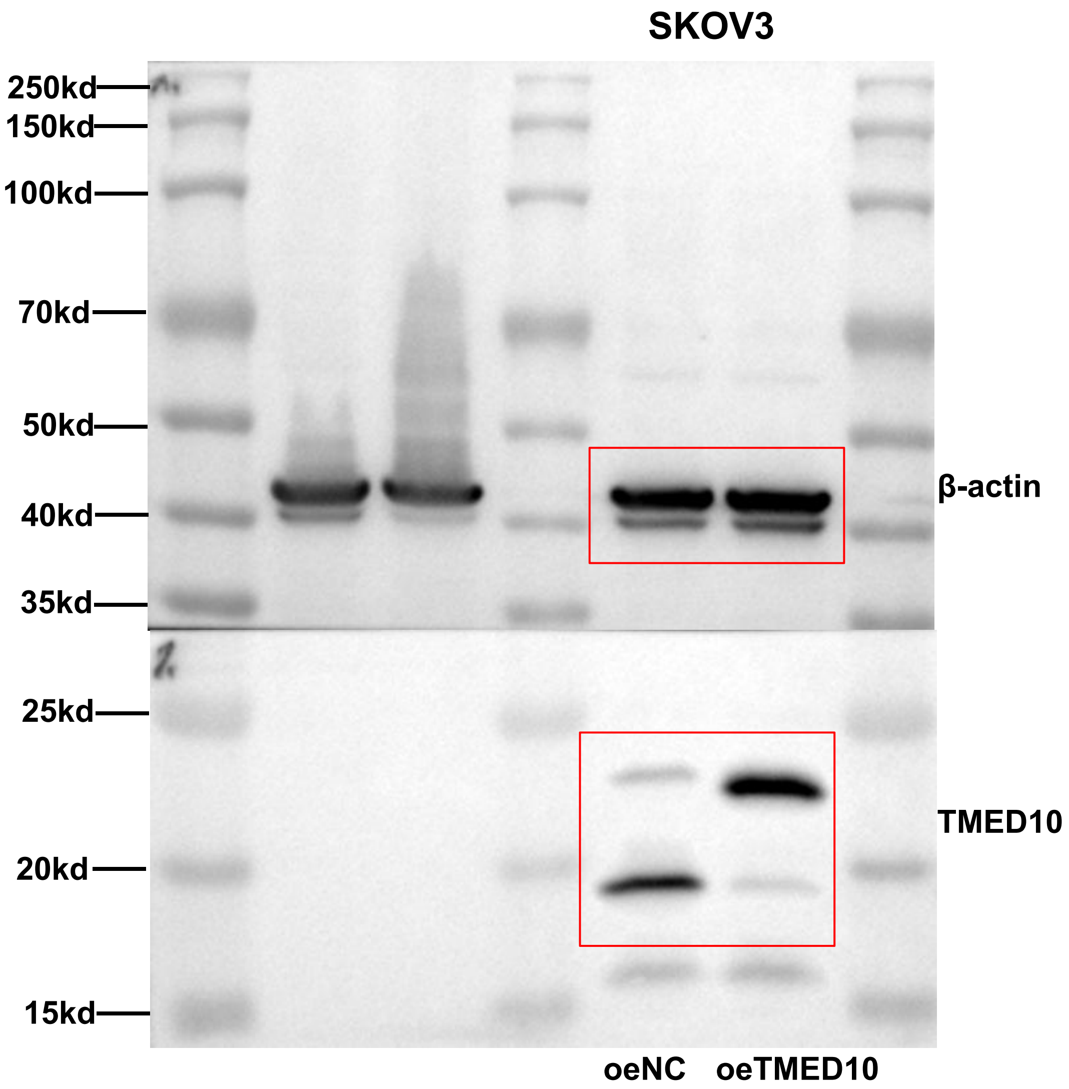

Supplementary Fig.2 J

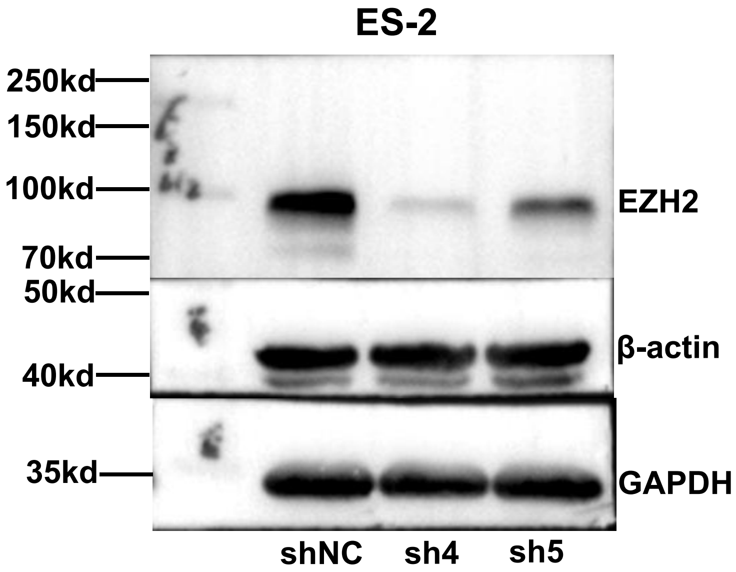

Supplementary Fig.2 O

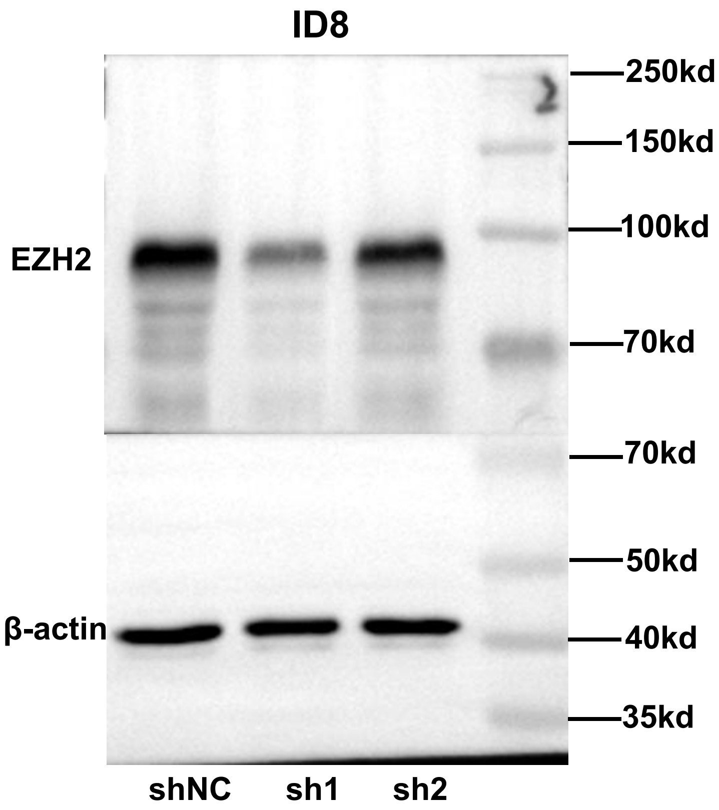

Supplementary Fig. 3C

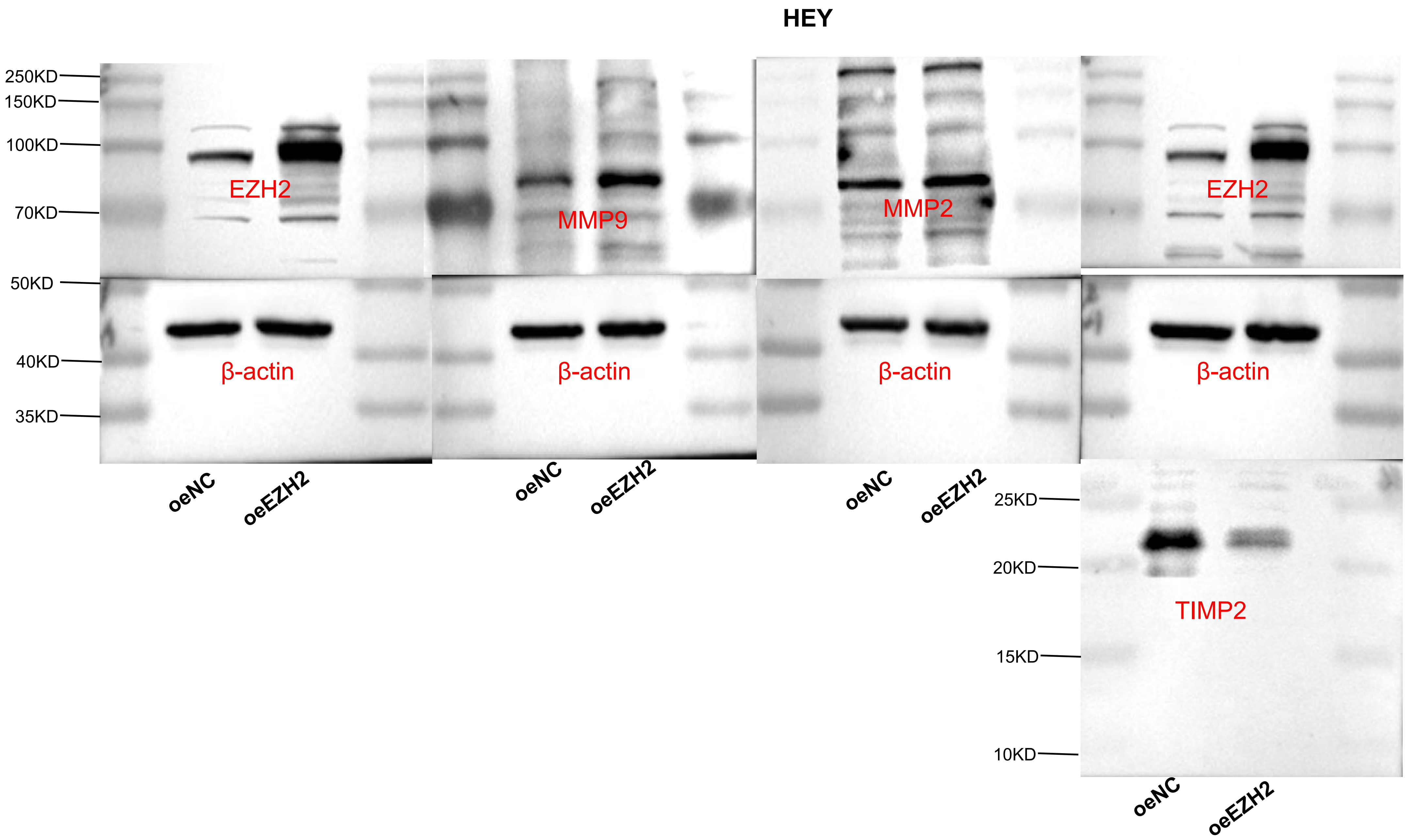

Supplementary Fig. 3C

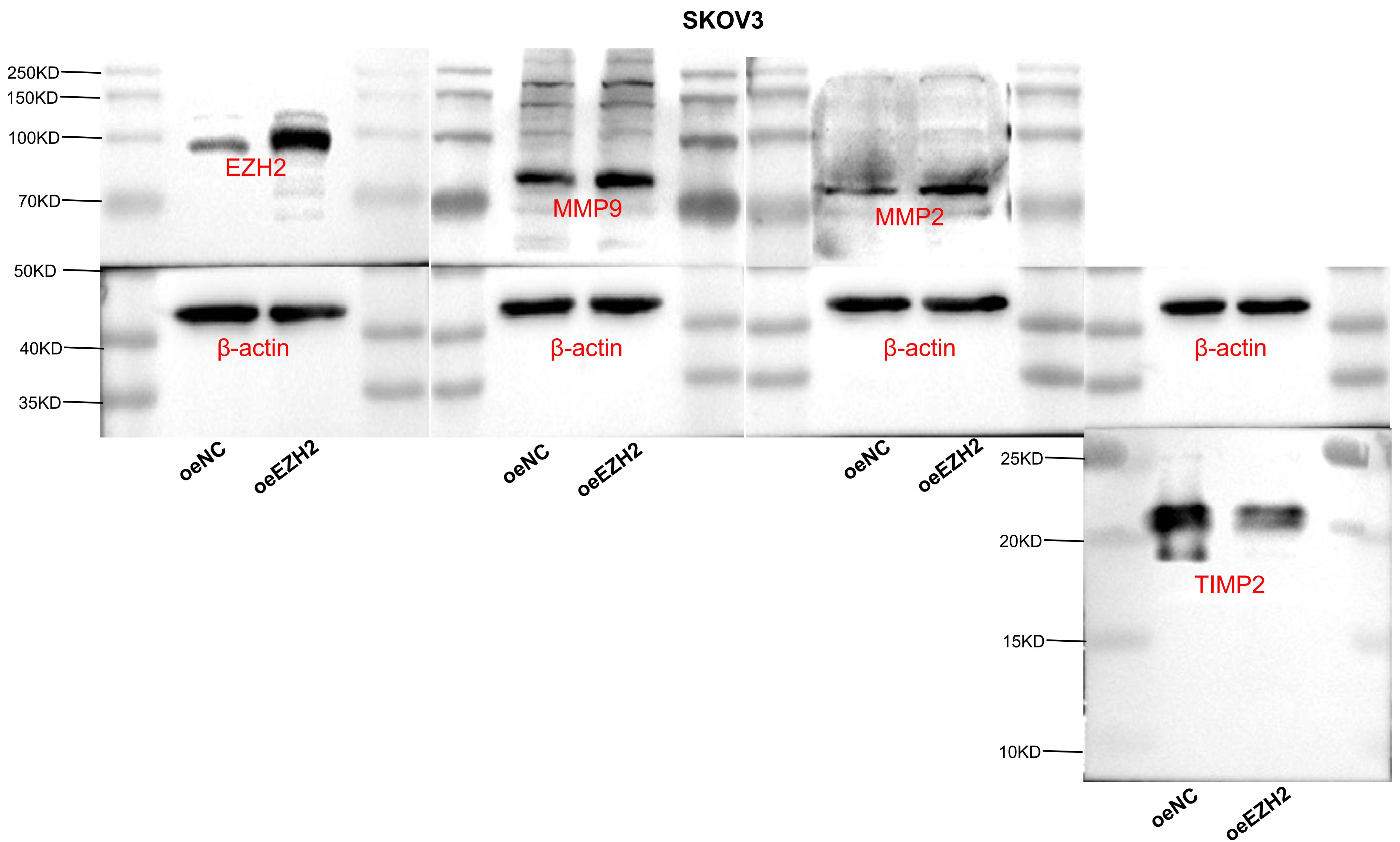

Supplementary Fig. 3C

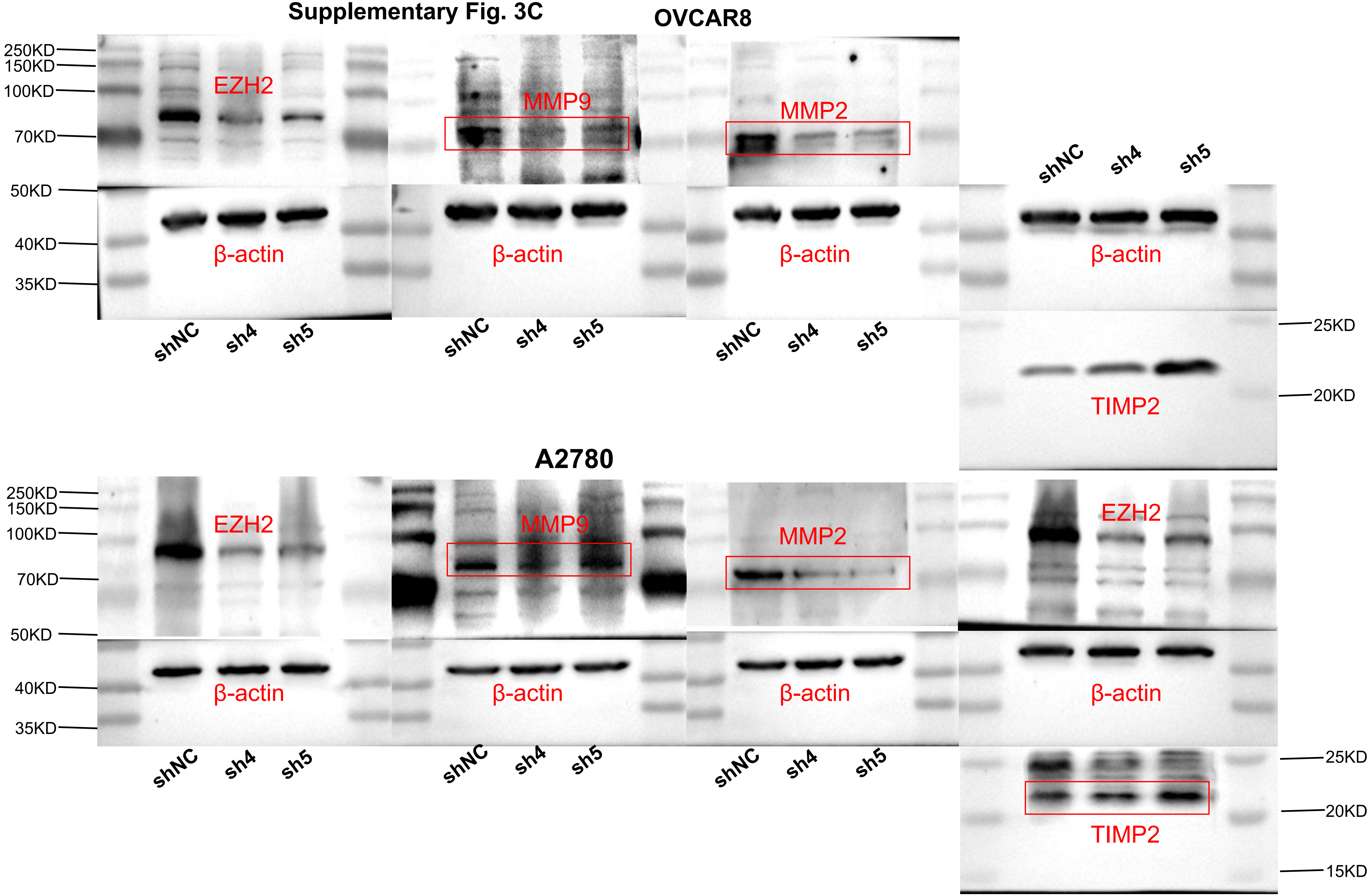

Supplementary Fig. 3D

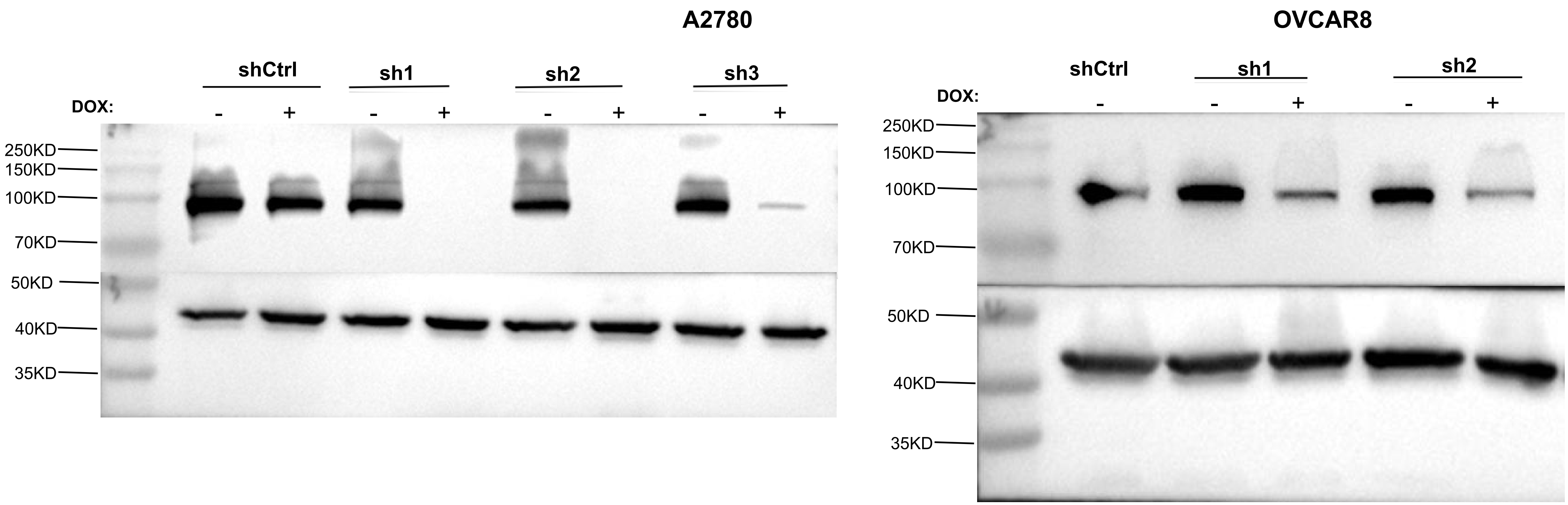

Supplementary Fig.4 D

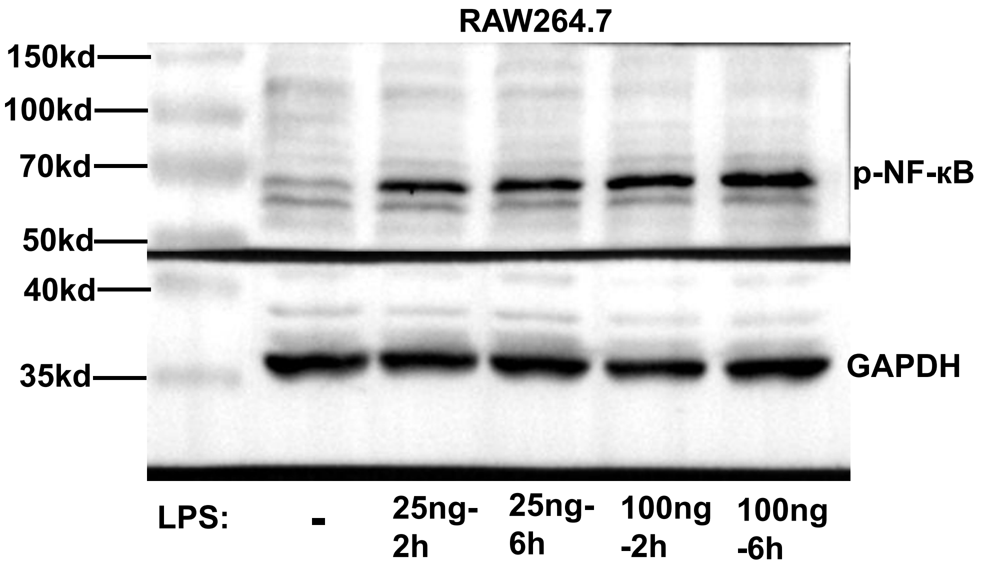

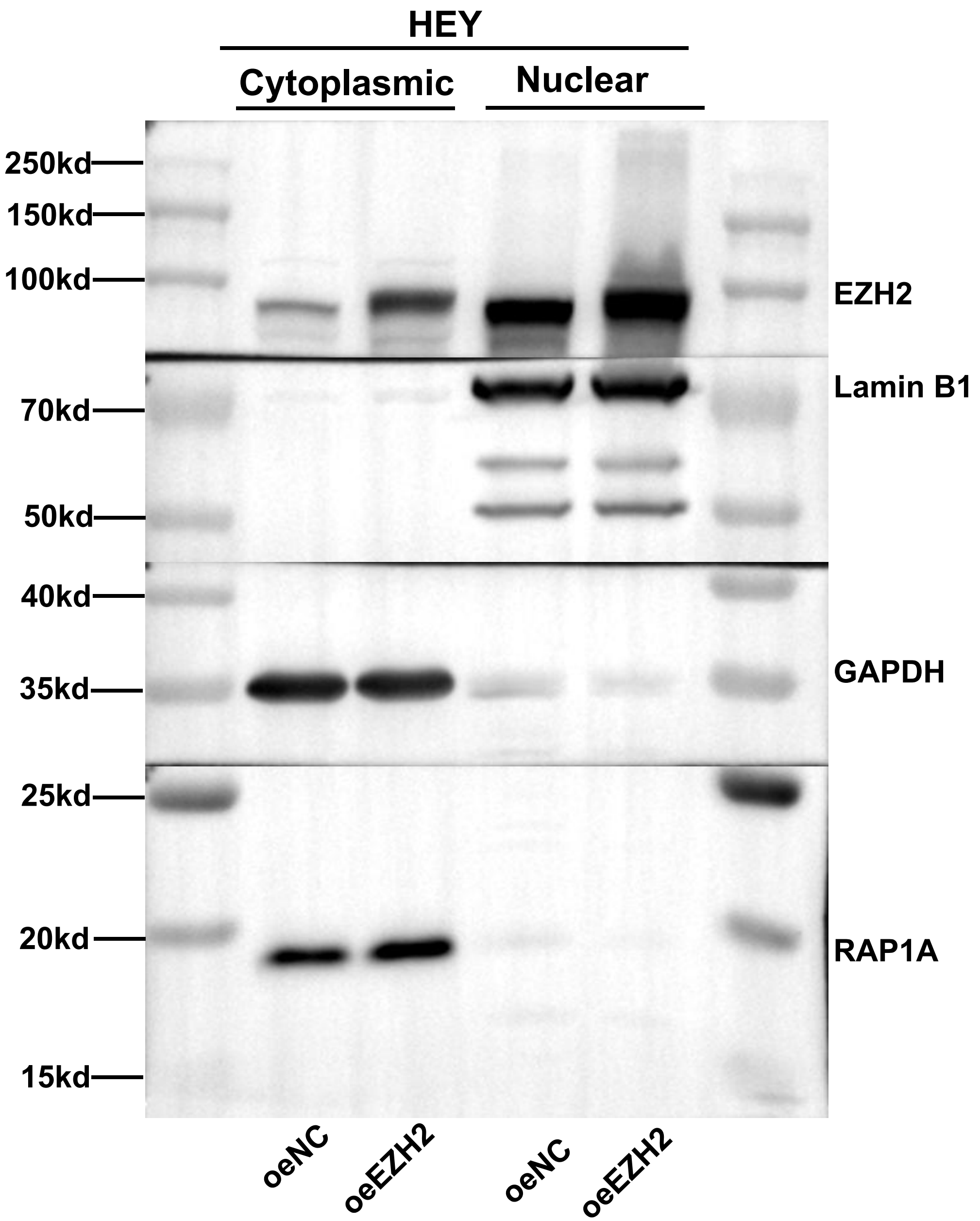

Supplementary Fig.4 D

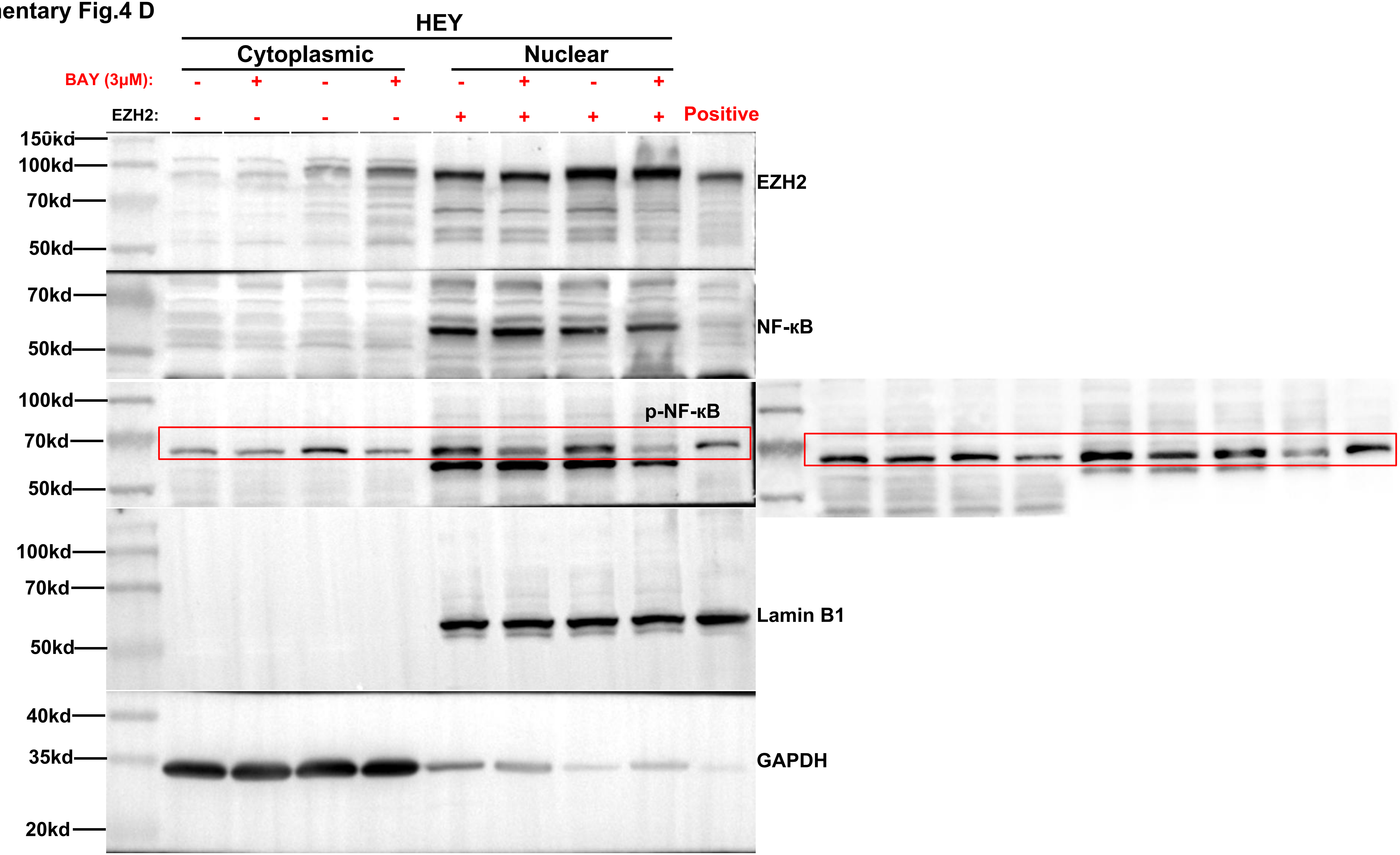

Supplementary Fig.4E

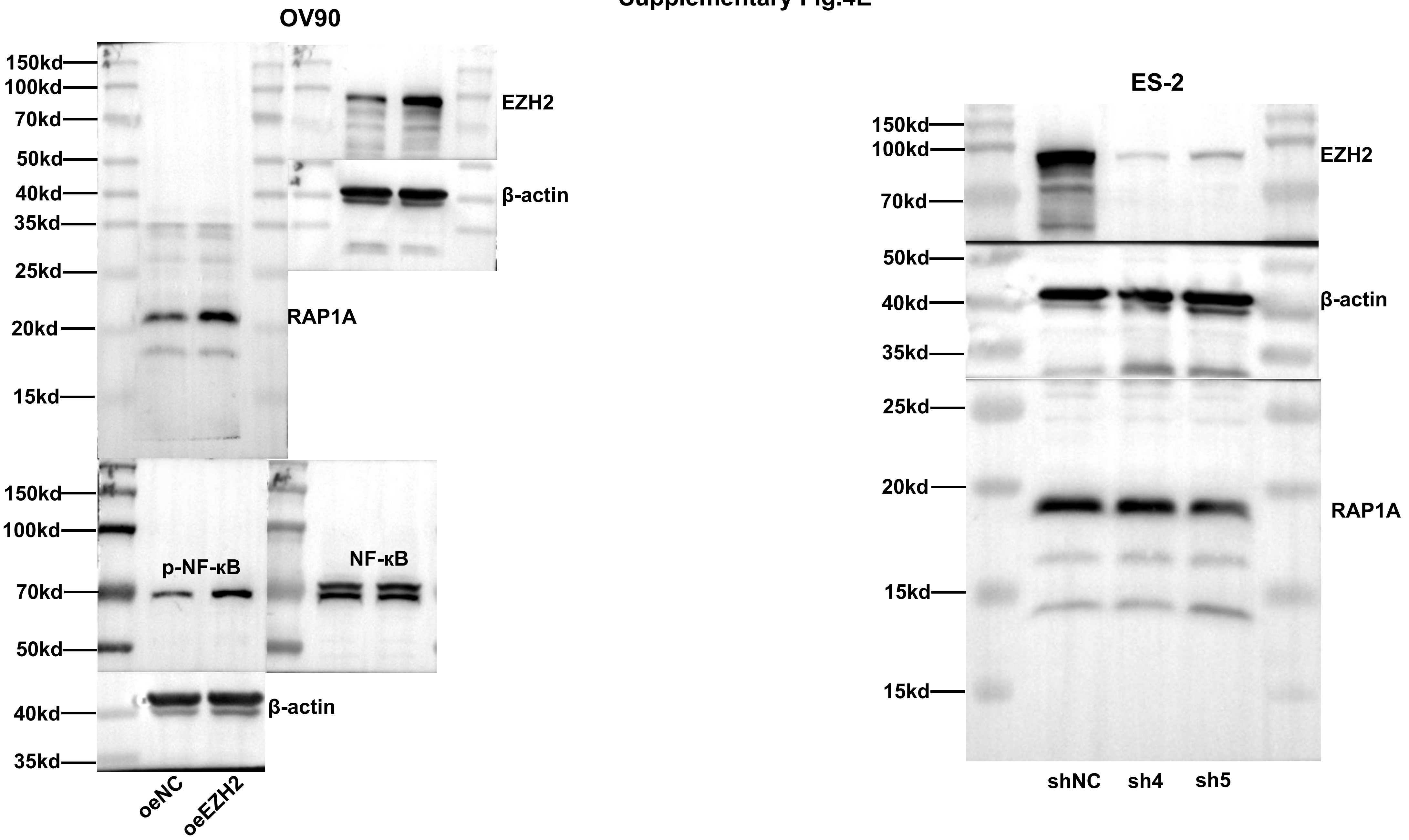

Supplementary Fig. 4F

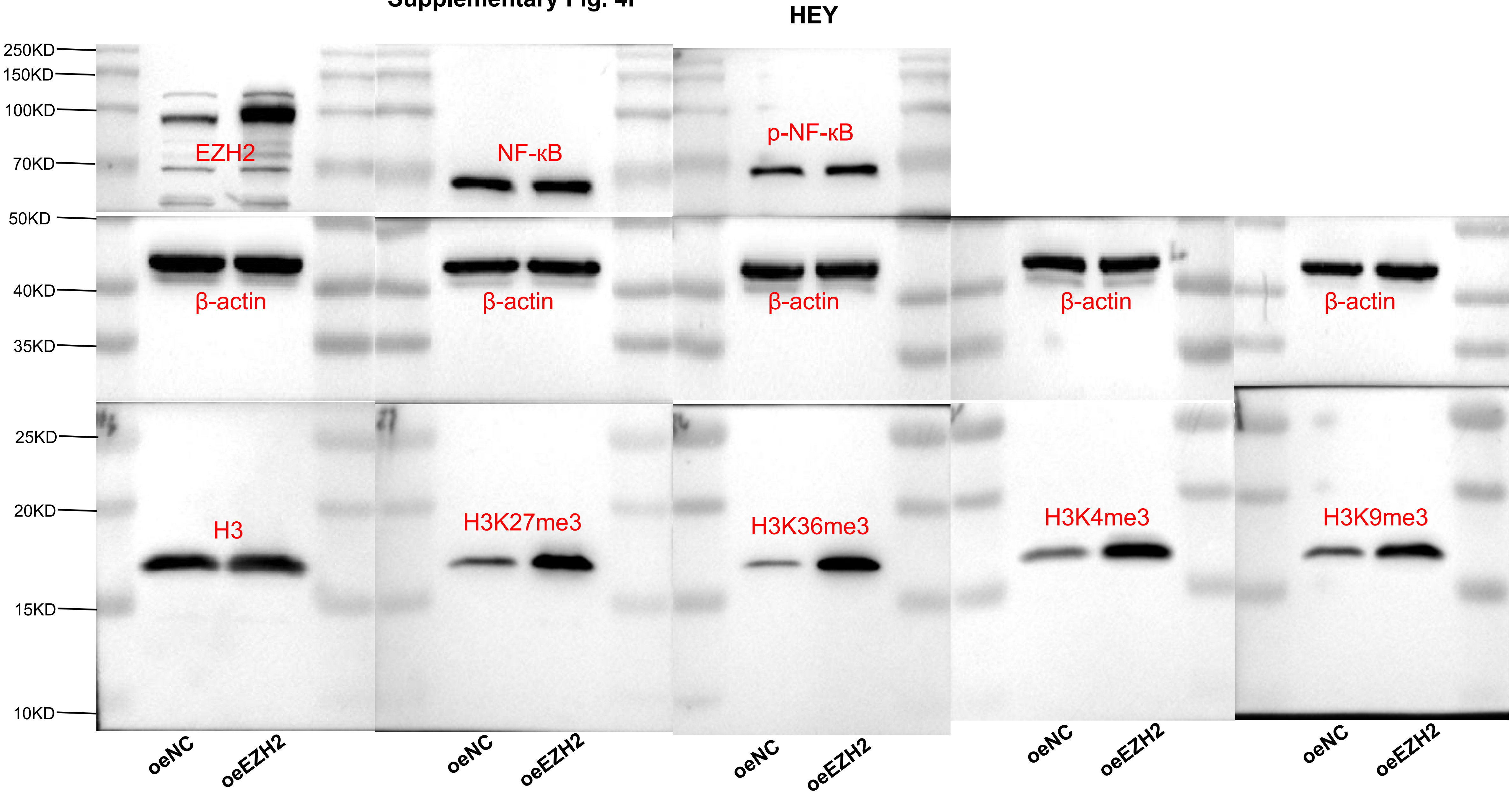

Supplementary Fig. 4F

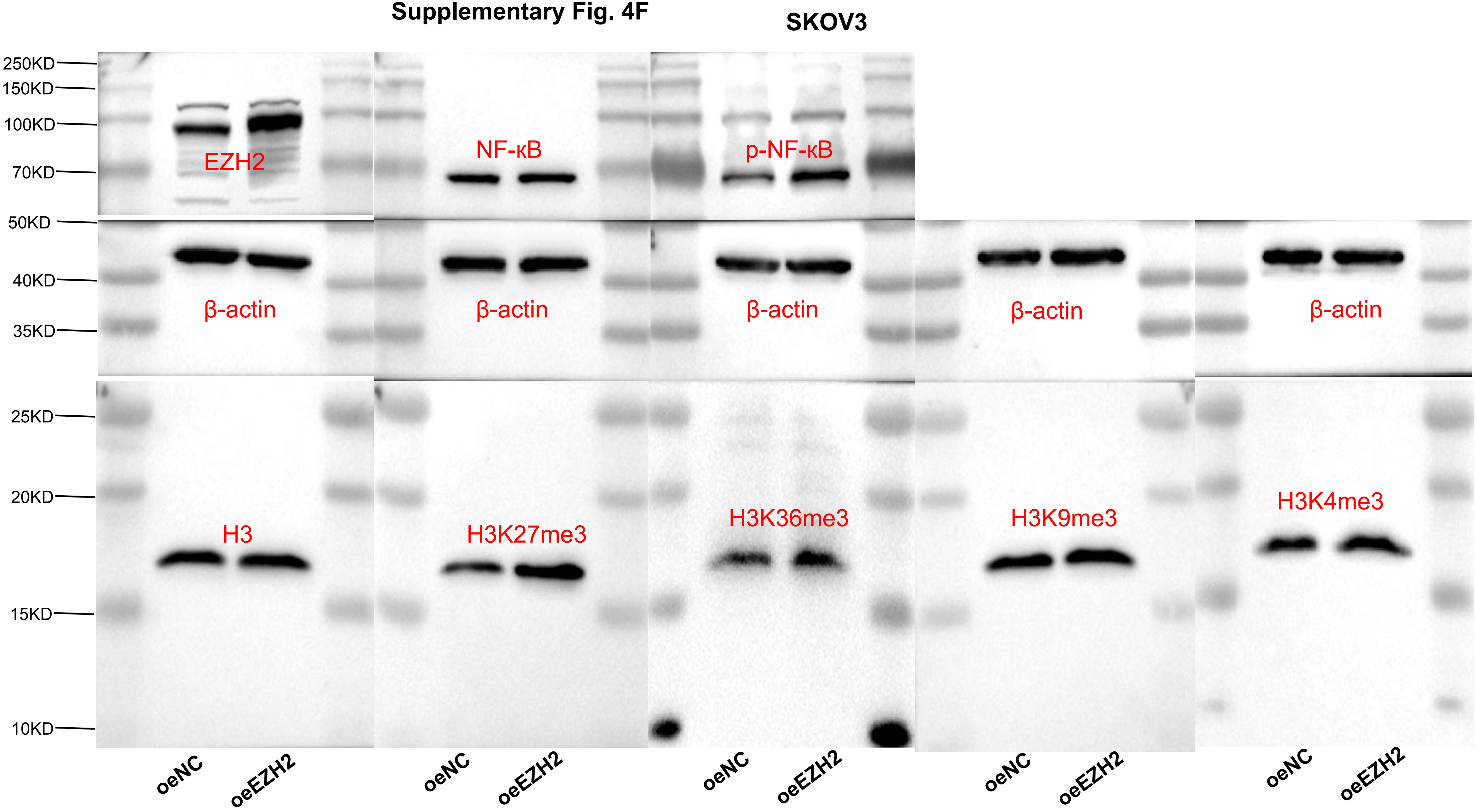

Supplementary Fig. 4F

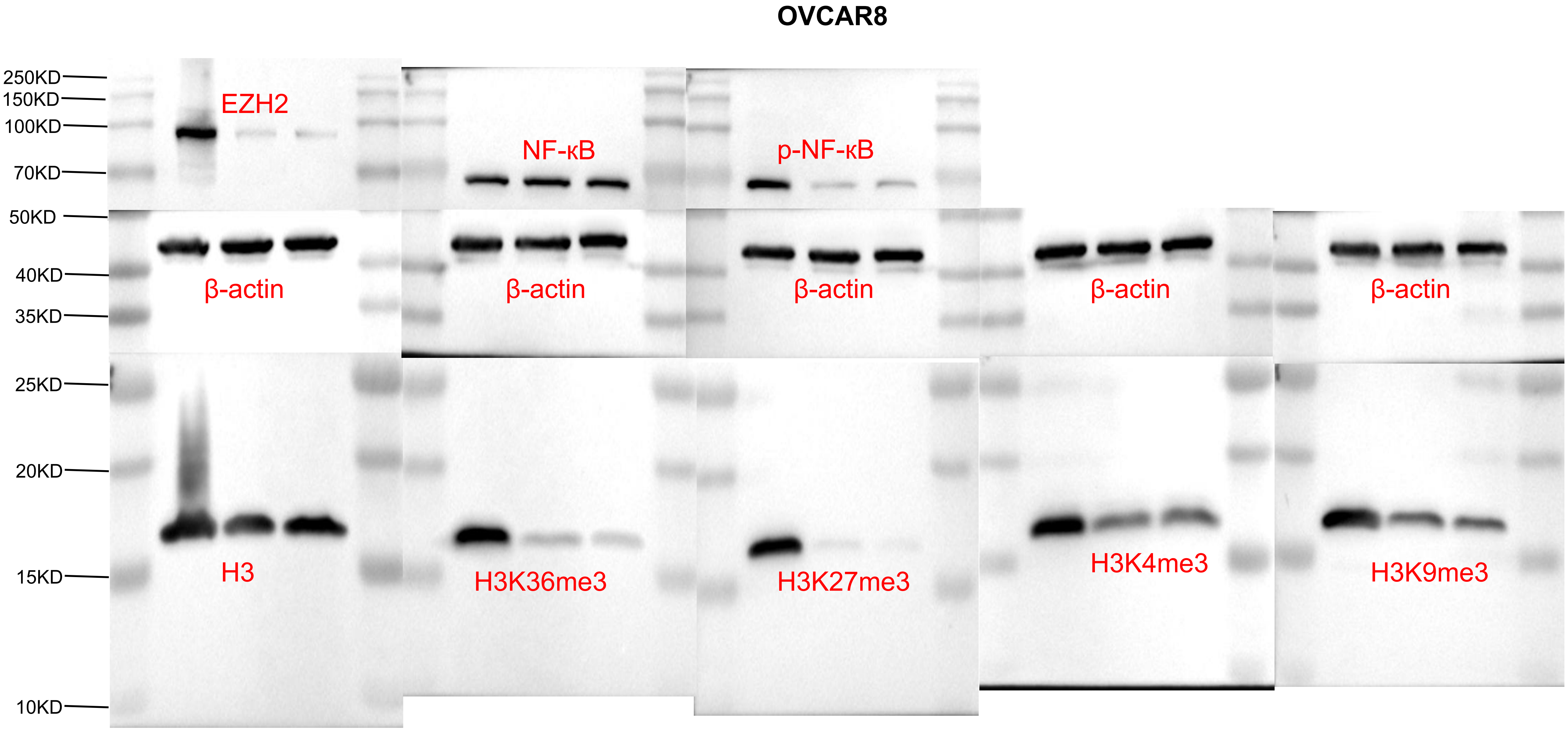

Supplementary Fig. 4F

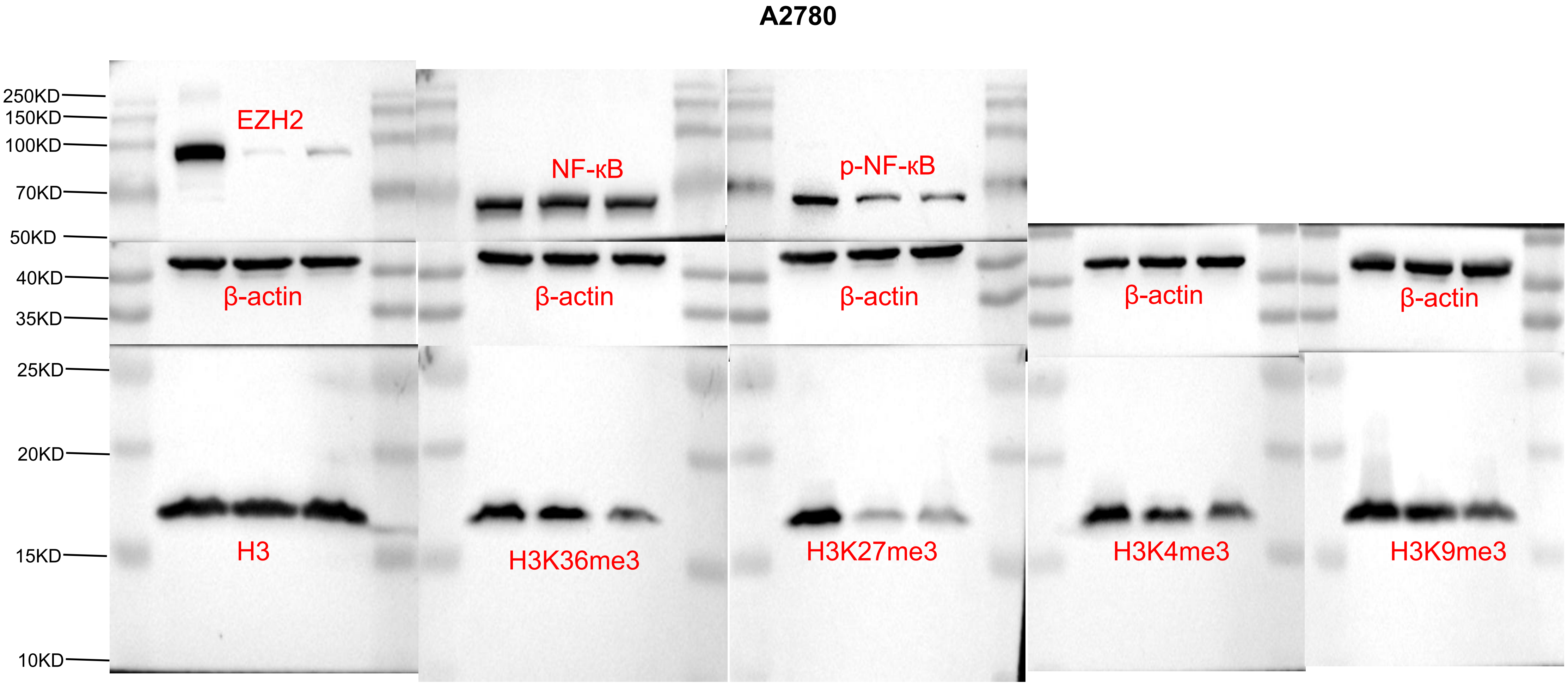

Supplementary Fig.4 I

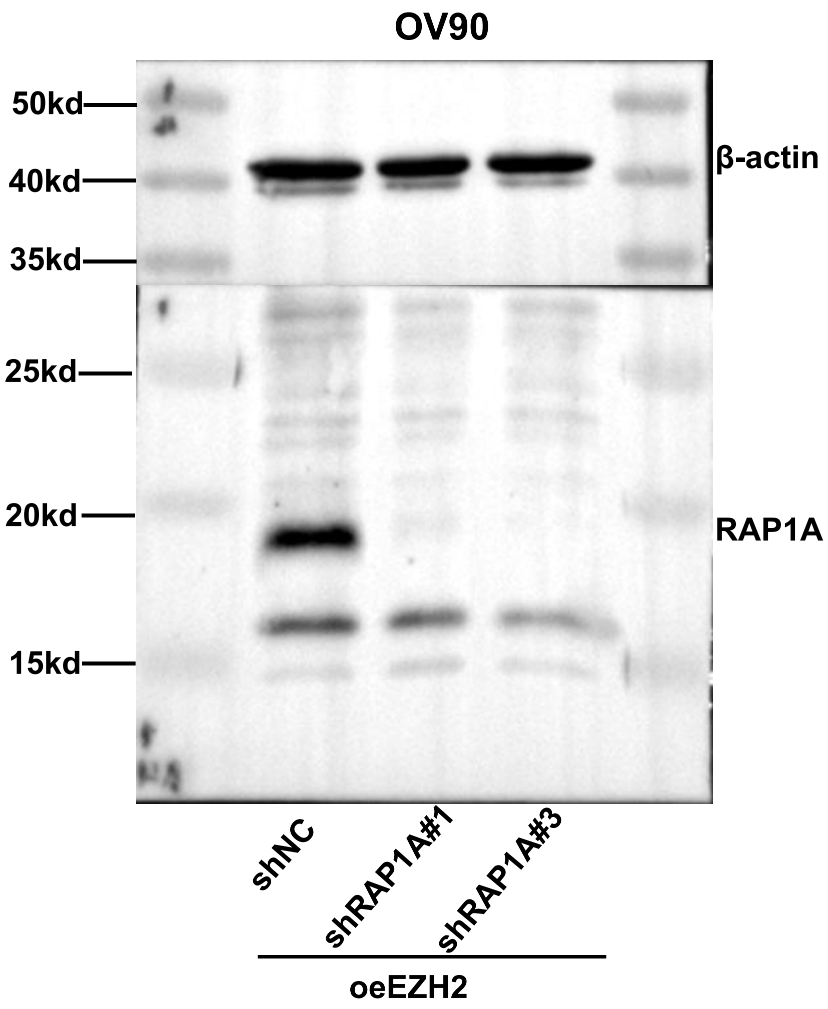

Supplementary Fig.6 F

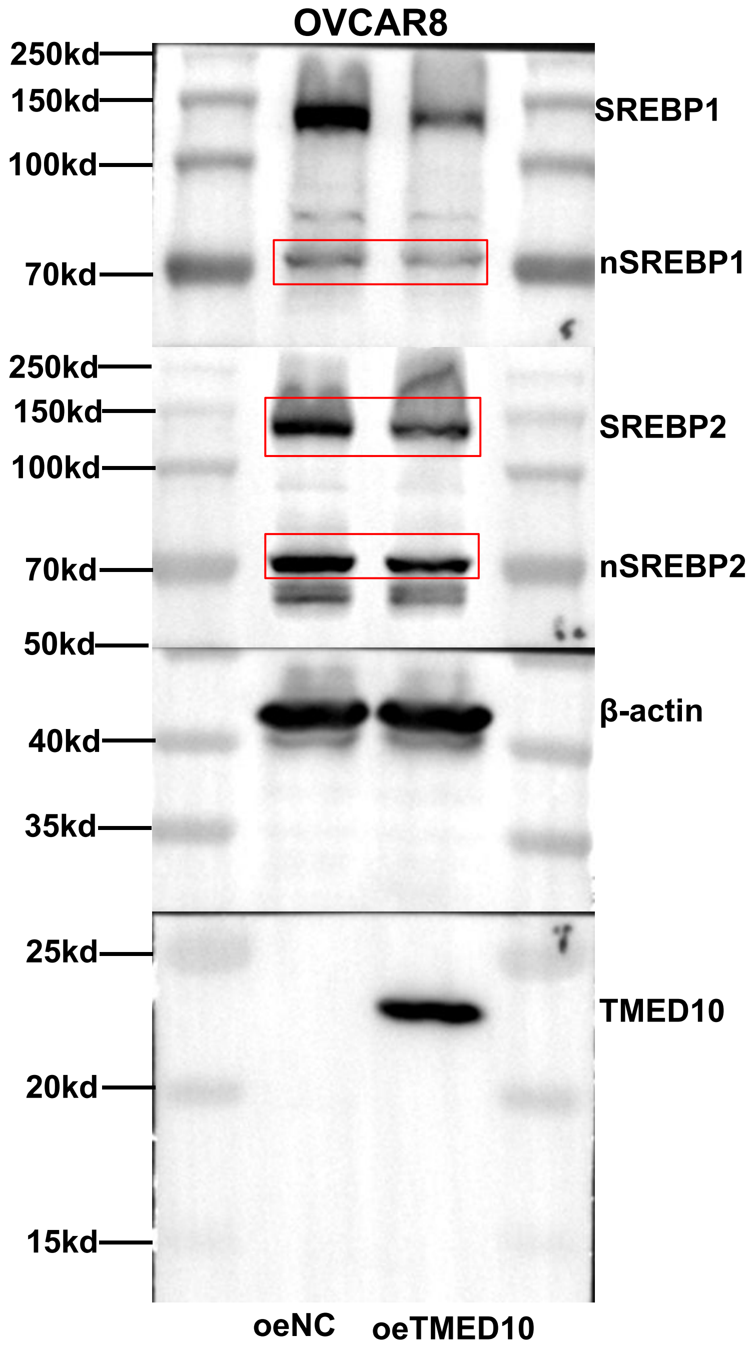

Supplementary Fig.6 G

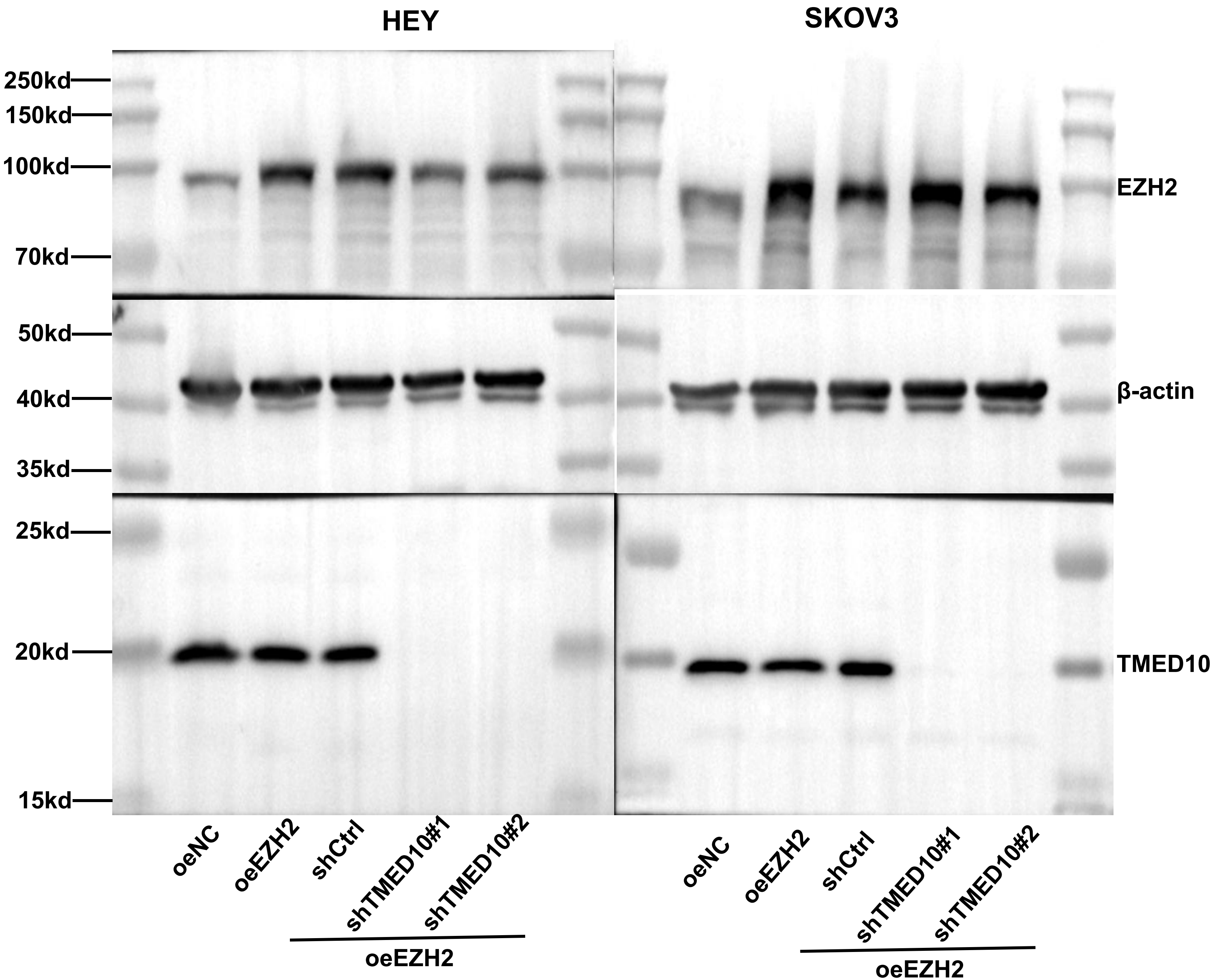

Supplementary Fig.7 D

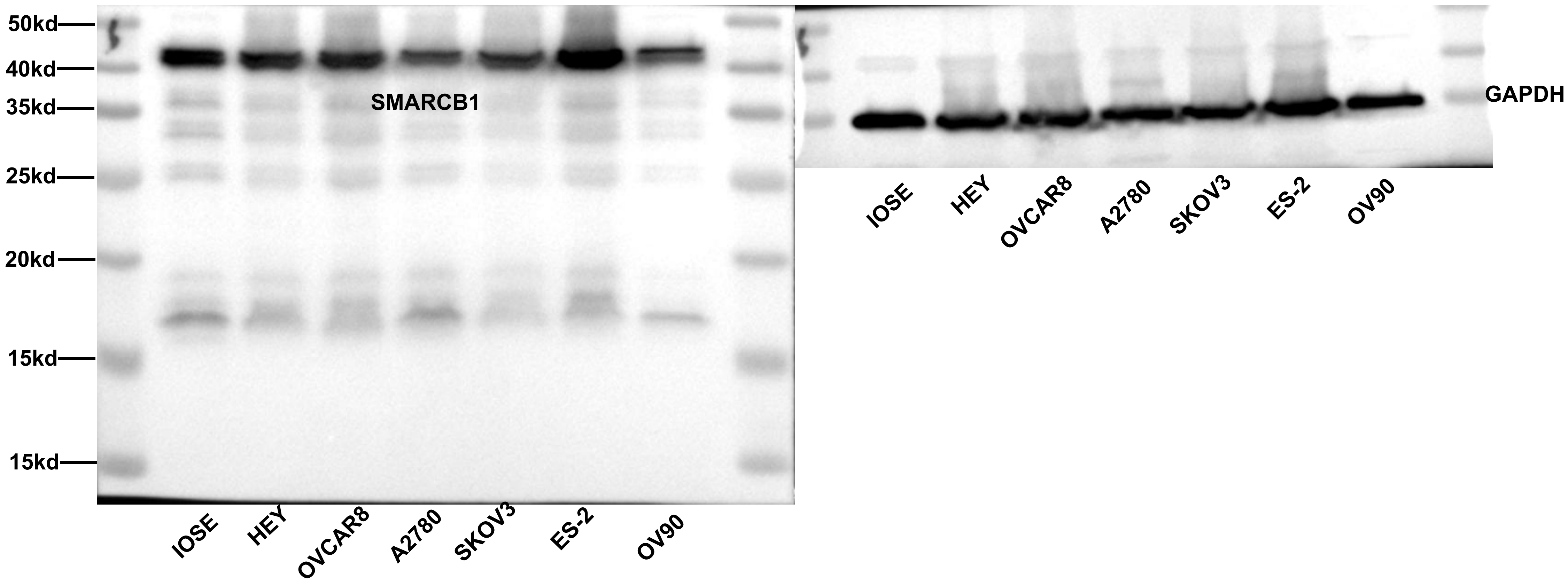

Supplementary Fig.7 E

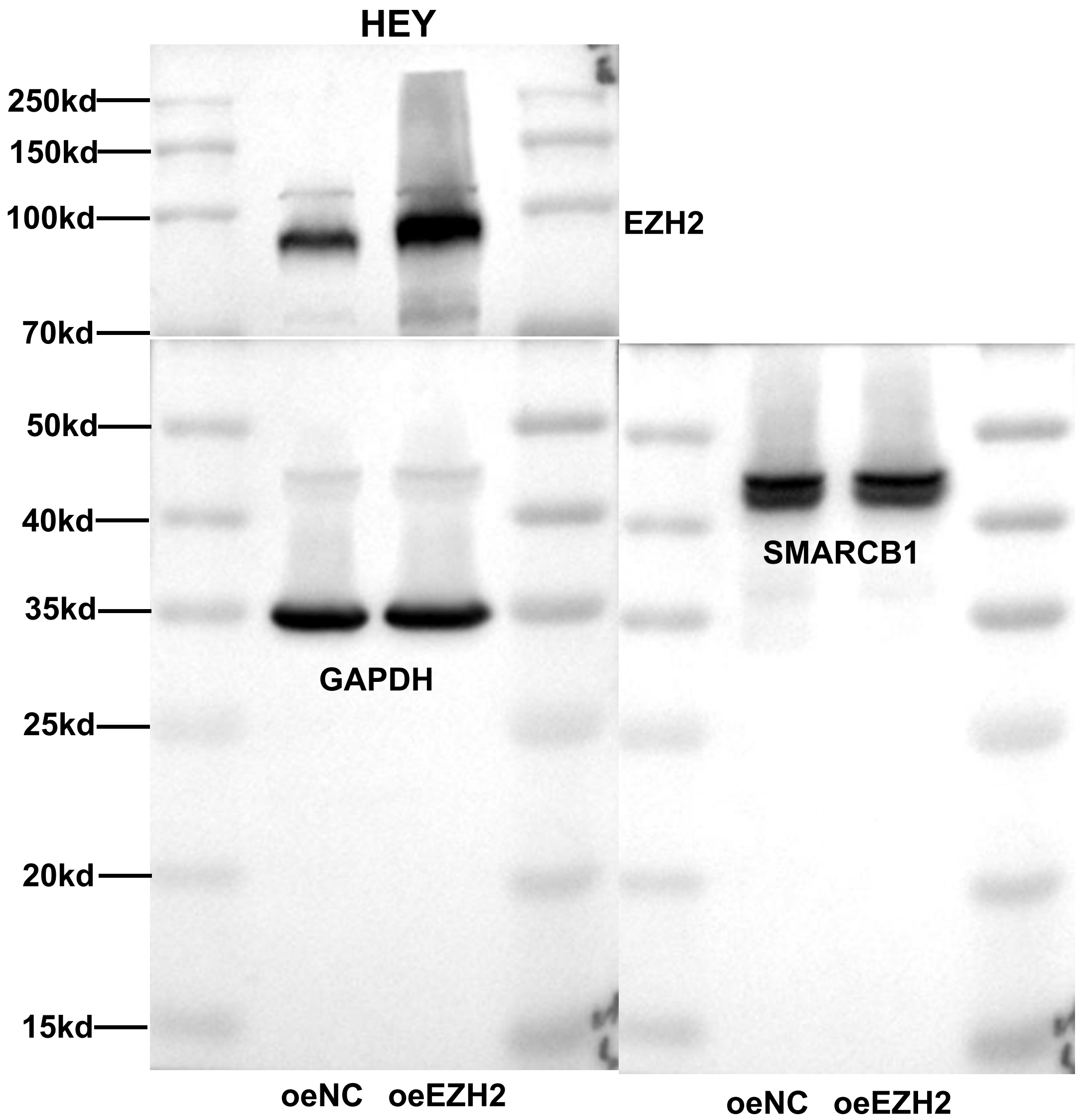

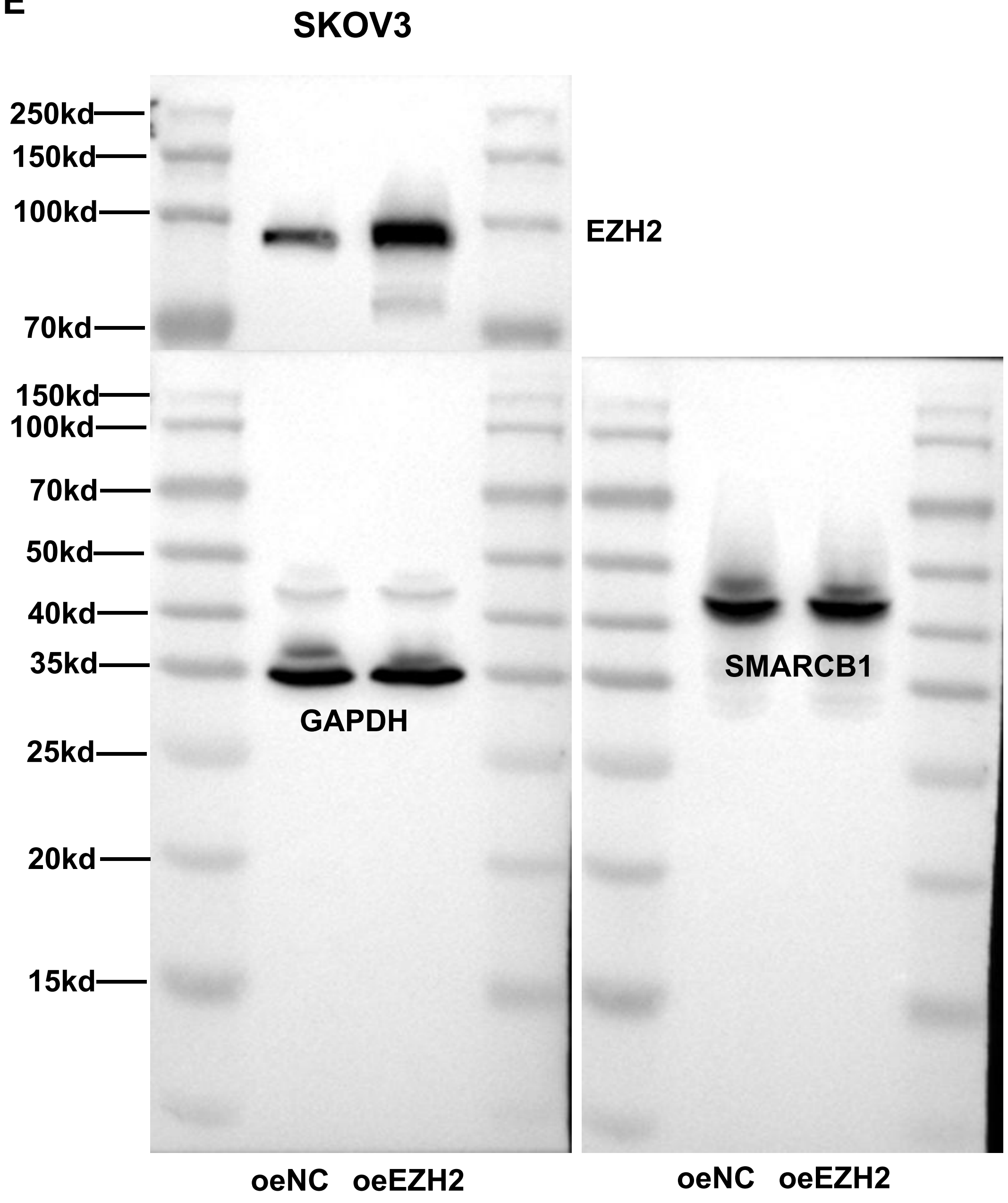

Supplementary Fig.7 F

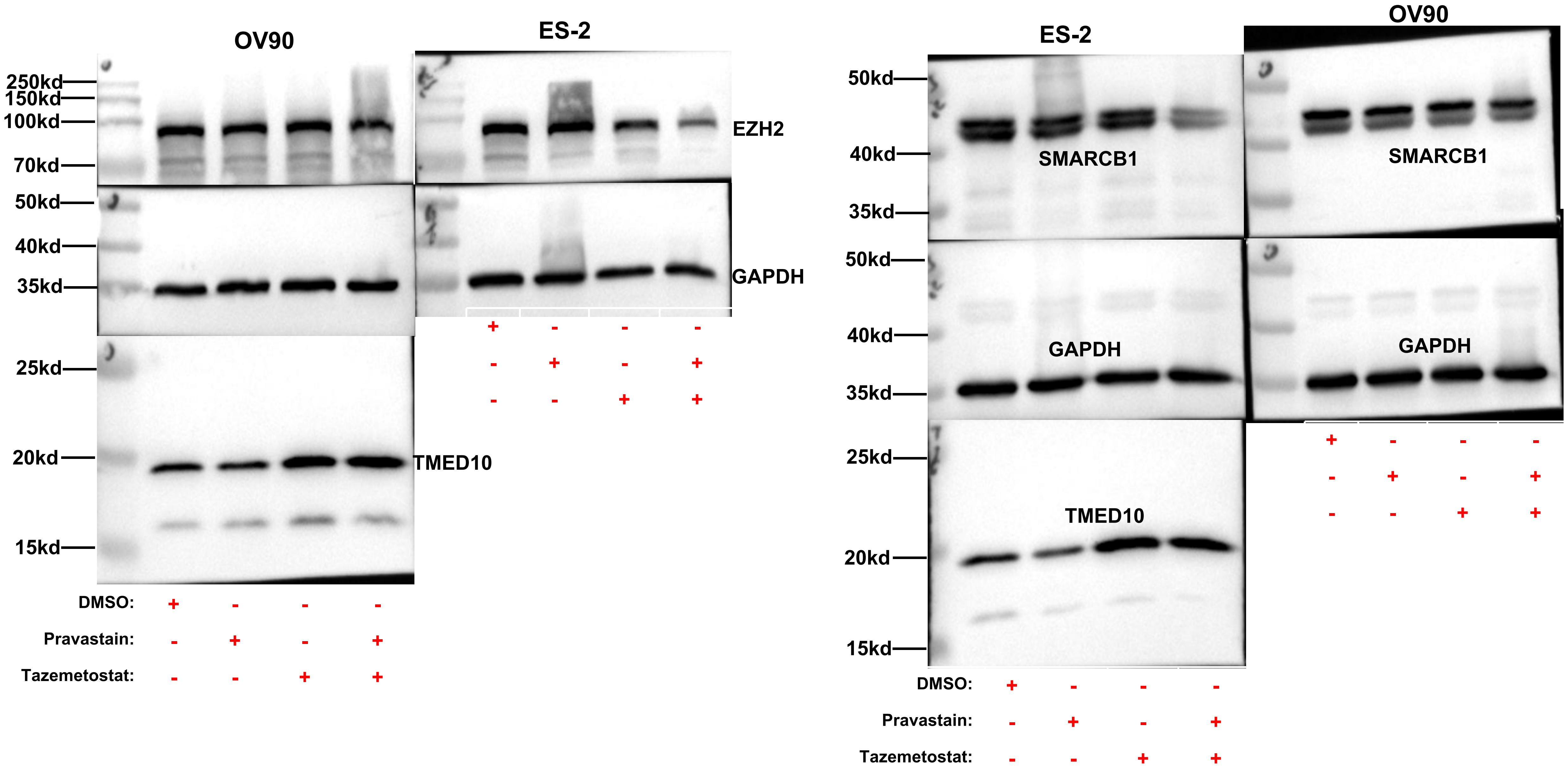

Supplementary Fig.8 D

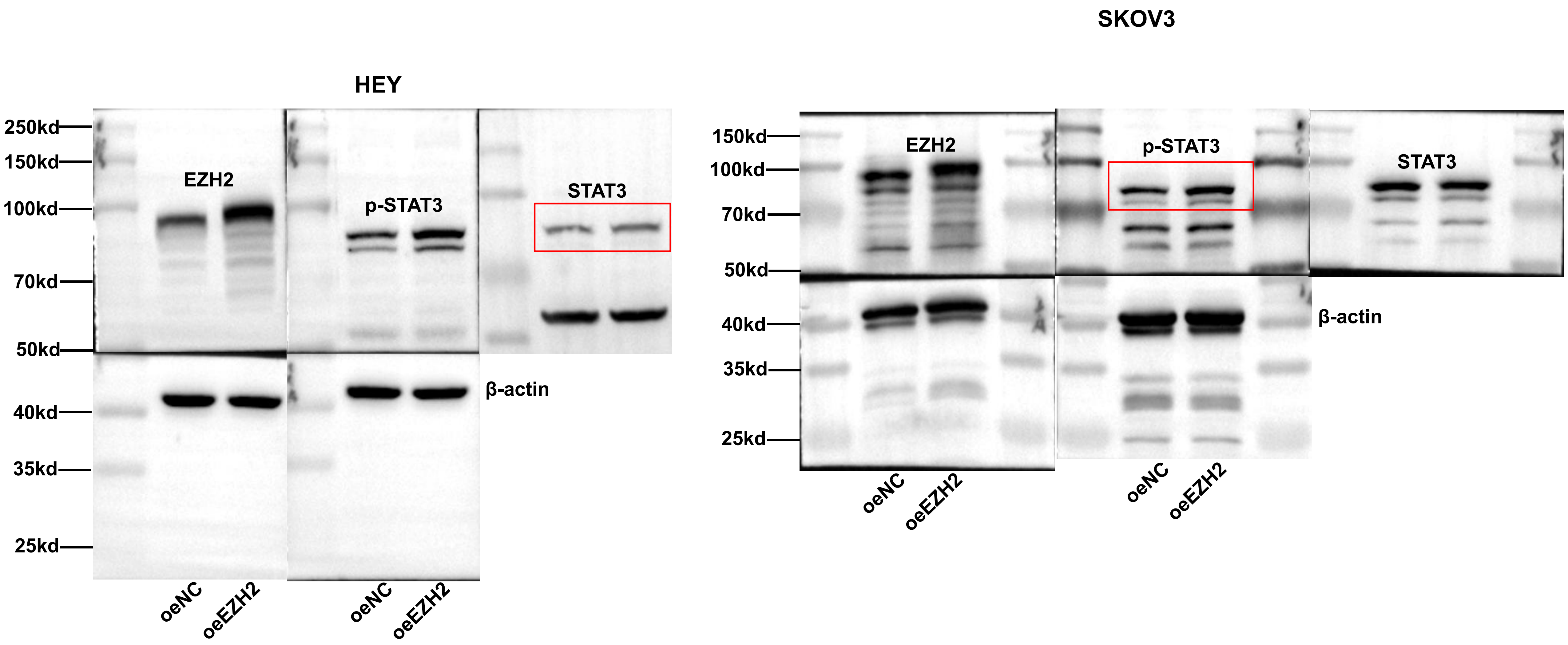

Supplementary Fig.8 G

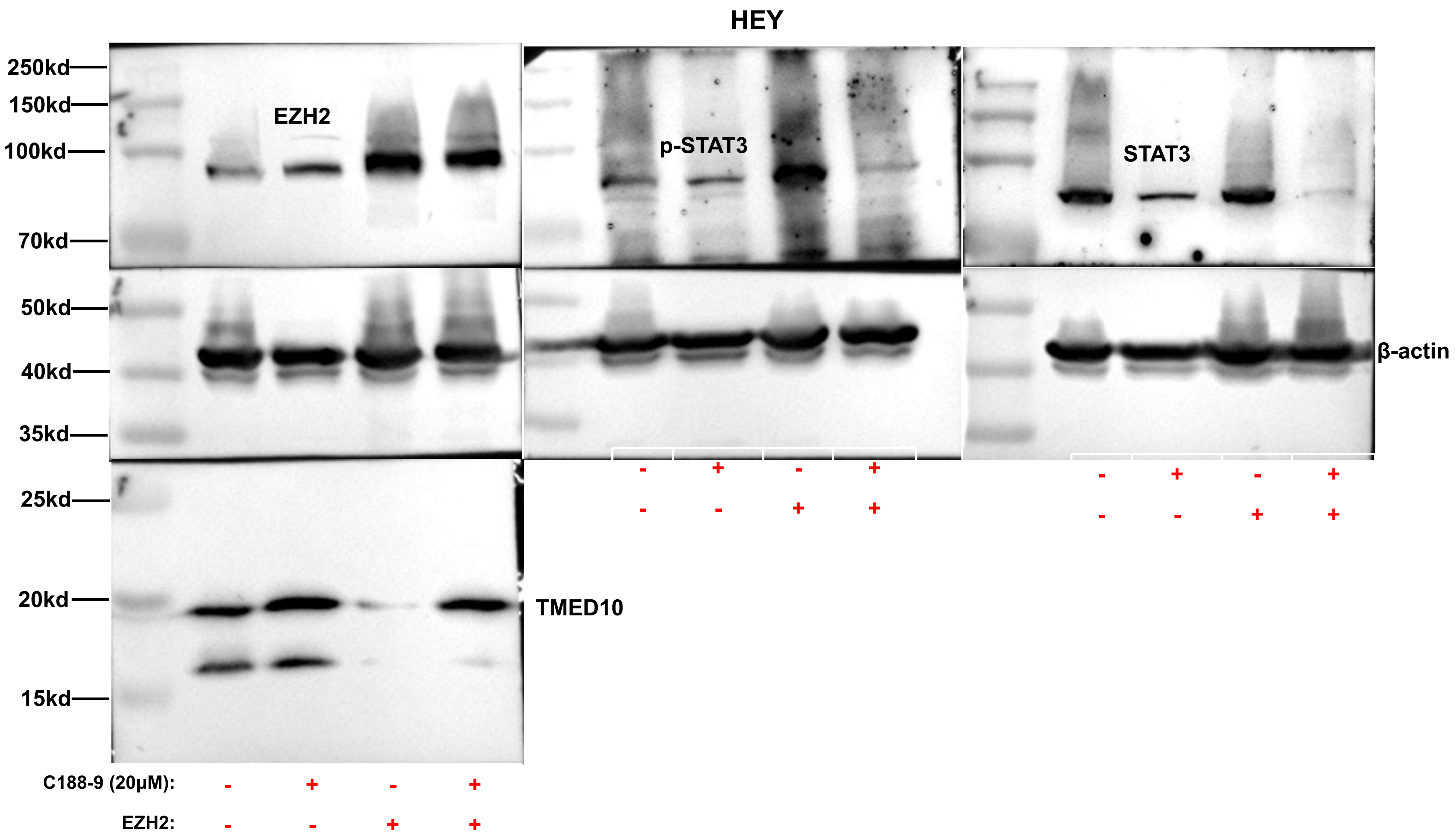

Supplementary Fig.8 H

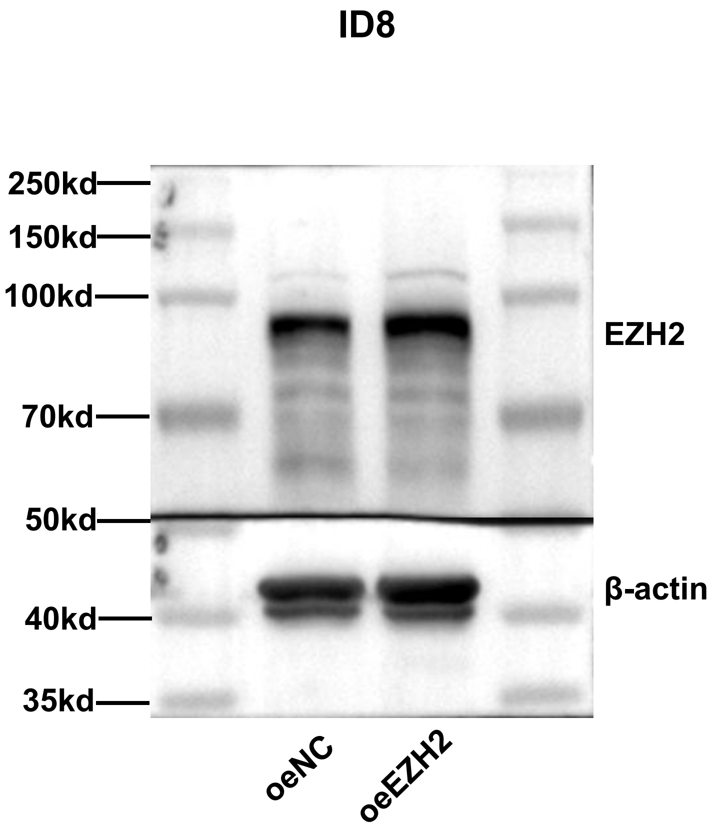

Supplement: Supplementary file 11 — Original Data [file 41419_2026_8894_MOESM11_ESM.pdf]
